# A defined microbial community reproduces attributes of fine flavour chocolate fermentation

**DOI:** 10.1038/s41564-025-02077-6

**Published:** 2025-08-18

**Authors:** David Gopaulchan, Christopher Moore, Naailah Ali, Darin Sukha, Sergio Leonardo Florez González, Fabio Esteban Herrera Rocha, Ni Yang, Mui Lim, Tristan P. Dew, Andrés Fernando González Barrios, Pathmanathan Umaharan, David E. Salt, Gabriel Castrillo

**Affiliations:** 1 Future Food Beacon of Excellence, University of Nottingham, Sutton Bonington Campus, Nottingham, UK; 2 School of Biosciences, University of Nottingham, Sutton Bonington Campus, Nottingham, UK; 3 School of Life Sciences, University of Nottingham, Nottingham, UK; 4 Cocoa Research Centre, The University of the West Indies, St Augustine, Trinidad and Tobago; 5 CasaLuker S.A., Bogotá, Colombia; 6 Grupo de Diseño de Productos y Procesos (GDPP), Departamento de Ingeniería Química y de Alimentos, Universidad de los Andes, Bogotá, Colombia

**Keywords:** Applied microbiology, Microbiome

## Abstract

Cocoa (*Theobroma cacao* L.) bean fermentation is a spontaneous process involving interactions between abiotic and biotic factors that contribute to the final flavours of chocolate. Understanding these underlying interactions could enable desired flavour profiles to be reproduced under controlled conditions. Here, using bean fermentation samples from Colombian farms, we established that pH, temperature and microbiota composition, including both bacteria and fungi, influence key flavour attributes of premium chocolate. Genome-resolved metagenomics revealed that metabolic traits necessary for the development of the flavour profile of chocolate are redundantly present in the fermentation microbial community. Using a defined and metabolically competent microbial consortium, the feasibility of replicating fine flavour attributes of chocolate under controlled conditions was confirmed via omics, metabolic networks and a trained tasting panel. Our results provide the basis for the design of fermentation starters to robustly reproduce fine chocolate characteristics.

## Abstract (web summary)

An in-depth microbiological and metagenomic analysis of Colombian farm and fermentation facilities resulted in the design of a defined microbial community that can reproduce the flavour of fine chocolate.

## Main

The production of fermented foods, such as chocolate, relies on the metabolic activities of microbial communities. Members of these communities transform the raw substrate, cocoa (*Theobroma cacao* L.) beans, into the precursor for chocolate production^1,2^. Although after harvest, cocoa beans undergo several processing steps to produce the final chocolate, the fermentation step, however, is a spontaneous process, involving a dynamic interplay between abiotic and biotic factors, resulting in the production of metabolites that define the chocolate flavour^3–9^. Unfermented cocoa beans develop little flavour potential when roasted and tend to be bitter and astringent^4,5^. In contrast, well-fermented beans can exhibit complex flavour notes with reduced bitterness and astringency. Consequently, the fermentation process is crucial for enhancing the aroma and flavour of the chocolate.

Typically, cocoa bean fermentation takes place on cocoa farms, within wooden boxes, baskets or heaps covered with various materials to regulate environmental temperatures^1,2^. In contrast to other fermented foods such as wine^10,11^, cheese^12^ or beer^13^, where microbes are often intentionally introduced or controlled, the microbial communities in cocoa bean fermentation assemble spontaneously from the environment with minimal human intervention. Therefore, variations in the assembly of microbial communities for fermentation across different locations are expected^14–16^, and this can influence the chocolate quality and flavour from different cocoa origins.

Although various approaches have been used to analyse microbial communities in fermenting cocoa beans^17–25^, our understanding of microbial community assembly during cocoa bean fermentation remains limited. Particularly, there is a knowledge gap regarding how abiotic and biotic factors influence fermentation outcomes and impact chocolate flavour^26^. This lack of knowledge limits the optimization of cocoa bean fermentation as an industrial process similar to other fermented food industries. Therefore, further studies are imperative to delve deeper into this system to unlock its full potential.

## Results

### Changes in temperature and pH inform fermentation progress

**Fig. 1:**
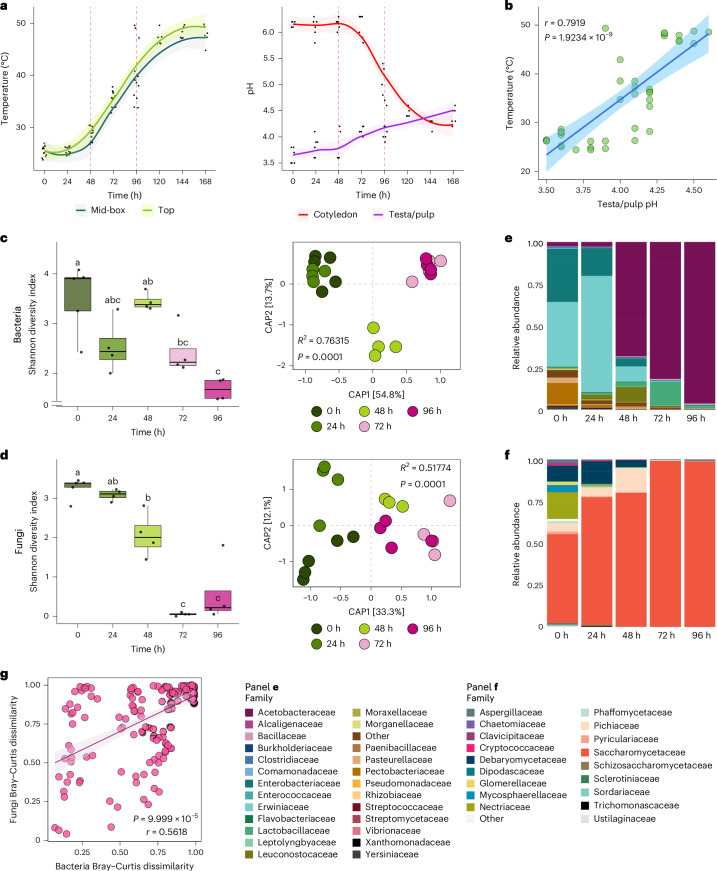
Changes in abiotic and biotic characteristics inform the progression of cocoa bean fermentation. **a**, Temperature and pH changes of the fermenting beans during fermentation. Shaded areas represent 95% confidence intervals (CIs). Temperature recordings were taken at two depths: below the surface (top) and midway through the fermenting mass (mid-box). The red dashed lines indicate the times when the beans were turned. **b**, Pearson correlation analysis (two-sided) between the bean temperature and testa/pulp pH. Shading represents 95% CIs. The Pearson correlation coefficient (*r*) and the associated *P* value are shown. **c**,**d**, Alpha and beta diversity estimates of the bacterial (**c**) and fungal (**d**) communities in the fermentation at different time points. Microbial samples were collected from two independent fermentations. A total of 21 bacterial and 20 fungal community profiles were used. Alpha diversity was assessed using the Shannon diversity index. The horizontal line within each box represents the median; box edges represent the interquartile range from the 25th to the 75th percentiles; whiskers extend to the smallest and largest values within 1.5× the interquartile range from the lower and upper quartiles; individual data points, including outliers, are overlaid as dots. One-way ANOVA was used to discern significant differences among groups (bacteria: *P* = 4.2759 × 10^−4^; fungi: *P* = 1.9382 × 10^−7^) and means were separated using Tukey’s post hoc test. Shared letters denote no significant difference (*P* > 0.05), whereas different letters indicate statistically significant (*P* < 0.05) variation between groups. Beta diversity was visualized using canonical analysis of principal coordinates (CAP). Group differences were assessed using PERMANOVA with 9,999 permutations. PERMANOVA *R*^2^ and *P* value are displayed. **e**,**f**, Phylogram showing the relative abundance profiles of the main bacterial (**e**) and fungal (**f**) families at different fermentation time points. The proportions in the bars represent the average relative abundances of each taxon, calculated across multiple replicate samples for the bacterial (*n*_total_ = 21) and fungal (*n*_total_ = 20) communities. **g**, Mantel correlation between bacterial and fungal Bray–Curtis dissimilarity matrices. The correlation was assessed using a two-sided Mantel test with the Pearson method and 10,000 permutations. Shading indicates the 95% CI of the fitted regression line. The Mantel correlation coefficient (*r*) and corresponding *P* value are displayed.

**Extended Data Fig. 1:**
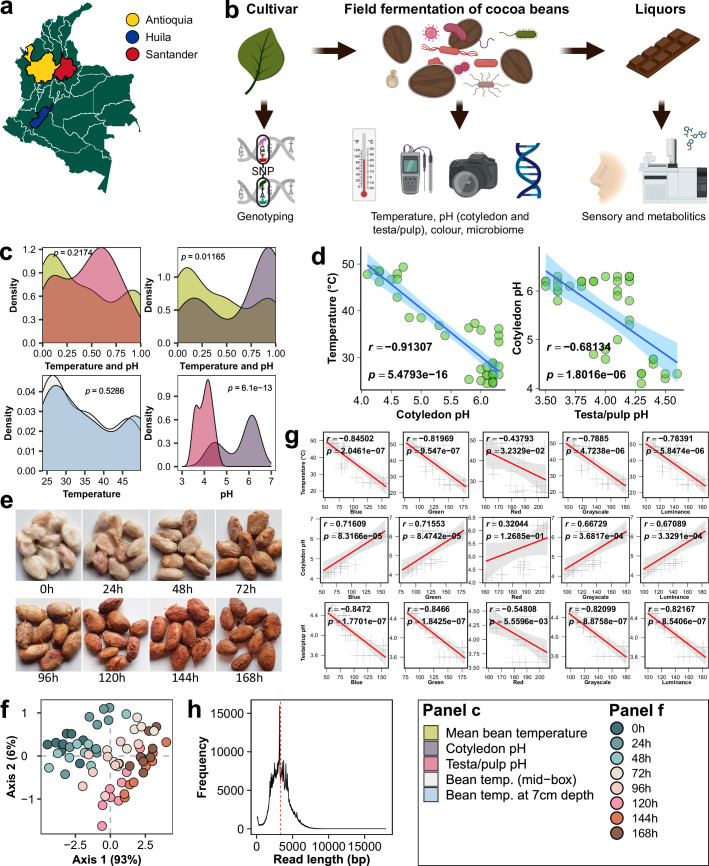
Temperature and pH changes predict the end of cocoa bean fermentation. **a**. The map shows the cocoa plantations from three cocoa-growing regions in Colombia (Santander, Huila, and Antioquia) evaluated in this study. **b**. Schematic diagram of field bean fermentation analysis performed in this work. Cocoa varieties grown on farms were genotyped using SNP analysis. Temperature, pH, and colour changes in the fermenting beans were recorded daily. Microbial communities were characterised using Oxford Nanopore metagenomic shotgun sequencing. After fermentation and drying, beans were processed into cocoa liquors and analysed through sensory evaluation and metabolomics. The diagram was created in BioRender. Gopaulchan, D. (2025) https://BioRender.com/t56v367**c**. Line graphs depict the magnitude of distribution of bean temperature and pH recorded during the fermentation. The difference between distributions was assessed using a Kolmogorov-Smirnov test, with corresponding *p*-values indicated within the plots. **d**. Pearson correlation analysis between the temperature and pH of the bean cotyledon and between the pH of the bean cotyledon and the pH of the testa/pulp compartment. A linear regression line illustrates the relationship, with a shaded band representing the 95% confidence interval. The scatter plots display correlation coefficients (*r*) and associated *p*-values. **e**. The images show the daily appearance of the cocoa bean, from the beginning (0 h) to the end (168 h) of the fermentation. **f**. Principal component analysis (PCA) of bean colour changes during fermentation. PCA is based on extracted RGB values from images of the beans taken daily. A minimum of eight points on each bean image were analysed for each time point. **g**. Pearson correlation analysis between bean colour parameters (blue, green, red, grayscale, and luminance) and fermentation temperature, between the bean colour parameters and the pH of the bean’s cotyledon, and between the bean colour parameters and the pH of the testa/pulp pH. The relationship is illustrated by a linear regression line, with the shaded region indicating the 95% confidence interval. Correlation coefficients (*r*) and associated *p*-values are shown within each panel. **h**. Read length distribution of Nanopore sequencing reads. The dashed red line indicates the average read length of 3,326.4 bp.

We monitored abiotic factors, such as temperature and pH, during bean fermentations in Colombia over two growing seasons. We selected a farm in the Santander district, the country’s leading cocoa-producing region (Extended Data Fig. 1a). Temperature changes in fermenting beans were measured daily at two depths over 7 days (Extended Data Fig. 1b). We found that the temperature of fermenting beans increased after 24 h, exhibiting a sigmoidal pattern that resembled typical microbial growth curves (Fig. 1a). Therefore, we hypothesized that this temperature increase was associated with exothermic metabolic reactions in the bean pulp, probably driven by microbial activity.

To reinforce this hypothesis, we measured changes in pH in the beans’ testa associated with the pulp (Extended Data Fig. 1b). The bean pulp is rich in primary and secondary substrates that can be used as carbon sources by the microbiota present^3,5,19,27^. We observed that in the first 48 h of fermentation, the pH of the testa/pulp was low (pH < 4) (Fig. 1a). Subsequently, the pH increased linearly probably due to chemical transformations of substrates in the testa/pulp^5^. In addition, we found a positive correlation between pH changes measured in the testa/pulp and temperature changes recorded in the fermenting cocoa mass (Fig. 1b). Both parameters exhibited similar magnitudes of variation during fermentation, indicating a link between changes in pulp pH and temperature shifts (Extended Data Fig. 1c). This is consistent with chemical transformations occurring within the pulp during fermentation^28,29^.

We next investigated pH changes in bean cotyledons, a measure more directly associated with bean fermentation (Fig. 1a). Contrary to the pH increase in the pulp, we found that the pH in the cotyledons decreases sigmoidally with time (Fig. 1a). A plausible explanation for this negative correlation between the pH in the testa/pulp and cotyledons (Extended Data Fig. 1d) could be that cocoa fermentation is a compartmentalized process that progresses simultaneously in the pulp and cotyledon. This compartmentalization could be facilitated by the physical barrier provided by the testa^30^ and the different chemical compositions of the pulp and cotyledon^3,27,31^. Supporting this hypothesis, we found that pH changes in the testa/pulp do not explain the pH shifts detected in the cotyledon (Extended Data Fig. 1c). Nevertheless, Pearson’s correlation analysis revealed a strong negative correlation (*r* = −0.91307, *P* = 5.4793 × 10^−16^) between bean temperature and pH changes in the cotyledon (Extended Data Fig. 1d). These results strongly support the idea that changes in temperature and pH during cocoa bean fermentation are the result of chemical reactions probably driven by the microbes present^8,19,32^. Overall, we conclude that bean temperature and cotyledon pH could serve as markers for bean fermentation progression. In fact, these parameters significantly correlated with colour changes in beans, which are traditionally used to assess the fermentation end point^5,33,34^ (Extended Data Fig. 1e–g).

### Changes in microbiota composition drive bean fermentation

To determine whether changes in microbial composition are associated with the progression of bean fermentation, we analysed microbial community dynamics using whole-metagenome shotgun Oxford Nanopore sequencing (Extended Data Fig. 1b,h and Supplementary Result 1). We found significant changes in alpha diversity (within-sample diversity) among microbial populations across different fermentation time points (Fig. 1c,d). Consistent with previous studies^14,35^, bacterial alpha diversity generally declined as fermentation progressed (Fig. 1c). Similarly, the alpha diversity of the fungal population followed a comparable declining trend across the fermentation time points (Fig. 1d). This decline in microbial diversity could be driven by metabolic changes in the pulp and bean composition because of microbial action^19,32,35^. These microbial activities may underlie the observed changes in temperature and pH during bean fermentation.

Analysis of beta diversity (between-sample variation) revealed that both bacterial and fungal communities separate along the first axis of a canonical analysis of principal coordinates (CAP) according to fermentation time (Fig. 1c,d), indicating clear shifts in community composition between early and late fermentation time points. In general, during the first 24 h of fermentation, the bacterial community was enriched in Erwiniaceae and depleted in Enterobacteriaceae and Pectobacteriaceae (Fig. 1e). Consistent with previous studies^1,2^, the bacterial community composition shifted after 48 h towards enrichment in Acetobacteraceae, which remained dominant for the rest of fermentation (Fig. 1e). In the case of fungi, we detected an initial enrichment in Saccharomycetaceae, aligning with previous reports^7,21,36–38^, and a concurrent depletion in Nectriaceae and Debaryomycetaceae within the first 24 h (Fig. 1f). The relative abundance of Saccharomycetaceae increased further at later time points during fermentation (Fig. 1f). We also analysed species-level enrichment of bacterial and fungal taxa across all fermentation time points relative to time zero (Supplementary Fig. 1 and Supplementary Result 2). Overall, these results underscore coordination between bacteria and fungi during bean fermentation (Fig. 1g) that may be influenced by changes in pH and temperature, spatial distribution and substrate availability. This proposed model of interkingdom microbial coordination could explain observed shifts in fermentation temperature and progressive acidification of the bean cotyledon, probably driven by the metabolic activities of Lactobacillaceae and Acetobacteraceae.

### Fermentation characteristics shape chocolate flavour profile

**Fig. 2:**
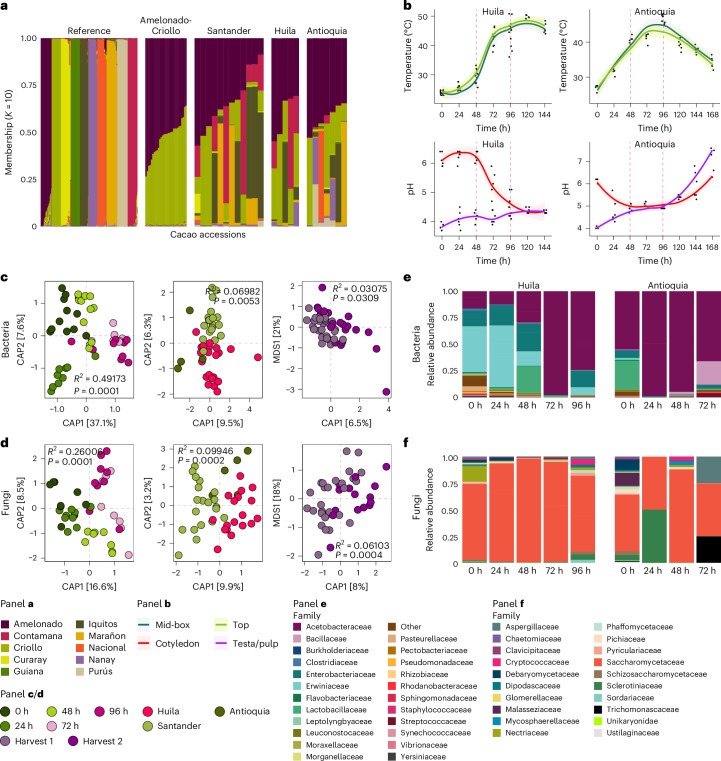
Cocoa bean fermentation attributes may influence chocolate flavour. **a**, Ancestry analysis of cultivated cacao plant samples from Santander (*n* = 12), Huila (*n* = 5) and Antioquia (*n* = 7). Population structure was determined on the basis of 84 high-quality SNP markers. The reference SNP profiles were primarily generated from cacao accessions at the International Cocoa Genebank Trinidad and were selected across the 10 cacao genetic clusters identified in ref. ^71^. In addition, Amelonado-Criollo reference hybrid accessions were included in the analysis. Each vertical line represents an individual. **b**, Temperature and pH changes of the fermenting beans in Huila and Antioquia farms. Shading indicates the 95% CI. Daily temperature recordings were taken at two depths within the fermentation box: 7 cm below the top surface (top) and midway through the fermenting mass (mid-box). Bean samples from these positions were collected and separated into testa with pulp and cotyledons, and the pH of both tissues was measured. Red dashed lines signify the time points when the beans underwent turning. **c**,**d**, Canonical analysis of principal coordinates (CAP) showing the bacteria (**c**) and fungi (**d**) community compositions in the fermenting beans across the three cocoa farms analysed in this study. Differences in beta diversity estimates are shown across fermentation time points (left), farm locations (middle) and bean harvesting periods (right). Group differences were assessed using PERMANOVA with 9,999 permutations. **e**,**f**, Phylogram displaying the relative abundance profiles of the main bacterial (**e**) and fungal (**f**) families at different fermentation time points. The proportions in the bar plots represent the average relative abundances of each taxon, calculated across multiple replicate samples for the bacterial (*n*_total_ = 24) and fungal (*n*_total_ = 23) communities.

To begin assessing the impact of the abiotic and biotic parameters on chocolate flavour, we selected two additional farms in Colombia in the regions of Huila and Antioquia (Extended Data Fig. 1a). First, we confirmed that the cocoa varieties from all three farms had comparable genetic backgrounds (Fig. 2a, Supplementary Fig. 2a,b, Supplementary Tables 1 and 2, and Supplementary Result 3). This analysis allowed us to exclude genotype as a relevant factor influencing bean fermentation and chocolate flavour in our study.

Next, we analysed the temperature and pH kinetics during bean fermentation at these two farms, across two consecutive harvests. Our findings showed that in general, the changes in these abiotic fermentation indicators in Huila, but not in Antioquia, resembled those observed previously at the Santander farm (Figs. 1a and 2b, Supplementary Fig. 2c and Supplementary Result 4). This suggests that the fermenting beans from Antioquia underwent chemical transformations distinct from those of the other two farms, probably due to the presence of different microbial communities.

To test this hypothesis, we characterized bacterial and fungal compositional changes in Huila and Antioquia fermentations (Extended Data Fig. 1h). Generally, the Huila and Antioquia fermentations showed microbiota characteristics similar to those in Santander (Figs. 1c,d and 2c,d, and Supplementary Fig. 2d). Supporting our hypothesis however, we found very different dynamics in the community in Antioquia (Figs. 1e,f and 2e,f, and Supplementary Result 5). These differences in microbial composition dynamics in Antioquia may explain the distinct temperature and pH patterns observed. Moreover, consistent with previous studies^14–16^, distinct differences in microbiota composition were detected across geographical locations, with a stronger signal in fungal populations (Fig. 2c,d, Supplementary Fig. 2d and Supplementary Result 5), suggesting that local environment or microbial source origin may influence fermentation characteristics. Consequently, we explored the influence of environmental microbial sources to the fermentation process (Supplementary Fig. 2e–i and Supplementary Result 6). Overall, the results suggest that bacteria harboured within fermentation box surfaces might function as a ‘memory’, serving as a stable inoculation source over time, whereas initial sources of fungi for fermentation probably originate from diverse environmental sources, explaining the stronger geographic signal. These divergent contributions from environmental sources among farms, coupled with temporal disparities, may enrich flavour diversity across locations.

We analysed whether changes in the identified fermentation signatures (temperature, pH and microbial composition) could offer insights into the quality of the fermented beans. We evaluated nine quality parameters^39–41^ commonly used in the chocolate industry to rapidly assess the commercial quality of fermented cocoa beans (Supplementary Fig. 2j and Supplementary Result 7). We found no differences in the fermentation degree quality parameters across locations, despite Antioquia fermentation signatures being very atypical (Supplementary Fig. 2j). These results suggest that the fermentation markers identified here are not predictive of the overall degree of bean fermentation. Therefore, we postulated that variations in these fermentation signatures, which are associated with chemical processes occurring within the beans, could offer insights into chocolate flavour characteristics.

**Fig. 3:**
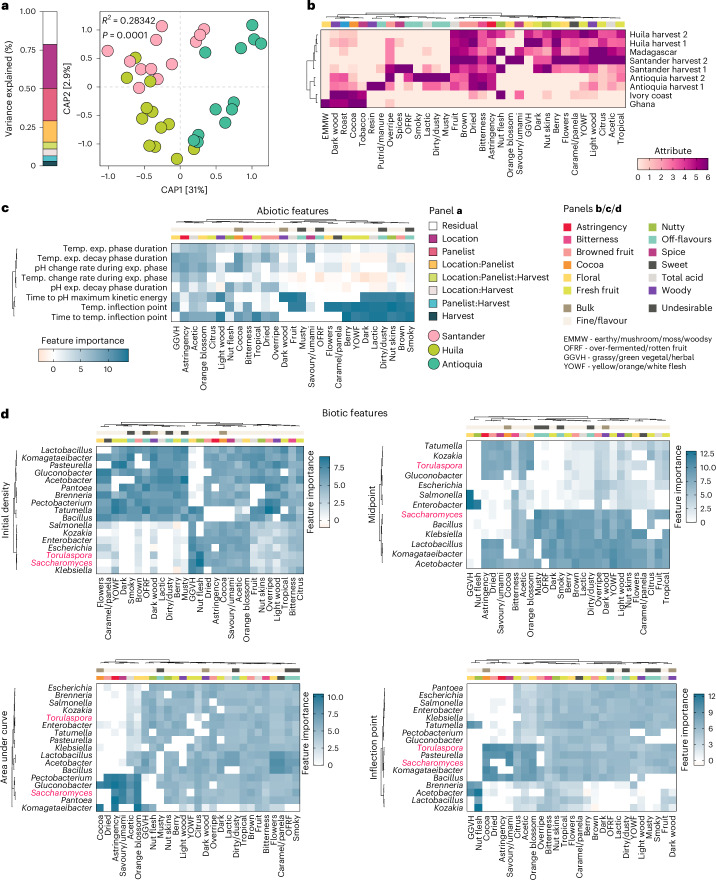
Abiotic and biotic features are linked to sensory attributes of cocoa liquors. **a**, CAP showing the clustering of cocoa liquor sensory profiles from fermented beans produced in Santander, Huila and Antioquia. Group differences were assessed using PERMANOVA with 9,999 permutations. The proportions of sensory attribute variance explained by farm location, harvesting period and sensory panelists, estimated by PERMANOVA, are displayed on the left bar. PERMANOVA *R*^2^ and *P* value explained by farm location are shown. **b**, Heat map illustrating the sensory characteristics of cocoa liquors from the three farms. The bar at the top denotes the sensory groups defined by the Cocoa of Excellence guidelines. Reference liquors from Madagascar (fine cocoa) and Ivory Coast and Ghana (bulk cocoa) were included. **c**, Heat map showing the extracted features of the temperature and pH kinetic curves of fermenting beans from the three farms and their importance to each chocolate sensory attribute. The heat map is clustered hierarchically on the basis of sensory attributes, and selected temperature and pH features, and is coloured on the basis of feature importance determined using the random forest model. The bars at the top denote the sensory groups defined by the Cocoa of Excellence guidelines and highlight attributes typically present in bulk cocoa, fine cocoa and undesirable flavours. **d**, Heat maps showing extracted features from pivotal bacteria and fungi growth curves in fermenting beans and their importance to sensory attributes. Important bacteria and fungi taxa driving differences in beta diversity in cocoa fermentation were selected on the basis of PERMANOVA coefficients. The heat map is coloured on the basis of feature importance, which represents the percentage increase in mean squared error (%IncMSE) for each feature, determined using the random forest model. The heat map is clustered on the basis of hierarchical clustering of the sensory attributes as well as the selected taxa feature. Bacteria are indicated in black text and fungi are illustrated in red text. The bars at the top denote the sensory groups defined by the Cocoa of Excellence guidelines for cocoa liquors and chocolate, and highlight attributes typically present in bulk cocoa, fine or flavour cocoa and undesirable flavours.

To validate this hypothesis, we prepared cocoa liquors from the fermented beans from the three farms and evaluated their flavours using a trained tasting panel. Cocoa beans are classified as ‘bulk’ or ‘fine flavour’^39,41^. Fine flavour beans offer complex, desirable notes with less bitterness and are prized in premium chocolate, whereas bulk beans have a simpler cocoa flavour and are typically more bitter. Consistent with the abiotic and biotic fermentation signature changes (Figs. 1 and 2), liquors from Santander and Huila shared flavour attributes that contrasted with those from Antioquia (Fig. 3a,b and Supplementary Result 8). We observed similar organoleptic qualities between the Santander and Huila liquors and a fine flavour reference liquor from Madagascar (Fig. 3b). In contrast, the Antioquia liquor clustered with bulk references from Ivory Coast and Ghana, showing a narrower flavour range (Fig. 3b). These results underscore the pivotal role of bean fermentation, especially the dynamics of its abiotic and biotic markers defined here, as critical factors driving the development of fine flavour in chocolate.

### Flavour is linked to fermentation abiotic and biotic signatures

**Extended Data Fig. 2:**
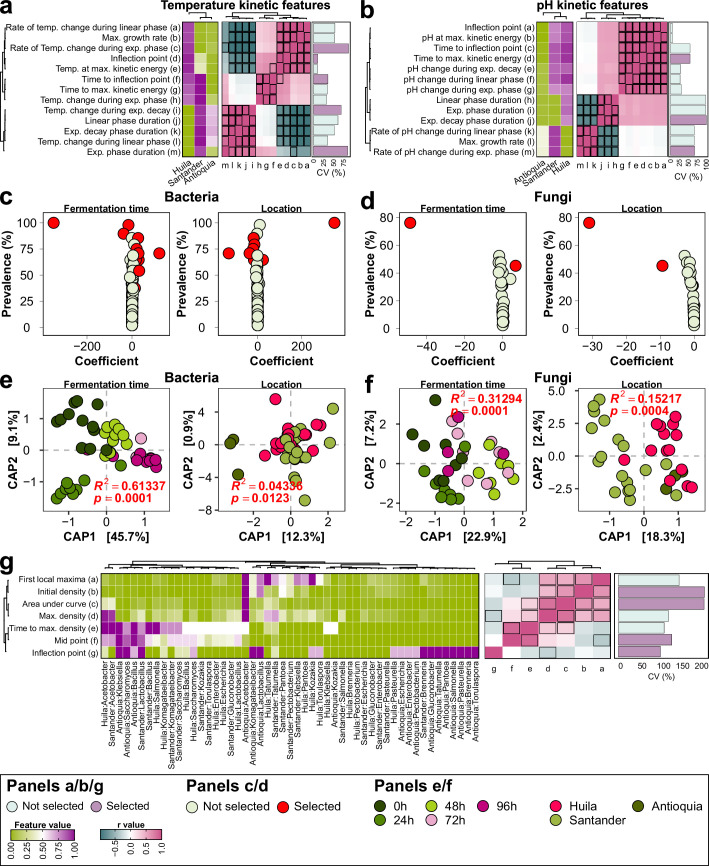
The abiotic and biotic markers extracted from the analysis of the cocoa bean fermentations correlate with desired chocolate attributes. **a**. Features extracted from the temperature kinetic curves and **b**. pH kinetic curves of fermenting beans. The heatmap on the left displays key features extracted from the kinetic curves and is coloured according to mean normalised values. Pearson correlation coefficients for these features are in the centre heatmaps. Relationships were assessed using two-sided Pearson correlation tests. The plot is coloured to reflect the correlation coefficient (*r*) values and significant correlations (*p* < 0.05) are highlighted with black squares. The bar plot on the right indicates the coefficient of variation of the feature extracted, with selected features highlighted in purple. All plots are clustered based on *r* values. **c**. Scatter plots illustrate PERMANOVA coefficients and prevalence of individual bacterial and **d**. fungi taxa across fermentation time points and farm locations in Santander, Huila, and Antioquia. Bacteria and fungi with coefficients ≥ 10 and ≥ 5, respectively, are highlighted in red. **e**. Canonical analysis of principal coordinate of microbiome composition showing the projected bacterial and **f**. fungal community assembly across fermentations. Group differences were assessed using a PERMANOVA with 9999 permutations. PERMANOVA *R*^*2*^ and *p*-values explained by fermentation time points and farm location are indicated within the plots. **g**. Features extracted from the growth curves of selected bacteria and fungi in fermenting beans. Bacteria and fungi growth curves were plotted based on their relative abundance in fermenting beans from Santander, Huila and Antioquia, and growth features of the selected taxa were extracted. The heatmap on the left depicts key features extracted from the growth curves, coloured based on mean normalised values. Pearson correlation coefficients (*r*) for these features are presented in the centre heatmap, with significant correlations (*p* < 0.05) highlighted by black squares. Relationships were assessed using two-sided Pearson correlation tests. The right bar plot indicates the coefficient of variation of the feature values, with selected features highlighted in purple. The three plots are clustered based on *r* values. The left heatmap is also clustered according to taxa abundance.

To assess the predictive value of temperature and cotyledon pH, in predicting chocolate flavour attributes, we fitted their kinetic curves using a 5-parameter model across locations and identified 13 fermentation kinetic features (Extended Data Fig. 2a,b). To reduce redundancy, we clustered pairwise correlations and retained the most variable feature from each cluster for predicting chocolate flavour attributes (Extended Data Fig. 2a,b and Supplementary Result 9). Using these selected features, we identified two main clusters linking abiotic features to flavour notes common to both bulk and fine chocolate (Fig. 3c). Consistent with our previous results (Fig. 3a,b), we observed a strong association between fine chocolate notes, such as light wood, flowers, caramel/panela, brown and dark wood, and abiotic features, such as time to pH maximum kinetic energy, temperature inflection point and time to temperature inflection point (Fig. 3c). This indicates that highly variable features related to temperature and pH strongly predict chocolate flavour characteristics.

We investigated whether microbiota composition changes during fermentation were associated with chocolate flavour attributes. We identified microbial groups driving compositional differences across fermentation time points and locations (Extended Data Fig. 2c,d). CAP analysis using these biotic markers recapitulated both temporal and spatial microbiota variations (Extended Data Fig. 2e,f). Seven growth features, capturing shifts in the microbial marker abundances, were then extracted, and redundancy was reduced using the same clustering approach applied to the abiotic traits (Extended Data Fig. 2g). We observed that changes in the features identified for certain microbes predicted distinct flavour characteristics (Fig. 3d). Notably, changes in the features identified in two fungi genera, *Torulaspora* and *Saccharomyces*, strongly associated with flavour attributes often found in fine chocolate (Fig. 3d).

To further validate the predictive power of the identified markers, we analysed abiotic and microbial feature associations with flavour attributes across 19 and 11 independent natural fermentations, respectively, conducted over multiple years in diverse agroecological regions of Trinidad, a country known for fine flavour cocoa. Despite this broad panel in sensory attributes, strong and consistent associations between several identified markers and key flavour attributes emerged, many matching or exceeding those from Colombian fermentations (Supplementary Fig. 3, Supplementary Table 3 and Supplementary Result 10). These findings indicate that, alongside temperature and pH dynamics, manipulating microbial community composition and dynamics during fermentation can shape chocolate flavour, enabling the development of reproducible starter cultures for consistent, high-quality chocolate production.

### Defined microbes reproduce fermentation metabolic profiles

**Fig. 4:**
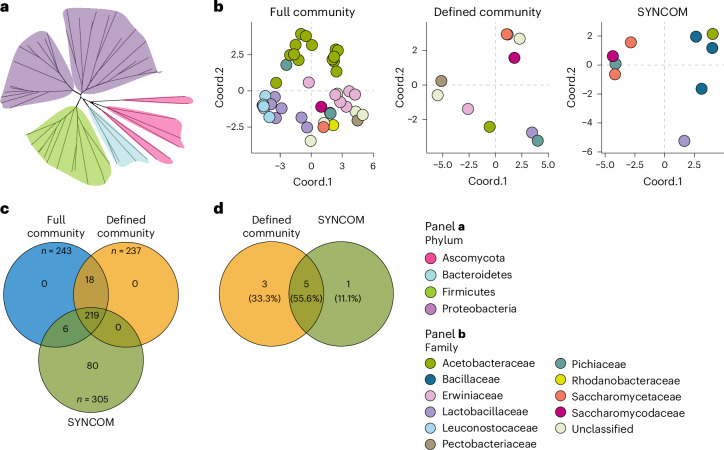
The metabolic characteristics of the microbial communities present in natural cocoa bean fermentations can be condensed into a reduced number of microbes. **a**, Neighbour-joining tree showing the 55 MAGs derived from the fermenting cocoa beans metagenomic dataset. The MAGs were constructed using both single-sample assembly and co-assembly approaches, employing different binning tools. The dendrogram was constructed utilizing marker gene sequences identified across the genomes, with colours indicating the main bacterial and fungal phyla detected. **b**, PCoA showing the projected metabolic potential of the full microbial community (left), defined community identified using our metabolic approach (middle) and the synthetic community (SYNCOM) (right) designed to recapitulate the defined community. Genome-scale metabolic networks were reconstructed from the MAGs and from the genome sequences that were generated from the cultured microbes. Metabolites reachable are based on cocoa pulp as the precursor. The colours depict different bacteria and fungi families. Notice that the defined and the synthetic community cover the main taxonomic units and metabolic groups of the full microbial community. **c**, Venn diagram depicting the number of metabolites attainable by the full, defined and synthetic microbial communities when seeded with cocoa pulp. The total number of metabolites produced by a community (*n*) reflects the cumulative metabolic capabilities of the individual microbes and the added value to produce new metabolites as a result of cooperation among individual microbes in the community. Each circle in the diagram represents the total number of metabolites (*n*) within the community. Overlapping sections denote metabolites shared by multiple communities, while non-overlapping areas signify metabolites exclusive to each community. **d**, Venn diagram illustrating the taxonomic overlap at the family level between the defined community identified in our metabolic network analysis and our synthetic community (SYNCOM). Each circle represents the total number of taxonomic families within a community. The overlapping regions highlight families shared by both communities, while the non-overlapping sections represent families unique to each. The percentages within each region denote the proportion of taxonomic families in that section relative to the total number across both communities.

To guide the design of fermentation starters, we investigated whether a reduced microbial consortium, defined by key taxonomic and metabolic traits, could reproduce the essential characteristics of the cocoa fermentation process. We first constructed metagenome assembled genomes (MAGs) from the shotgun sequencing data of bacteria and fungi associated with fermentation. After removing duplicate MAGs and those that did not meet our genome completeness and contamination thresholds, 55 MAGs remained for further analysis (Fig. 4a, Supplementary Fig. 4a,b, Supplementary Table 4 and Supplementary Result 11). We confirmed that the constructed MAGs reliably captured the microbial community dynamics during fermentation (Supplementary Fig. 4c,d). To assess microbial functional dynamics, we identified enriched Gene Ontology (GO) terms in contigs assembled from the microbial communities that served as the source for MAG reconstruction. Consistent with microbial composition patterns (Supplementary Fig. 4d), functional analysis confirmed similar enrichment dynamics in Santander and Huila fermentations, contrasting sharply with Antioquia. Key enriched GO categories included microbial division, growth and processes typical of food fermentation, such as pH response, alcohol biosynthesis and metabolism, and responses to heat, starvation, osmotic and oxidative stresses^42–44^ (Supplementary Fig. 5, Supplementary Table 5 and Supplementary Result 11). Again, pathway and protein enrichment analyses further highlighted key enzymatic groups enriched during fermentation that probably contribute to cocoa bean flavour development, including aminotransferases (linked to aroma precursors such as pyrazines^45,46^), l-aspartate 4-carboxy-lyase (producing sweet and savoury flavour precursors^47,48^) and ethanolamine ammonia-lyase (generating fruity volatiles such as acetaldehyde^3,9,49^) (Supplementary Fig. 6, and Supplementary Tables 6 and 7). Taken together, these findings suggest that the sequenced microbial communities from the fermentations and the corresponding assembled MAGs potentially capture essential metabolic pathways for cocoa bean fermentation and the development of diverse chocolate flavour attributes. However, due to the incomplete nature of some MAGs, certain metabolic pathways may still be underrepresented.

**Extended Data Fig. 3:**
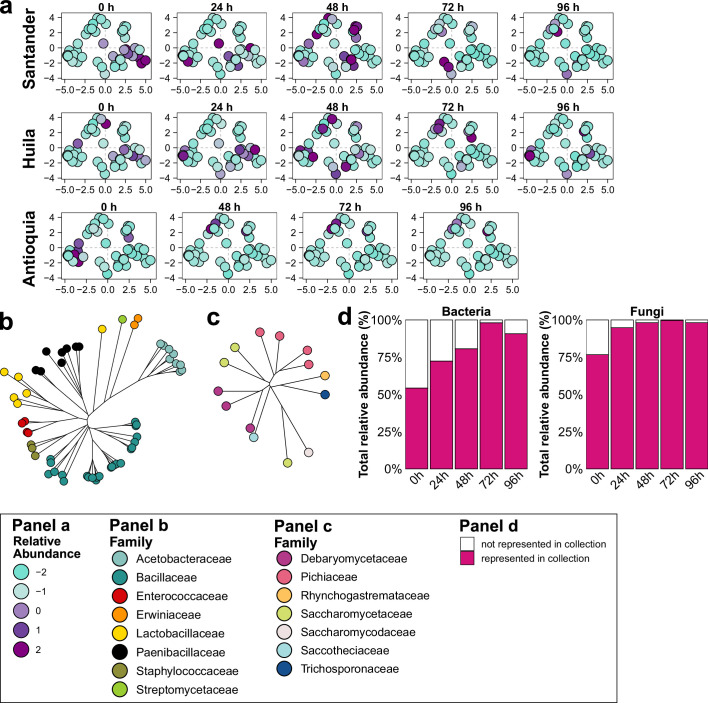
A defined microbial synthetic community reproduced microbiota characteristics of cocoa beans fermentation. **a**. Principal coordinate analysis (PCoA) showing the projected metagenome assembled genomes (MAGs) abundance, with varying metabolic potentials, throughout the fermentation process in Santander, Huila, and Antioquia. The metabolic potential of each MAG, derived from cocoa pulp as the precursor, was transformed into a distance matrix and then converted into two dimensions using classical multidimensional scaling. MAGs are coloured based on their relative abundance during fermentation at each time point for each region. **b**. Neighbour-joining trees displaying bacteria and **c**. fungi isolates cultured in this study. The tips of the trees are coloured according to Phylum. **d**. The bar graph illustrates the total relative abundance of the microbial communities, in fermenting cocoa beans (from Santander, Huila, and Antioquia), our isolates represent at the family level, at different time points during the process.

To evaluate the metabolic capabilities of the MAGs, we reconstructed in silico, genome-scale metabolic networks for 44 MAGs specifically recovered from fermenting beans. Using cocoa pulp substrates^19,27,32,50,51^ to seed the network, we predicted metabolite production for individual MAGs to assess the cumulative metabolic capacities of the MAG set, a representative proxy of the fermentation microbiota (Supplementary Fig. 7). We observed strong taxonomic clustering, with closely related microbes producing similar metabolites (Supplementary Fig. 7). As expected, microbes with similar metabolic capacities followed comparable abundance trajectories in the Santander and Huila fermentations but diverged markedly in the Antioquia fermentation (Extended Data Fig. 3a). These findings indicate that taxonomic and metabolic features essential for cocoa fermentation are redundantly present within the microbial community. Indeed, our metabolic network analysis pinpointed 10 MAGs with metabolic capacities equivalent to the microbial community in cocoa fermentations (Fig. 4b,c and Supplementary Result 12).

**Extended Data Fig. 4:**
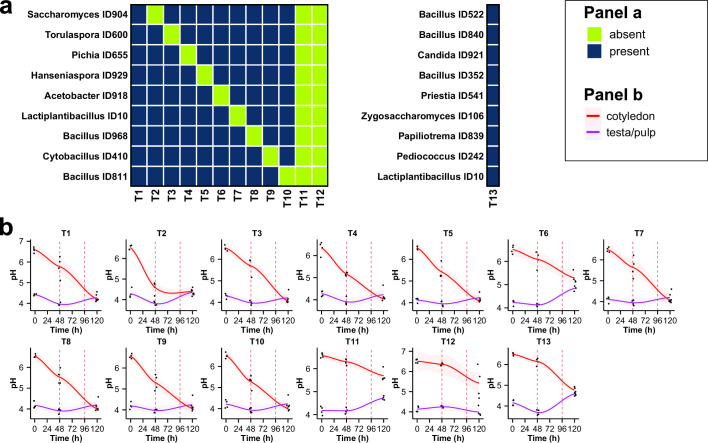
Removal of individual members of the synthetic community did not affect the kinetics of pH changes in the cotyledons and testa/pulp. **a**. Schematic representation of the experimental design showing the combinations of microbes used in cocoa beans fermentations performed using a full synthetic microbial consortium (T1, 9-member SYNCOM) and modified versions where individual strains were removed (T2–T10). Controls included non-inoculated beans (T11 and T12) and beans inoculated with a randomly selected 9-member microbial consortium (T13). **b**. The graphs illustrate the kinetics of pH changes in the cotyledons (red) and testa/pulp (purple) of cocoa beans fermented in vitro using the microbial combinations described in panel a. Smoothed lines indicate fitted trends based on raw data, with shaded areas representing 95% confidence intervals. The red dashed lines indicate the time at which the beans were turned during the fermentation.

To evaluate this reduced consortium, we established a collection of bacterial and fungal strains isolated from fermenting cocoa beans (Supplementary Result 13). This representative collection captured the dominant microbial families involved in Colombian cocoa fermentations (Extended Data Fig. 3b–d and Supplementary Table 8). We selected a subset of 9 strains (5 bacteria, 4 fungi; Supplementary Table 8) that approximated the taxonomy (55.6%) and metabolic potential (94.9%) of the 10 identified MAGs (Fig. 4b–d and Supplementary Result 14). We then tested the consortium in controlled cocoa fermentations using microboxes. We inoculated beans with the full consortium, individual strain dropouts (where individual strains were removed from the consortium), a randomly selected consortium, and included uninoculated controls (Extended Data Fig. 4a).

**Extended Data Fig. 5:**
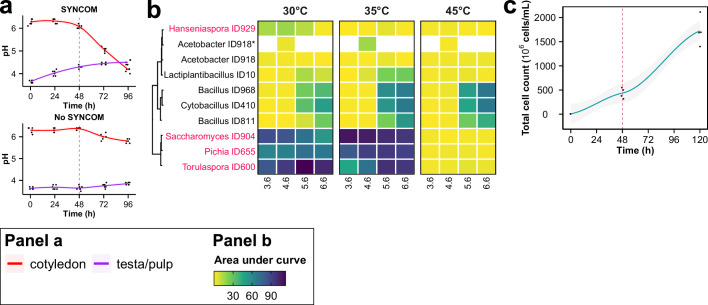
Temperature and pH changes could determine the composition of the microbiota community in fermentations. **a**. The graphs depict the kinetics of pH changes in the cotyledons (red) and testa/pulp (purple) measured in cocoa beans fermented in vitro fermentations inoculated (SYNCOM; top)) or not (No SYNCOM; bottom) with a synthetic microbial consortium at different time points. Smoothed lines indicate fitted trends based on raw data, with shaded areas representing 95% confidence intervals. The red dashed lines indicate the time at which the beans were turned during the fermentation. **b**. The heatmap depicts the growth of individual isolates from the defined synthetic community (SYNCOM). Isolates were cultured in artificial cocoa pulp media under varying pH conditions and temperatures for 72 h. Growth is represented by the area under the growth curve, with the heatmap colour-coded to reflect these values. Additionally, isolate Acetobacter ID918 was also cultured in acetic acid medium, as indicated by an asterisk (*). **c**. Total cell counts were determined in in vitro fermentations inoculated with the defined synthetic community and measured at different time points throughout the fermentation process. Smoothed lines indicate fitted trends, with shaded areas representing 95% confidence intervals.

Inoculated beans with the synthetic microbial communities (full, dropouts and random) generally recapitulated the pH kinetics in the testa/pulp and cotyledons observed during fermentations at Santander and Huila farms, both associated with fine flavour chocolates (Figs. 1a and 2b, Extended Data Figs. 4b and 5a, and Supplementary Result 14). In contrast, these pH shifts were absent in uninoculated beans (Extended Data Figs. 4b and 5a). Further analyses revealed that elevated temperatures and acidic conditions, typical in natural fermentations, significantly impaired the growth of consortium member strains (Extended Data Fig. 5b). These findings suggest that dynamic pH and temperature shifts may drive microbial succession and interkingdom coordination during fermentation.

**Fig. 5:**
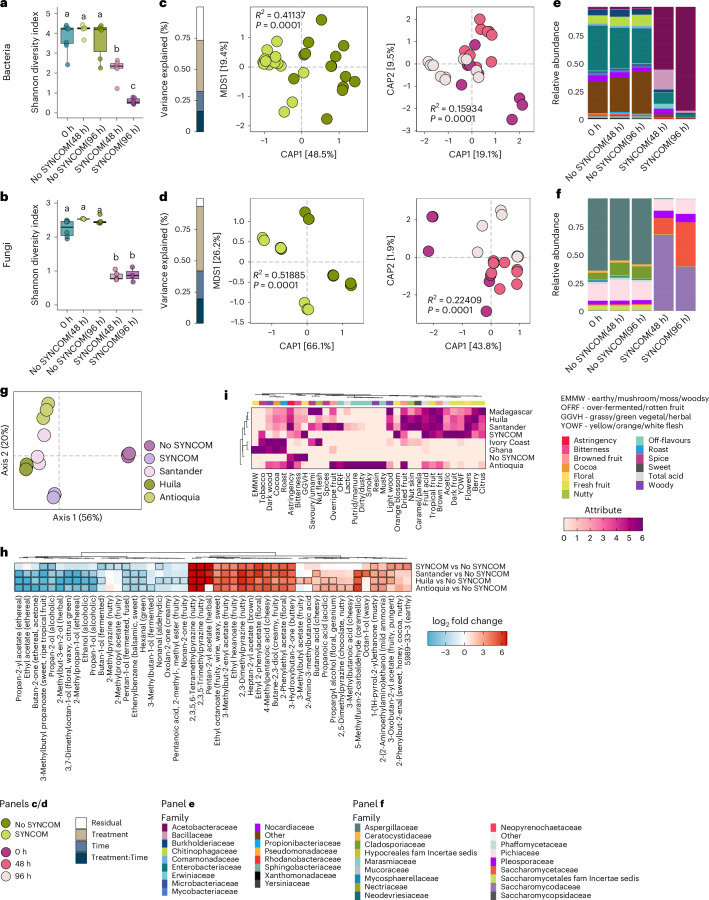
A microbial consortium replicates desirable traits in cocoa fermentation. **a**,**b**, Bacterial (**a**) and fungal (**b**) alpha diversity estimate of in vitro fermented beans inoculated (SYNCOM) or not (No SYNCOM) with a synthetic microbial consortium. Three independent fermentations were conducted and 32 bacterial and 22 fungal community profiles were generated. Alpha diversity was estimated using the Shannon diversity index. The horizontal line within each box indicates the median; box edges represent the interquartile range from the 25th to 75th percentiles; whiskers extend to the smallest and largest values within 1.5× the interquartile range of the lower and upper quartiles; and individual data points, including outliers, are overlaid as dots. ANOVA was used to identify significant differences among groups, and means were separated using Tukey’s post hoc test. Letters indicate similarities and differences between groups (*P* < 0.05). **c**,**d**, Canonical analysis of principal coordinates illustrating bacterial (**c**) and fungal (**d**) beta diversity estimates from in vitro fermented beans. Differences in beta diversity are illustrated for the experimental treatment effect (inoculated and not inoculated; left) and the fermentation time effect (right). Group differences were assessed using PERMANOVA with 9,999 permutations. The PERMANOVA *R*^2^ and corresponding *P* value are shown. The bar on the left denotes the percentage of the variance explained by the experimental variables. **e**,**f**, Phylogram showing the relative abundance profiles of the main bacterial (**e**) and fungal (**f**) families in the in vitro fermentations. **g**, PCA of the volatiles detected in liquors made from beans fermented in vitro in comparison to liquors from Santander, Huila and Antioquia. **h**, Heat maps showing the enrichment of volatiles detected in liquors made from beans fermented in vitro as well as from the three farms. Squares outlined in black are volatiles significantly enriched (red) and depleted (blue) with respect to the No SYNCOM control (*q* < 0.05 and log_2_ fold change > ±2). Volatiles are clustered according to their log_2_ fold change values. **i**, Heat map illustrating the sensory characteristics of liquors made from beans fermented in vitro in comparison to liquors from the three farms. The bar at the top denotes the sensory groups defined by the Cocoa of Excellence guidelines.

**Extended Data Fig. 6:**
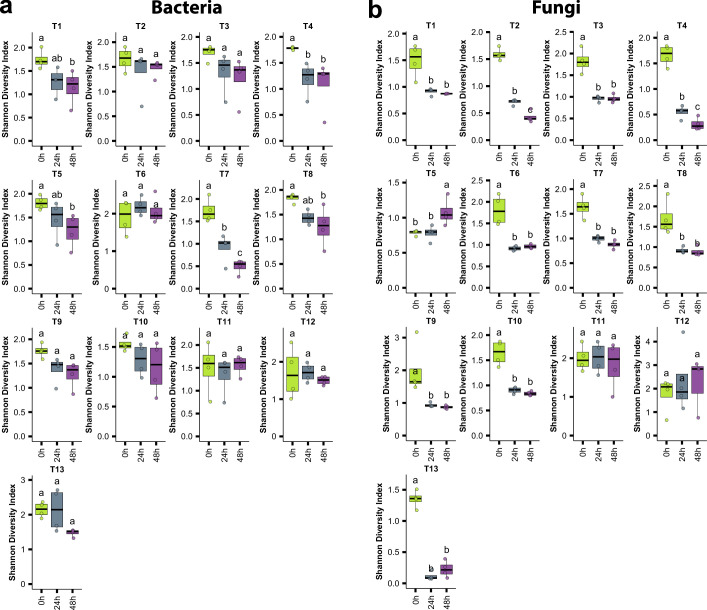
Removal of individual members of the synthetic community affected the composition of the microbial community in the fermentations. **a**. Graphs showing the alpha diversity estimates of bacterial and **b**. fungal communities in fermenting cocoa beans inoculated with the full microbial synthetic community (T1, 9-member SYNCOM) and modified versions where individual strains were removed (T2–T10). Controls included non-inoculated beans (T11 and T12) and beans inoculated with a randomly selected 9-member microbial consortium (T13). For each treatment, four independent fermentations were conducted, with a single microbial DNA sample collected from the fermenting mass at 0, 24, and 48 h in each fermentation. After quality filtering, 155 bacterial (n = 155) and 154 fungal (n = 154) community profiles were retained for downstream analysis. The alpha diversity was estimated using the Shannon Diversity Index. Boxplots depict the distribution of Shannon Diversity Index values at different sample time points. The horizontal line inside each box marks the median, and the box edges correspond to the interquartile range (from the 25th to the 75th percentile). Whiskers extend to the minimum and maximum values within 1.5 times the interquartile range from the lower and upper quartiles. Individual data points, including outliers, are displayed as overlaid dots. One-way ANOVA was used to identify significant differences among groups and means were separated using Tukey’s post hoc test. Letters indicate similarities and differences between groups (*p* < 0.05).

**Extended Data Fig. 7:**
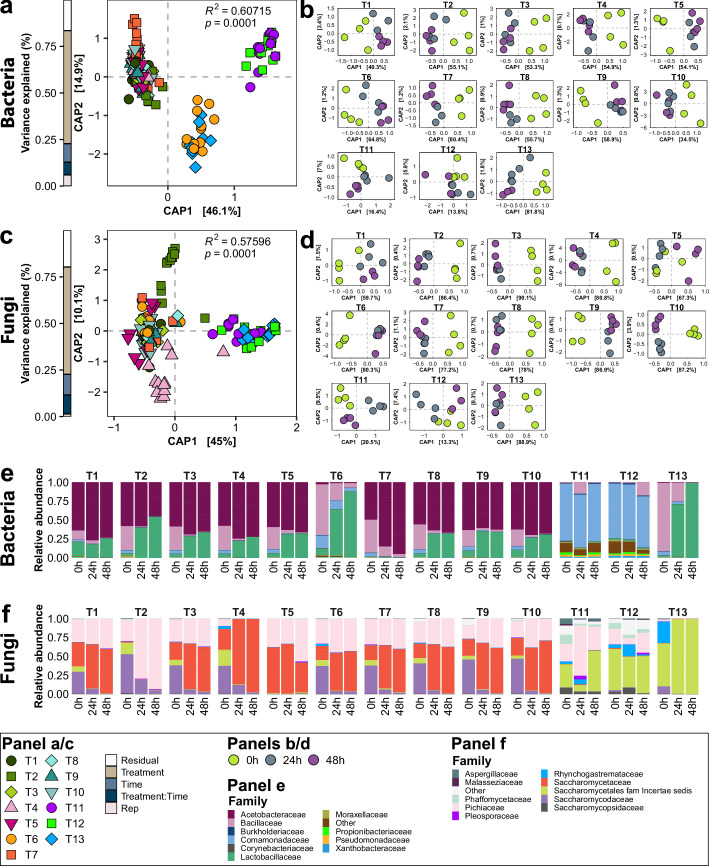
Removal of individual members of the synthetic community influences the composition of the microbial community in the fermentations. **a** Canonical analysis of principal coordinate (CAP) of microbiome composition showing the projected bacterial community assembly across the different in vitro fermentations inoculated with the full microbial synthetic community (T1, full 9-member SYNCOM) and modified versions where individual strains were removed (T2–T10). Controls included non-inoculated beans (T11 and T12) and beans inoculated with a randomly selected 9-member microbial consortium (T13). Group differences were assessed using a PERMANOVA with 9999 permutations. CAP ordination illustrates sample clustering by synthetic community treatment, with the PERMANOVA *R²* and *p*-value displayed within the panel. The bar on the left describes the variance explained by the different experimental variables. **b**. CAPs analysis showing the projected bacterial community assembly in the individual fermentations inoculated with the different microbial combinations described in panel a at distinct time points. **c**. CAP analysis of microbiome composition showing the projected fungal community assembly across the different in vitro fermentations inoculated with the different microbial combinations described in panel a. Group differences were assessed using a PERMANOVA with 9999 permutations. CAP ordination illustrates sample clustering by synthetic community treatment. The PERMANOVA *R²* and *p*-value are shown. The bar on the left describes the variance explained by the different experimental variables. **d**. CAPs analysis showing the projected fungal community assembly in the individual fermentations inoculated with the different microbial combinations described in panel a at distinct time points. **e**. Phylogram illustrating the relative abundance of key bacterial and **f**. fungal families across in vitro fermentations inoculated or not with the different microbial combinations described above at distinct time points. The 0 h time point represents samples collected after the initial synthetic microbial consortium was applied. The proportions in the bar plots represent the average relative abundances of each taxon, calculated across multiple replicate samples for the bacterial (n_total_ = 155) and fungal (n_total_ = 154) communities.

We reproduced key microbiota features of the Santander and Huila fermentations (Figs. 1c–f, 2c–f and 5a–f, and Supplementary Result 14). Microbial cell density increased progressively during the process (Extended Data Fig. 5c). Inoculation with the full synthetic community led to a decline in bacterial and fungal alpha diversity during fermentation, mirroring farm observations (Fig. 5a,b). In dropout experiments, this diversity decline was lost in five bacterial and one fungal dropout combinations (Extended Data Fig. 6a,b). Analysis of community structure revealed that the microbial composition was primarily shaped by the initial starter formulation and fermentation time (Fig. 5c–f, Extended Data Fig. 7 and Supplementary Fig. 8), highlighting the critical role of specific strains for maintaining microbial community structure. Together, these findings demonstrate that defined synthetic starters, under controlled conditions, can replicate the key abiotic and biotic features of spontaneous farm cocoa fermentations.

**Extended Data Fig. 8:**
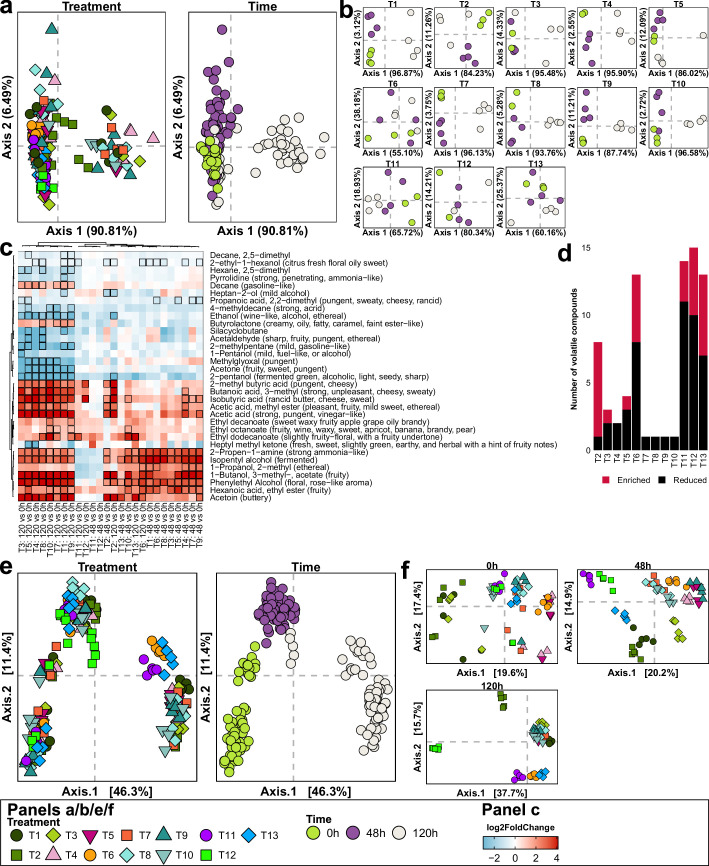
Volatile and non-volatile compounds detected in cocoa beans fermentations change with the fermentation progression and microbial composition of the starters. **a**. Principal coordinate analyses (PCoA) showing the volatile compound profile projections of cocoa beans fermentations driven by different synthetic microbial consortium and **b**. fermentation time. Fermentations were carried out using a full synthetic microbial consortium (T1, 9-member SYNCOM) and modified versions where individual strains were removed (T2–T10). Controls included non-inoculated beans (T11 and T12) and beans inoculated with a randomly selected 9-member microbial consortium (T13). **c**. Heatmaps displaying the enrichment profiles of individual volatile compounds detected in beans fermentations from a. Squares outlined in black indicate volatile compounds that were significantly enriched (red) or depleted (blue) relative to the start of fermentation (0 h) (*q* < 0.05 and log_2_ fold change > ±2). Both volatile compounds and bean samples are clustered based on their log_2_ fold change values. **d**. Bar plot showing the number of enriched and depleted volatile compounds detected in each synthetic microbial consortium treatment compared to T1 at 120 h. **e**. Principal coordinate analyses (PCoA) showing the non-volatile compound profile projections in cocoa beans during fermentation with the different starters (left) described in panel a, and across fermentation time (right). **f**. Principal coordinate analyses (PCoA) showing the non-volatile compound profile projections in cocoa beans during fermentation driven by the starters described in panel a at different fermentation times (0, 48, 120 h). Points are coloured according to the microbial starter treatments.

**Extended Data Fig. 9:**
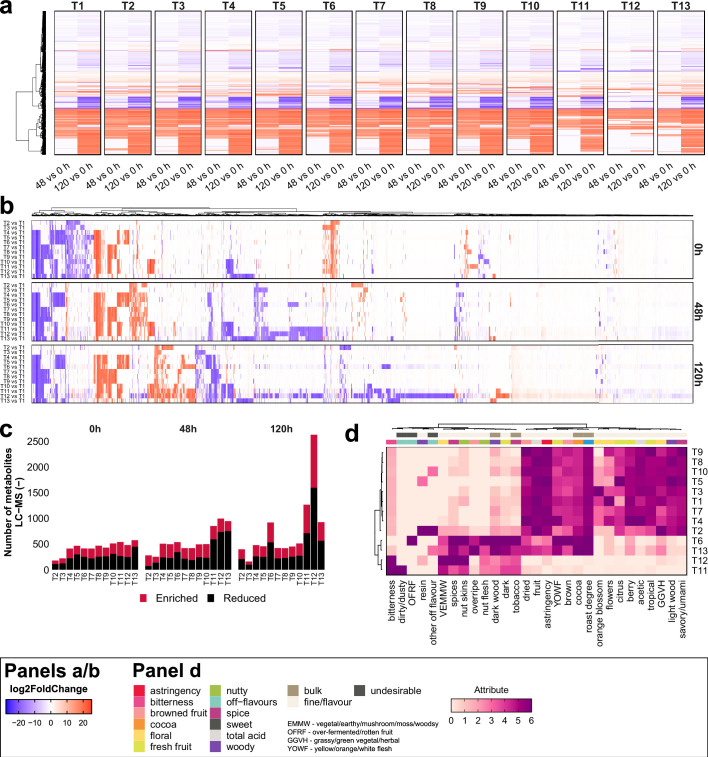
The non-volatile compound profiles of fermenting cocoa beans and the sensory attributes of the resulting liquors change depending on the microbial composition of the starters used in the fermentations. **a**. Heatmaps showing individual non-volatile compounds enriched or depleted at 48 h and 120 h compared to 0 h for each microbial treatment used. Cocoa bean fermentations were conducted using the full synthetic microbial consortium (T1, 9-member SYNCOM) and modified versions where individual strains were systematically removed (T2–T10). Control conditions included non-inoculated beans (T11, T12) and beans inoculated with an alternative, randomly selected 9-member consortium (T13). **b**. Heatmaps showing individual non-volatile compounds enriched or depleted in the different fermentations (T2-T13) described above relative to the fermentation with the full synthetic community (T1; 9-member SYNCOM) at 0 h, 48 h, and 120 h. A generalised linear model was used to assess enrichment, with significant changes determined by an adjusted *p*-value < 0.05 and log₂ fold change > ±2. Heatmaps are colour-coded, with red indicating enriched compounds and blue indicating depleted compounds. Hierarchical clustering highlights trends in compound variation across different time points and fermentation treatments. **c**. Bar plot showing the number of enriched and depleted non-volatile compounds detected in each synthetic microbial consortium treatment compared to T1 at 0 h, 48 h, and 120 h. **d**. Heatmap representing sensory attributes of cocoa liquors produced from beans fermented with the microbial consortia described in panel a. Mean sensory scores from panel evaluations were normalised between 0 and 6. The coloured bar at the top categorises sensory profiles based on the Cocoa of Excellence classification. Clustering was applied to identify similarities in sensory characteristics among different cocoa liquors.

We tested whether the reproducibility of general fermentation characteristics under controlled conditions with synthetic starters extended to metabolic transformations linked to chocolate flavour. We prepared cocoa liquors from beans fermented under controlled conditions and analysed volatile organic compounds (VOCs) associated with chocolate flavour^3,9,46,52–57^. We compared these to cocoa liquors from the on-farm fermentations in Santander, Huila and Antioquia. Principal component analysis revealed a clear separation between the metabolic profiles of inoculated and farm fermentations versus non-inoculated controls (Fig. 5g). VOC enrichment patterns were similar between the controlled and on-farm fermentations, but distinct from non-inoculated controls (Fig. 5h). We confirmed that changes in VOC metabolic profiles were linked to initial starter composition and evolved throughout fermentation (Extended Data Fig. 8a,b and Supplementary Fig. 9a). Kinetic analyses across the full, dropout and random synthetic starters revealed significant differences in VOC concentrations, while VOCs in non-inoculated fermentations remained largely unchanged compared to time zero (Extended Data Fig. 8a–d, Supplementary Fig. 9a and Supplementary Result 15). Non-volatile metabolite profiles also differed across the synthetic communities and diverged further over time (Extended Data Fig. 8e,f and Supplementary Fig. 9b). At later stages, non-inoculated and random-inoculated fermentations were metabolically distinct from others (Extended Data Fig. 8e,f, Supplementary Fig. 9b and Supplementary Result 15). Hundreds to thousands of metabolites showed distinct accumulation kinetics (Extended Data Fig. 9a–c), confirming limited redundancy in the metabolic capacities of the reduced starter (Extended Data Fig. 9b,c). These results highlight the reproducibility and tunability of flavour-associated metabolic profiles using defined synthetic communities.

A trained tasting panel confirmed that liquors from beans fermented with the full synthetic community exhibited flavour notes characteristic of fine chocolates from Santander, Huila and Madagascar, clearly distinguishing them from bulk chocolate references (Fig. 5i). In contrast, dropouts, the random community and non-inoculated fermentations produced liquors with altered or diminished flavour complexity (Extended Data Fig. 9d). These results demonstrate that defined microbial starters can reliably reproduce and modulate fine chocolate traits under controlled conditions.

## Discussion

We establish the previously suggested role of pH and temperature changes^1,58^ as robust predictors of chocolate flavour characteristics. In addition, we characterize microbial dynamics during bean fermentation to reveal interkingdom microbial interactions that promote flavour profile development in cocoa beans. Our study demonstrates that both abiotic and biotic fermentation markers influence the flavour characteristics of chocolate liquors, supporting previous hypotheses^6,58^. We expose the origins of spontaneous microbial assembly driving fermentations on farms, shedding light on flavour attributes of cocoa beans linked to specific geographic locations. Furthermore, by defining the relationship between abiotic and biotic fermentation signatures and flavour notes, our findings provide a foundation for manipulating flavour profiles of cocoa beans. These conclusions have been validated across diverse agroecological cocoa-growing regions, spanning two countries and multiple harvest seasons.

We demonstrate using synthetic community dropout experiments that the metabolic capabilities required to ferment cocoa are redundantly represented within the fermentation microbial community and therefore can be encapsulated in a reduced microbial consortium under controlled conditions. While the dropout experiments, removing only one microbial strain at a time, strongly support a reduced functional redundancy present in the fermentation starter designed in this work, these dropout experiments may not be sufficient to capture higher-order metabolic interactions within the microbial community. Therefore, a fully combinatorial design in the dropout experiments may be required to convincingly demonstrate that the metabolic capabilities contributed by each starter member are unique. Similarly, due to the incomplete nature of some eukaryotic MAGs, certain metabolic pathways may be underrepresented or absent in our analysis. Therefore, we speculate that increasing the quality of eukaryotic MAGs could lead to the design of new starters with specific metabolic capabilities. These new starters are expected to generate novel chocolate flavour profiles through mechanisms similar to those described here.

The results of this work expand our understanding of how the microbial community composition present in fermentation is a key determinant of chocolate flavour characteristics. We developed a robust pipeline enabling the design of fermentation starters that will contribute to the domestication of spontaneous and unpredictable microbial fermentation of cocoa occurring on farms. This sets the stage for the emergence of a modern chocolate industry akin to the beer or cheese industry, based on controlled cocoa fermentations, driven by synthetic microbial starters capable of robustly reproducing unique flavour attributes in cocoa beans and chocolate.

## Methods

### Farms selection

Cocoa plantations used in this work were distributed throughout Colombia and were separated into three agroecological zones on the basis of climatic conditions, topography and soil composition. The selected agroecological zones were: (1) Santander, a mountainous region with the largest production rates of cocoa in the country, (2) Huila, an inter-Andean dry valley region and (3) Antioquia, a Pacific region. For each zone, one farm was selected for sampling: Santander (3,888 ft above sea level), Huila (3,640 ft above sea level) and Antioquia (3,993 ft above sea level) (Extended Data Fig. 1a). The Huila and Antioquia farms were located ~430 km apart, and ~550 km and 198 km away from the analysed farm in Santander, respectively (Extended Data Fig. 1a). For the farms selection, we also considered best agricultural practices, a well-established infrastructure and characteristics of cocoa fermentation. All regions had a tropical and humid climate, and the monthly average temperature during sample collection was from 22.8 °C to 28.7 °C.

### Cocoa bean fermentation in the farms

All cocoa bean fermentations were performed on the farms using the farmers’ traditional practices and were analysed during the mid (May) and main harvests (October–November) on the three farms except for Antioquia, where only the main harvest period was characterized. Briefly, mature ripe cacao pods were harvested and opened manually in the fields. Cocoa beans and surrounding pulp were scooped out by hand and placed into pre-washed wooden fermentation boxes. The beans (200–400 kg) were then covered with banana leaves and/or jute bags to control the environmental temperature in the boxes. Natural cocoa bean fermentation proceeded at ambient temperatures ranging from an average minimum temperature of 22 °C (night-time) to a maximum of 33 °C (daytime). In all cases, the beans were turned at 48 h and 96 h after fermentation began. Fermented beans were removed from the fermentation box 144 h or 168 h after the start of the fermentation on the basis of the temperature, pH and cut test results.

### Temperature, pH and bean colour analyses

To ensure the reproducibility of our observations, we only evaluated fermentation events that followed the traditional practices of local farmers who use wooden boxes to ferment cocoa beans. By maintaining these traditional protocols, we minimized the risk of introducing experimental bias (other types of practices such as sacks, bamboo baskets, plastic baskets, styrofoam and others) that could have altered the natural fermentation trajectory and influenced the final flavour profiles. This approach allowed us to preserve the authenticity of the fermentation process, ensuring that our results reflected the typical outcomes of the region’s traditional methods. Consequently, this consistency helped in achieving reproducibility of the fermentation process and flavour profiles under the experimental conditions. The temperature of the fermentation mass within the boxes was recorded daily using a Brannan digital thermometer (Extended Data Fig. 1b). Measurements were taken at three different zones in the box (bottom left corner, middle and top right corner) at two depths: (1) 7 cm below the top surface of the beans and (2) midway through the fermenting mass. For pH measurement, three beans were collected from the boxes, 7 cm below the top surface in each of the three selected fermentation zones. Testa (seed coats) covered with pulp were separated from the cotyledons of the beans and both tissues (testa/pulp and cotyledons) were macerated in 10 ml distilled water using a mortar and pestle. Then, the pH of the suspensions was determined using a Hanna Checker H198103 pH tester. The colour changes of the beans were determined using images taken daily with a Samsung SM-G9600 camera in automatic mode. RGB values were extracted from the bean images using ImageJ v.1.54d. A minimum of eight points on each bean image were selected for each analysed time point. Greyscale and luminance values were derived using the formulas (R + G + B)/3 and 0.299 R + 0.587 G + 0.114B, respectively. Results from the temperature and pH measurements, along with the colour changes of the beans, were collectively used to determine the end of the fermentation.

### Sample collection for microbial community analyses

Samples for the microbial community analyses were collected from the fermenting beans daily using a Zymo Collection Swab (R1104) (Extended Data Fig. 1b). For the collection, we removed the top 7 cm of beans from the fermenting mass, at the centre of the fermentation box to create a small cavity, and samples were collected in duplicate by swabbing the surfaces of the beans at the bottom of the cavity. The swab buds were then placed in Zymo DNA/RNA Shield Lysis and Collection tubes (R1104), and the tube contents were mixed by vigorous shaking for 10 s. A total of *n* = 66 fermentation samples were collected across the three farms. Using the same protocol, swab samples were also collected from various environmental sources on the farms, including the surface of cacao leaves and pods, the inner surface of the fermentation box, the hands of farm workers involved in scooping beans and transferring them to the fermentation box, and their pod cracking tools. Soil samples were collected by discarding the top 7 cm of soil and transferring 150–250 mg of soil into Zymo DNA/RNA Shield Lysis and Collection tubes using a clean spatula. To analyse the microbiota on fruit flies around the fermentation, fruit flies were caught and incubated in 0.5 ml Zymo DNA/RNA Shield Lysis solution with agitation for 5 min. The flies were then removed, and the solution was transferred into a Zymo DNA/RNA Shield Lysis and Collection tube with an additional 0.5 ml of lysis solution. In total, *n* = 70 farm environment samples were collected for metagenomic analysis across the three farms. Clean swabs were used as control samples without DNA.

### DNA extraction, library preparation and whole-metagenome shotgun sequencing

Samples for microbial community analysis were homogenized using the SuperFastPrep-2 cell lysis homogeniser (MP Biomedicals) at maximum speed for 10 s and were subsequently centrifuged for 1 min and 30 s at 9,800 × *g*. DNA was extracted using the ZymoBIOMICS DNA Miniprep kit (Zymo, D4300) following manufacturer instructions, and the resulting DNA concentration was determined using a Qubit fluorometer (Thermo Fisher). DNA sequencing libraries were prepared using the Rapid PCR Barcoding kit (SQK-RPB004) from Oxford Nanopore Technologies (ONT). In brief, 1 µl fragmentation mix was added to 3 µl DNA (2–10 ng µl^−1^), and the reaction was mixed by gentle finger-flicking. The tube was placed in a miniPCR mini16 thermal cycler (Amplyus) and the DNA was fragmented using the following conditions: 30 °C for 1 min, then 80 °C for 1 min. The fragmented DNA was cooled and amplified in a PCR reaction containing 20 µl nuclease-free water, 25 µl LongAmp *Taq* 2× master mix (New England Biolabs (NEB), M0287L), 4 µl fragmented DNA and 1 µl barcode adaptor. The reaction was gently mixed and amplified using the following conditions: 95 °C for 3 min, 20 cycles of denaturation at 95 °C for 15 s, annealing at 56 °C for 15 s and extension at 65 °C for 6 min, and a final extension of 65 °C for 6 min. The resulting DNA library was purified using 0.6× Agencourt AMPure XP beads (Beckman Coulter, A63881) and eluted in 10 µl 10 mM Tris-HCl pH 8.0 and 50 mM NaCl. The library concentration was determined using a Qubit fluorometer (Thermo Fisher). Equimolar quantities of individual barcoded sample libraries were pooled and the volume adjusted to 10 µl using 10 mM Tris-HCl pH 8.0 and 50 mM NaCl. Subsequently, 1 µl of Rapid Adapter solution was added to the pooled library and the tube was incubated at room temperature for 5 min. Then, 34 µl sequencing buffer, 25.5 µl loading beads and 4.5 µl nuclease-free water were added to the tube, and the contents were mixed gently. The prepared pooled library was added to a verified and primed FLO-MIN106 R9.4.1 flow cell (ONT, FLO-MIN106D) in a MinION DNA sequencer (ONT) following manufacturer instructions. DNA sequencing was conducted with default parameters using MinIT (ONT) with MinKNOW v.2.1.12 (ONT). Fast5 files were base called with Guppy v.4.0.15 using the ‘template_r9.4.1_450bps_hac.jsn’ high-accuracy model (ONT).

### Temperature, pH and bean colour analysis

Temperature and pH distributions were compared using the Kolmogorov–Smirnov test with the ks.test() function from the stats v.4.3.0 package in R and plotted with ggplot2 v.3.4.2. To explore the correlations between temperature and pH in the testa/pulp and cotyledons, scatterplots with correlation coefficients and *P* values were generated using the stat_cor() function from the ggpubr v.0.6.0 package. To assess dissimilarities in bean colour at the different time points during fermentation, principal component analysis (PCA) was performed with the prcomp() function in R, and the first two principal components were plotted using ggplot2 v.3.4.2. In addition, scatterplots with correlation coefficients and *P* values were employed to explore correlations between bean colour values, temperature and pH.

### Processing and filtering of whole-metagenome shotgun sequence data

We obtained a total of 29,714,777 base-called reads (97.7 Gbp) from the whole-metagenome shotgun sequencing across the three farms. The initial dataset underwent demultiplexing, and primer and barcode sequences were trimmed using qcat v.1.1.0 (ONT). Reads with ambiguous barcode assignments were excluded from further analysis. The reads were filtered with NanoFilt (v.2.8.0)^59^ to discard low quality sequences (*Q*-score < 9) and sequences <100 bp. Reads were mapped to the *Theobroma cacao* Criollo v.2.0 reference genome^60^ as well as the *Homo sapiens* reference genome GRCh38.p14 (RefSeq GCF_000001405.40) using minimap2 (v.2.17)^61^ to identify and remove potential contaminating DNA in samples. Reads that mapped were removed using SAMtools (v.1.9)^62^ and Seqtk v.1.3 scripts. Following these processing steps, we retained 24,300,820 reads (80.8 Gbp) with an average read length of 3,326.4 bp (Extended Data Fig. 1h) and a mean read quality of Q13.2.

### Profiling microbiota community composition

We used the Kraken v.2.1.2 pipeline^63^ for classifying the whole-metagenome shotgun sequencing reads. The reads were classified using the Kraken 2 archaea, bacteria, viral, plasmid, human, UniVec_Core, protozoa and fungi reference databases (k2_pluspf_20220607). To estimate relative abundances, the Bracken v.2.7 pipeline^64^ was applied to the classification results. Subsequently, Pavian v.1.0 facilitated the extraction of abundance and taxonomic tables. Functions in phyloseq v.1.44.0 with microbiome v.1.22.0 and microbiomeutilities v.1.0.17 were used to filter the dataset and remove samples with low read depth, remove unidentified taxa and singletons, transform abundance values using rarefaction, subset and merge sample and taxonomic groups, and perform other dataframe manipulations. To assess alpha diversity across the samples, we calculated the Shannon diversity index using phyloseq v.1.44.0. We used analysis of variance (ANOVA) to test for significant differences in Shannon diversity indices between groups, and means were separated using Tukey’s honestly significant difference (HSD) test from the agricolae v.1.3.5 R package. For beta diversity, Bray–Curtis dissimilarity matrices were calculated using the phyloseq v.1.44.0 ‘bray’ method, and the variances explained by fermentation time, farm location and harvest season were estimated by performing permutational multivariate analysis of variance (PERMANOVA) using the adonis2() function in the vegan v.2.6.4 R package. Unconstrained and constrained ordination of beta diversity was plotted using principal coordinate analysis (PCoA) and canonical analysis of principal coordinates (CAP), respectively, on the basis of Bray–Curtis dissimilarity matrices calculated with vegan v.2.6.4. We visualized differences in fermentation time, farm location and harvest season with the CAP analysis, using the following models:

1 $$\sim {\rm{time}}+{\rm{condition}}({\rm{location}}+{\rm{harvest}}+{\rm{replicate}})$$

2 $$\sim {\rm{location}}+{\rm{condition}}({\rm{time}}+{\rm{harvest}}+{\rm{replicate}})$$

3 $$\sim {\rm{harvest}}+{\rm{condition}}({\rm{time}}+{\rm{location}}+{\rm{replicate}})$$

The relative abundance of taxa was plotted as a stacked bar representation using phyloseq v.1.44.0. The tax_glom() function in phyloseq v.1.44.0 was used to agglomerate taxa, and the aggregate_rare() function in microbiome v.1.22.0 was used to aggregate rare groups. Mantel correlations between bacterial and fungal Bray–Curtis dissimilarity matrices were performed using the mantel() function of vegan v.2.6.4, with the Pearson method and 10,000 permutations. We used DESeq2 (v.1.40.0)^65^ to calculate the enrichment profiles at Santander by fitting a generalized linear model (GLM) with the following design:

4 $${\rm{abundance}} \sim {\rm{fermentation\; time}}+{\rm{replicate}}$$

We extracted the following comparisons from the fitted model: 24 h vs 0 h, 48 h vs 0 h, 72 h vs 0 h and 96 h vs 0 h. Taxa at the family, genus and species levels were considered significant if they had a false discovery rate (FDR)-adjusted *P* value (*q* value) < 0.05. The results of the GLM analysis were rendered in heat maps coloured on the basis of the log_2_ fold change output by the GLM. Significant differences between comparisons with a *q* value < 0.05 with log_2_ fold change > ±2 were highlighted with black squares.

### Single-nucleotide polymorphism (SNP) genotyping of cacao varieties

To elucidate the genetic backgrounds of the cacao varieties cultivated across the three cocoa farms, we first conducted a survey focusing on diverse fruit morphologies to gauge the diversity present. The parameters assessed encompassed fruit characteristics such as form, basal constriction, apex, rugosity, ridging, length, diameter, wall thickness and the colour of the mature unripe fruit. Following this morphological survey, young and healthy leaf samples were collected from each distinct morphotype identified (*n*_total_ = 24; Santander *n* = 12, Huila *n* = 5 and Antioquia *n* = 7). The leaf samples were washed and dried, and DNA isolation was carried out using a ZR Plant/Seed DNA MiniPrep kit (Zymo, D6020) with modifications detailed in ref. ^66^. The samples were genotyped at 96 SNP sites (Supplementary Table 1) on a Fluidigm Juno System using a Juno 96.96 Genotyping IFC (Standard BioTools) in accordance with manufacturer instructions. The SNP sites analysed were selected from the *Theobroma cacao* global reference SNP panel^67,68^. Briefly, genotyping assays were prepared using the Juno SNP Type Genotyping Reagent kit (Standard BioTools, 100-8364) and specific SNP type genotyping assays manufactured by Standard BioTools. The Juno 96.96 thermal cycling protocol included a multiplex specific target amplification (STA) step before the SNP genotyping to enrich the template molecules. STA thermal cycling conditions comprised 95 °C for 2 min, followed by 14 cycles at 95 °C for 15 s, and 60 °C for 4 min. For SNP genotyping, reactions were initiated at 95 °C for 10 min, followed by 4 cycles at 95 °C for 15 s, 64–61 °C (1 °C decrease with each cycle) for 45 s, and 72 °C for 15 s. This was followed by 39 cycles at 95 °C for 15 s, 60 °C for 45 s, and 72 °C for 15 s. Fluorescence intensity was quantified using the Fluidigm EP1 software (Standard BioTools), and genotypic calls were automatically made using Fluidigm SNP Genotyping Analysis software v.4.1.3 (Standard BioTools). SNP data generated are available in Supplementary Table 2.

### Drying, roasting and sensory evaluation of liquor samples

The fermented beans were spread on a wooden surface in a 3–4-cm layer for sun drying. The drying mass was mixed every 1.5 h for the first 3 days of drying and every 3 h from the fourth day onward. Drying was carried out carefully to ensure that off-flavours did not develop. The beans were covered overnight and during rainy conditions. All batches underwent drying until reaching a final moisture content of 7%, taking ~7–8 days, after which they were stored in jute bags. Quality assessments were conducted on 100-g samples from each bean lot using cut tests following the procedure outlined in ref. ^41^. The evaluation included identifying characteristics such as underfermented (purple/violet), overfermented (grey/slaty), insect-damaged, chopped/broken, germinated, mouldy, double, or flat bean defects. Beans categorized as fully fermented with no defects and those that were partially purple were considered desirable/high quality. For the roasting process, the beans were placed on shallow perforated trays and roasted at 140 °C for 25 min a convection oven (Sheldon Manufacturing). Subsequently, the beans were cooled to ambient temperature, broken and winnowed to produce cocoa nibs. The nibs were transformed into cocoa liquor on a granite-wheeled melangeur (CocoaTown), reducing the particle size to 16–18 μm. Liquor samples (60 °C) were evaluated through coded, randomized tastings by 3–6 trained sensory panelists in duplicate or triplicate. The panelists consisted of members of the Food Technology, Quality and Sensory Evaluation team at the Cocoa Research Centre, Trinidad. The panel members were trained in accordance with the Cocoa of Excellence Programme guidelines^69^ under the supervision of the panel leader, and are experienced in cocoa sensory evaluation. Flavour descriptors assessed by the panel members were based on the cocoa liquor sensory evaluation template of E.S. Seguine and D.A. Sukha^70^ and expressed as numerical values between 0 and 10. Reference liquors from Madagascar (fine or flavour cocoa) and Ivory Coast and Ghana (bulk cocoa) were included in the sensory analysis. To neutralize palates between tastings, soda crackers and mouth rinsing with still water were employed.

### Analysis of cacao genotypes

SNP genotypic data were generated at 96 SNP sites for each morphotype in the study (*n*_total_ = 24; Santander *n* = 12, Huila *n* = 5 and Antioquia *n* = 7). This dataset was then combined with the SNP profiles from 228 cacao reference accessions sourced from the Cocoa Research Centre, Trinidad, SNP database. The reference SNP profiles were primarily generated from cacao accessions at the International Cocoa Genebank Trinidad and were selected across the 10 cacao genetic clusters identified in ref. ^71^ (*n*_total_ = 228; Amelonado *n* = 28, Contamana *n* = 15, Criollo *n* = 15, Curaray *n* = 22, Guiana *n* = 24, Iquitos *n* = 22, Marañon *n* = 27, Nacional *n* = 17, Nanay *n* = 23 and Purús *n* = 5). In addition, 30 Amelonado-Criollo hybrid accessions were included. The combined dataset was filtered by removing SNPs with >10% missing data and monomorphic SNPs. The result was a final dataset of 84 high-quality SNP markers, with a missing data range between 0 and 3.57% and a mean of 0.52% across all accessions. For phylogenetic analysis, SNP profiles were converted into DNA strings, aligned using DECIPHER v.2.24.0 and transformed into a distance matrix with seqinr v.4.2.16. A neighbour-joining tree was constructed with ape v.5.6.2, and the resulting tree was visualized using ggtree v.3.8.0 with ggtreeExtra v.1.10.0. The genetic distances were further analysed through a PCoA for visualization. The PCoA involved converting genetic distances to 2 dimensions using classical multidimensional scaling with the stats v.4.3.0 package, and plotting with ggplot2 v.3.4.2. Ancestry was inferred using STRUCTURE (v.2.3.4)^72^, employing structure-threader (v.1.3.10)^73^ for parallelized runs across multiple CPU cores. To facilitate the analysis, reference accessions were replicated to ensure a minimum representation of 60 individuals for each of the 10 cacao genetic groups. Simulations were calculated using the admixture model with alpha inferred and independent allele frequency with 200,000 burn-ins and 500,000 Monte Carlo Markov Chain repetitions without any previous genetic or geographic origin information. The number of clusters (*K*) was set from 8 to 12 with 30 iterations for each *K* value. CLUMPAK (Cluster Markov Packager Across *K*) (v.1.1)^74^ was utilized to assess the congruence among independent STRUCTURE runs for each *K* value, and the optimum *K* value was determined according to ref. ^75^.

### Community-wide microbial source tracking analysis

To explore how the surrounding microbial environmental sources in the cocoa plantations may be contributing to cocoa fermentation communities, we used FEAST v.0.1.0 to perform community-wide microbial source tracking analysis. The results were plotted with ggplot2 v.3.4.2.

### Statistical analysis of bean quality and cocoa liquor sensory profiles

Bean quality assessments were analysed using Fisher’s exact test with the fisher.test() function of the stats v.4.3.0 package. For the analysis of sensory attributes in the cocoa liquors, we estimated the variances explained by farm location, harvesting period and sensory panelists by performing PERMANOVA using distance matrices with the adonis2() function in the vegan v.2.6.4 R package. This analysis allowed us to examine how location, harvest and panelist, and their interactions, contributed to the variation in the sensory data (Fig. 3a). A constrained ordination of the sensory attributes was plotted using CAP with vegan v.2.6.4 using the following model:

5 $$\sim {\rm{location}}+{\rm{condition}}({\rm{harvest}}+{\rm{panelist}})$$

To illustrate the sensory characteristics of individual cocoa liquors, the mean scores for each sensory attribute were calculated across panelists for each liquor sample. These scores were then transformed to a scale between 0 and 6 using the rescale() function of the scales v.1.2.1 R package. The transformed scores were visualized on a heat map generated with ggplot2 v.3.4.2. Hierarchical clustering of sensory attributes was applied using the ward.D2 or single method within the hclust() function in R. The clustering was based on Euclidean distances calculated using the dist() function on the transformed scores.

### Extracting abiotic kinetic features and random forest analysis

To identify abiotic features associated with the sensory attributes of the cocoa liquors, we utilized the Practical Program for Forces Modeling (PPFM 2020)^76^ tool to model the kinetics of the temperature changes during bean fermentation across the three locations. This involved randomly selecting a minimum of 15 temperature versus time data points and fitting the temperature curve using a 5-parameter general model equation. From the model, we derived several key features including: (1) maximum growth rate (peak rate at which the system grows during a specified period); (2) time to maximum kinetic energy (duration for the system to reach its maximum kinetic energy level); (3) temperature at maximum kinetic energy (the specific temperature value at the point of maximum kinetic energy); (4) exponential phase duration (period of rapid increase in numbers or activity); (5) linear phase duration (phase where the kinetics rate becomes relatively constant); (6) exponential decay phase duration (timeframe when the system starts to decline after reaching its maximum kinetics); (7) temperature change during exponential phase (change in temperature during rapid exponential kinetics); (8) temperature change during linear phase (alterations in temperature during stable kinetics); (9) temperature change during exponential decay (variations in temperature during the decline following exponential kinetics); (10) rate of temperature change during the exponential phase (speed at which temperature changes during rapid exponential kinetics); (11) rate of temperature change during the linear phase (speed at which temperature changes during stable kinetics); (12) time to inflection point (duration for the system to reach the inflection point, indicating a shift in kinetics pattern); and (13) inflection point (point on the kinetics curve where the curvature changes, signifying a transition in kinetics rate or pattern). The entire process was repeated at least three times for each farm’s fermentation. In addition, we extended our analysis to model the kinetics of the inverse cotyledon pH, extracting similar curve features. The feature values were normalized using the rescale() function in the scales v.1.2.1 R package. The mean normalized feature values were then visually represented on a heat map using ggplot2 v.3.4.2. Subsequently, Pearson correlation coefficients and corresponding *P* values between these features were computed using the rcorr() function in the Hmisc v.5.0.1 package. The results of the correlation analysis were graphically presented using ggplot2 v.3.4.2, where the colour of the plots reflected the correlation coefficient values. Significant correlations (*P* < 0.05) were emphasized with black squares on the plots. Furthermore, the coefficient of variation for the feature values was calculated and depicted using ggplot2 v.3.4.2. The 3 plots were integrated on the basis of the hierarchical clustering of the Pearson correlation coefficients of the features. The clustering employed the ward.D2 method within the hclust() function in R, utilizing Euclidean distances calculated using the dist() function. For each cluster identified, we selected the feature with the highest coefficient of variation as a representative for the cluster. Following this, for each sensory attribute, we employed the randomForest v.4.7.1.1 R package to construct a random forest model. This was done to pinpoint the most significant features associated with each sensory attribute. Subsequently, we visualized the percentage increase in mean squared error (%IncMSE) for each feature by generating a heat map using ggplot2 v.3.4.2. Hierarchical clustering of the feature importance was applied using the ward.D2 method within the hclust() function in R. The clustering was based on Euclidean distances calculated using the dist() function.

### Extracting taxonomic kinetic features and random forest analysis

To identify taxonomic markers associated with the sensory attributes of the cocoa liquors, we began by pinpointing the pivotal bacteria and fungi responsible for the notable variances in beta diversity, specifically focusing on those exerting the most influence on beta diversity disparities observed among fermentation time points and across the three farm locations. To identify the top bacteria and fungi driving the differences in beta diversity across fermentation time and farm location, we calculated PERMANOVA coefficients of the taxa using the adonis() function in the vegan v.2.6.4 R package at the genus level, and assessed their prevalence with microbiome v.1.22.0. The results were visualized using ggplot2 v.3.4.2. Bacteria with coefficients ≥10 and fungi with coefficients ≥5 were selected for further analysis. We verified that the selected bacteria and fungi could recapitulate the differences in the beta diversity of the entire community by performing a PERMANOVA with the selected taxa, as well as a CAP, on the basis of Bray–Curtis dissimilarity matrices calculated with vegan v.2.6.4 using the following models:

6 $$\begin{array}{l}\sim{\rm{fermentation}\;\rm{time}}+{\rm{condition}}\left({\rm{farm}\;\rm{location}}\right.\\\left.+\,{\rm{harvest}\;\rm{period}}+{\rm{replicate}}\right)\end{array}$$

7 $$\begin{array}{l}\sim{\rm{farm}\;{\rm{location}}}+{\rm{condition}}\left({\rm{fermentation}}\;{\rm{time}}\right.\\+\left.{\rm{harvest}}\;{\rm{period}}+{\rm{replicate}}\right)\end{array}$$

Subsequently, the relative abundances of the selected bacteria and fungi were extracted from the metagenomic dataset for each fermentation across the three farms and different fermentation time points. Following this, we utilized the gcplyr v.1.5.2 R package to extract growth features of the selected taxa. The extracted features encompassed: (1) first local maxima (the initial peak density achieved during growth before a subsequent decline); (2) initial density (starting density of taxa, corresponding to the first local minima in taxa density); (3) area under the curve (overall taxa growth); (4) maximum density (peak taxa density, offering insights into the taxa carrying capacity within a specific environment, or alternatively, measures of taxa growth yield or efficiency); (5) time to maximum density (duration taken to reach the maximum taxa density in the environment); (6) midpoint (the moment when the density initially reaches half of the maximum density); and (7) inflection point (the instance when the derivative of the growth curve attains its maximum value). The feature values were normalized using the rescale() function in the scales v.1.2.1 R package. The mean normalized feature values were then visually represented on a heat map using ggplot2 v.3.4.2. Hierarchical clustering of the taxa was performed using the hclust() function with the ward.D2 method, on the basis of Euclidean distances calculated with the dist() function. Subsequently, Pearson correlation coefficients and corresponding *P* values between these features were computed using the rcorr() function in the Hmisc v.5.0.1 package. The results of the correlation analysis were graphically presented using ggplot2 v.3.4.2, where the colour of the plots reflected the correlation coefficient values. Significant correlations (*P* < 0.05) were emphasized with black squares on the plots. Furthermore, the coefficients of variation for the feature values were calculated and depicted using ggplot2 v.3.4.2. The 3 plots were integrated on the basis of the hierarchical clustering of the Pearson correlation coefficients of the features. The clustering employed the ward.D2 method within the hclust() function in R, utilizing Euclidean distances calculated using the dist() function. For each cluster identified, we selected the feature with the highest coefficient of variation as a representative for the cluster. Following this, for each sensory attribute, we employed the randomForest v.4.7.1.1 R package to construct a random forest model. This was done to pinpoint the most significant features associated with each sensory attribute. Subsequently, we visualized the percentage increase in mean squared error (%IncMSE) for each feature by generating a heat map using ggplot2 v.3.4.2. Hierarchical clustering of the feature importance was applied using the ward.D2 method based on Euclidean distances.

### Validation of abiotic and taxonomic markers linked to sensory attributes in cocoa liquors

To assess the robustness of the association between abiotic and taxonomic markers and sensory attributes in cocoa liquors, we modelled the kinetics of temperature and pH changes during bean fermentation across 19 independent fermentations, conducted on cocoa farms in diverse agroecological regions of Trinidad between 2018 and 2022. The raw data, including bean temperature, pH and corresponding bean flavour profiles from fermentations, was obtained from the internal database of the Cocoa Research Centre, The University of the West Indies. Farms and fermentation events were selected to capture the full spectrum of cocoa flavour profiles found in Trinidad, a country renowned for producing high-quality fine or flavour cocoa beans. From the kinetic curves, we extracted the following features: temperature inflection point, time to temperature inflection point, duration of the temperature exponential phase, duration of the temperature exponential decay phase, rate of temperature change during the exponential phase, pH exponential decay phase duration, and pH change rate during the exponential phase. In addition, the relative abundances of the selected bacterial and fungal taxa were extracted from metagenomic data of 11 fermentations. Subsequently, growth curves were plotted as described earlier, and the following growth features were extracted: area under the curve, inflection point, initial density and midpoint. As before, we constructed random forest models to identify the most important abiotic and taxonomic features associated with each sensory attribute. Feature importance was visualized using heat maps, displaying the percentage increase in mean squared error (%IncMSE).

### Construction of metagenome assembled genomes

Various strategies were utilized to construct the MAGs. Initially, a single-sample assembly and binning approach was adopted, where reads from individual samples were assembled into contigs using metaFlye^77^ in the Flye v.2.9 package with default mode. Reads from each sample were subsequently mapped to the respective assembly using minimap2 (v.2.17)^61^, and the corresponding abundance files were generated using SAMtools (v.1.12)^62^. The abundance files were used for metagenomic binning of the contigs using two different binning tools: MaxBin (v.2.2.4)^78^ with default parameters and MetaBAT (v.2.15)^79^ with specific parameters (percentIdentity=85, minContigLength=1000, minContigDepth=1). To help capture low-abundance microbes, a co-assembly and binning approach was implemented. This involved pooling reads from fermentation samples within each region (Santander, Huila, Antioquia), assembling contigs and generating metagenomic bins. In addition, a concatenation of reads from fermentation samples across all three farms was performed, followed by contig assembly and binning. These diverse strategies resulted in the construction of 1,591 MAGs. Subsequently, MAGs underwent dereplication using dRep (v.3.4.0)^80^, with genome filtering options set at 10,000 bp minimum length, 10% minimum completeness, 10% maximum contamination and 95% average nucleotide identity (ANI) threshold for species-level dereplication (see ref. ^81^ for species-level definition). The dereplicated MAGs were evaluated using CheckM (v.1.1.6)^82^ to determine their genome completeness and contamination levels. MAGs were assigned to be either low, medium or high quality based on the completeness and contamination levels recommended by ref. ^83^ (low-quality: completeness 0–50%, contamination <10%; medium-quality: completeness 50–90%, contamination <10%; high-quality: completeness >90%, contamination <5%). After excluding MAGs with contamination levels >10% and those with completeness <50% for bacteria or 30% for fungi, 55 MAGs were retained for further analysis. The completeness and contamination statistics for the final MAG set were visualized using ggplot2 v.3.4.2 (Supplementary Fig. 4a,b), and additional quality and genome statistics can be found in Supplementary Table 4. To assess how well the MAGs represented the fermentation and farm environment communities, sequence reads were mapped to the MAGs using minimap2 (v.2.17)^61^, and mapped reads were identified with SAMtools (v.1.9)^62^ and Seqtk v.1.3 (Supplementary Fig. 4c).

### MAG classification, annotation and abundance

The taxonomic classification of the MAGs was performed using the CAT v.8.22 taxonomic classification pipeline^84^. This process entailed identifying open reading frames (ORFs) within each contig, followed by mapping the predicted ORFs against the NCBI NR protein database. The taxonomic assignment of the MAGs was determined on the basis of the consensus classification of individual ORFs. Taxonomic classification of the MAGs can be found in Supplementary Table 4. To visualize the relationships among the MAGs, a dendrogram was constructed using the neighbour-joining approach, utilizing marker gene sequences identified across the genomes of the 55 MAGs from the CheckM (v.1.1.6)^82^ tool. The marker gene sequences for each MAG were initially merged into a string and aligned using Clustal W in the msa v.1.32.0 package. The alignment was trimmed with microseq v.2.1.6, transformed into a distance matrix with seqinr v.4.2.16, and a neighbour-joining tree was constructed with ape v.5.6.2. The resulting tree was visualized using ggtree v.3.8.0 with ggtreeExtra v.1.10.0 (Fig. 4a). The ribosomal RNA (*rRNA*) genes in the MAGs were identified using Barrnap v.0.9 (https://github.com/tseemann/barrnap). Open reading frames from each MAG were predicted using FragGeneScanRs (v.1.1.0)^85^ with default settings. Functional annotation of predicted proteins was performed using eggNOG-mapper (v.2.1.9)^86^ with the eggNOG v.5.0.2 database^87^ with Diamond (v.2.0.11)^88^ and MMseqs2 release 12-113e3 (ref. ^89^). To assess the relative abundance of the MAGs during fermentations, reads from each fermentation sample across the three regions were mapped to the MAGs using minimap2 (v.2.17)^61^, and CoverM (v.0.6.1)^90^ was used to extract the relative abundance counts with the ‘genome’ mode and transcripts per million coverage method. Subsequently, DESeq2 (v.1.40.0)^65^ was used to determine the enrichment profiles of the MAGs in the three farms by fitting a GLM with the design:

8 $${\rm{abundance}} \sim {\rm{fermentation\; time}}+{\rm{location}}+{\rm{replicate}}$$

We extracted the following comparisons from the fitted model: 48 h vs 0 h, 72 h vs 0 h and 96 h vs 0 h. Significance was determined with an FDR-adjusted *P* value (*q* value) < 0.05. The results of the GLM analysis were rendered in a heat map coloured on the basis of the log_2_ fold change. Significant differences between comparisons (*q* value < 0.05) with log_2_ fold change > ±2 were highlighted with black squares (Supplementary Fig. 4d).

### Enrichment of microbial biological functions during fermentation

To identify metabolic processes that were enriched within the microbial communities during the fermentation, we selected the contigs assembled from individual samples (380,365 contigs) and subsequently subjected them to deduplication using the dedupe.sh tool in BBTools v.38.76 to eliminate redundancies. Next, we determined the relative abundance of the contigs during fermentations by mapping the reads from the samples to the contigs using minimap2 (v.2.17)^61^, and extracting the relative abundance counts using CoverM (v.0.6.1)^90^ in the ‘contig’ mode and reads_per_base coverage method. Taxonomic classification of the contigs was performed using the CAT v.8.22 taxonomic classification pipeline^84^. Subsequently, the contigs were filtered to retain only bacterial and fungal sequences. DESeq2 (v.1.40.0)^65^ was utilized to determine the contig enrichment profiles in the three farms by fitting a GLM with the following design:

9 $${\rm{abundance}} \sim {\rm{fermentation\; time}}+{\rm{location}}+{\rm{replicate}}$$

We extracted the following comparisons from the fitted model: 48 h vs 0 h, 72 h vs 0 h and 96 h vs 0 h. Contigs meeting the criteria of an FDR-adjusted *P* value (*q* value) < 0.05 and a log_2_(fold change) > ±2 were selected for further analysis. Open reading frames encoded within the contigs were predicted using FragGeneScanRs (v.1.1.0)^85^ with default settings. This was followed by functional annotation of the predicted proteins using the eggNOG-mapper (v.2.1.9)^86^ pipeline with the eggNOG v.5.0.2 database^87^ with Diamond (v.2.0.11)^88^ and MMseqs2 release 12-113e3 (ref. ^89^). Enriched Kyoto Encyclopedia of Genes and Genomes (KEGG) biochemical reactions, along with associated enzymes and proteins, were identified on the basis of an adjusted *P*-value threshold of <0.01 and visualized using a heat map generated with ggplot2 v.3.4.2. The genes annotated with Gene Ontology (GO) classifications were subsequently extracted, and a GO enrichment analysis focusing on biological processes was conducted. This involved employing adaptive GO clustering in conjunction with Mann–Whitney *U* testing, utilizing the GO_MWU tool as previously described^91^. In this analytical approach, genes were ranked on the basis of signed log_2_ fold change values. Significantly enriched and depleted GO categories were determined by an adjusted *P* value < 0.05 (Supplementary Table 5). The most prominent enriched and depleted GO categories shared across comparisons were visualized in ggplot2 v.3.4.2 and coloured on the basis of the square root transformed delta rank values (enrichment score) of the GO categories (Supplementary Fig. 5). Similarly, enriched biochemical reactions annotated in the KEGG database, and proteins, were identified using a generalized linear model with an adjusted *P*-value threshold of <0.05 (Supplementary Tables 6 and 7). Prominently enriched and depleted categories shared across comparisons were visualized as heat maps (Supplementary Fig. 6).

### Metabolic network analysis and identification of a defined microbial community

For each MAG, we combined the predicted coding DNA sequence and corresponding translated amino acid sequences identified with FragGeneScanRs (v.1.1.0)^85^ with the functional annotations predicted by eggNOG-mapper (v.2.1.9)^86^, into genbank-formatted files using emapper2gbk (v.0.3.0)^92^ in ‘genes mode’. Subsequently, these files were utilized to generate the metabolic network of the fermentation community using the Metage2Metabo (v.1.5.3)^92,93^ graph-based metabolic analysis pipeline. Briefly, genome-scale metabolic networks (GSMNs) were reconstructed for the 44 MAGs detected in the fermenting bean using the Metage2Metabo (v.1.5.3)^92,93^ pipeline with Pathway Tools (v.26.0)^94^. The networks were then analysed to determine individual metabolic capabilities and, subsequently, the collective metabolic capabilities of the community. Metabolites known to be present in cocoa pulp (artificial pulp components: 0.14% (w/v) high-viscosity carboxymethyl cellulose, 0.77% (w/v) low-viscosity carboxymethyl cellulose, 1.09% (w/v) pectin, 2.5% (w/v) sucrose, 4% (w/v) glucose, 5% (w/v) fructose, 1% (w/v) citric acid, 0.5% (w/v) yeast extract, 0.5% (w/v) peptone, 0.1% (w/v) calcium lactate pentahydrate, 0.1% (v/v) Tween 80, 0.05% (w/v) magnesium sulfate heptahydrate and 0.02% (w/v) manganese sulfate monohydrate) (see refs. ^27,50^) were used as metabolic precursors to seed the network. The cooperation potential between GSMNs was assessed by calculating the added value of metabolic cooperation within the community. The added value of cooperation was used as the metabolic target to compute the key species and a defined community. Defined microbial communities were then identified by simplifying the complexity of the full community into a defined community with equivalent metabolic capabilities. The metabolites reachable by each MAG, identified on the basis of the cocoa pulp metabolic precursors seeded in the network, were compiled into a data matrix. This matrix was visualized using ComplexHeatmap v.2.12.1 with circlize v.0.4.15 (Supplementary Fig. 7). To visualize changes in the abundance of microbes with different metabolic potential over time, the data were transformed into a distance matrix using the dist() function in R^95^ with the ‘euclidean’ method and converted to two dimensions using classical multidimensional scaling of the dissimilarities with the stats v.4.3.0 package. Results were plotted with ggplot2 v.3.4.2 and coloured on the basis of the relative abundance of each MAG during the fermentation at each time point for each region (Extended Data Fig. 3a). Finally, our metabolic network analysis identified 10 MAGs possessing metabolic capabilities equivalent to the full community. The metabolites reachable by the 10 MAGs, based on the cocoa pulp as the precursor, were compiled and transformed into a distance matrix, then converted to two dimensions and plotted with ggplot2 v.3.4.2 as described above. We used ggvenn v.0.1.10 to visually represent the count of metabolites generated by the microbial communities, as well as to demonstrate the overlap of metabolites shared among them.

### Isolation of bacterial and fungal strains from fermenting cocoa beans for in vitro studies

We isolated bacterial and fungal strains from fermenting cocoa beans from the Cocoa Research Centre’s fermentation facility at the University of the West Indies in Trinidad. For this, mature ripe cacao pods were harvested from the International Cocoa Genebank, Trinidad (ICGT), and opened manually in the field by trained staff. The beans and surrounding pulp were scooped out by hand, placed into clean plastic bags and transported to the Cocoa Research Centre fermentation facility. The beans were placed into a pre-washed wooden fermentation box, covered with jute bags to minimize heat loss, and allowed to undergo natural fermentation at ambient temperatures ranging from a minimum of 22 °C (night-time) to a maximum of 34 °C (daytime). The beans were turned at 48 h and 96 h after fermentation initiation. Using sterile surgical gloves, two beans were collected daily from the fermentation mass at a depth of 7 cm at the centre of the fermentation box. These beans were cut into small pieces with a sterile scalpel blade. A few pieces of each bean were placed into tubes containing 80% glycerol, and the tubes were stored at −80 °C until further used. To culture the isolates, the bean–glycerol mixture was homogenized using a sterile scalpel. Samples (1 ml) of the homogenate were serially diluted (1/100, 1/1,000, 1/10,000, 1/100,000) with 10 mM MgCl_2_, from which 100-µl aliquots of the dilutions were plated on different selective agar media. The selective media included: (1) acetic acid medium (AAM): 1% (w/v) d-glucose (Sigma-Aldrich, G7021-5KG), 0.5% (v/v) ethanol, 0.3% (v/v) acetic acid, 1.5% (w/v) bacteriological peptone (Millipore) (Sigma-Aldrich, 91249-500 G), 0.8% (w/v) yeast extract (Millipore) (Sigma-Aldrich, 70161-500 G), 2% (w/v) agar (Sigma-Aldrich, A6686-500G), pH 4.5 with nystatin (30 mg l^−1^) (Sigma-Aldrich, N3503-25MU) and penicillin (50 mg l^−1^) (Sigma-Aldrich, 13752-5G-F); (2) De Man Rogosa Sharpe agar (MRS) (Millipore)^96^ (Sigma-Aldrich, 69964-500 G) with 0.1% (v/v) Tween 80 (Sigma-Aldrich, P4780-100ML) and nystatin (30 mg l^−1^); (3) yeast peptone glucose agar (YPG): 1% (w/v) yeast extract, 2% (w/v) peptone, 2% (w/v) glucose, 2% (w/v) agar, pH 5.6 with chloramphenicol (100 mg l^−1^) (Sigma-Aldrich, C1919-25G); and (4) nutrient agar (NA) (Millipore) (Sigma-Aldrich, 70148-500G). These media were optimized for the culture of the main taxonomic groups present in fermenting cocoa beans. The plates were incubated at various temperatures (25 °C, 30 °C, 37 °C and 42 °C) for 1–3 weeks. Colonies with distinct morphologies based on colony appearance, colour, optimal growth temperature and growth rate were selected and purified through successive subculturing until no visible signs of contamination were observed. The purified isolates were grown in liquid culture, mixed at a ratio of 1:1 with 80% (v/v) glycerol and stored at −80 °C for future use.

### Identification of isolates using Sanger sequencing

To identify the isolates, we conducted amplification and sequencing of the *16S rRNA* gene for bacteria and the internal transcribed spacer (ITS) region for fungi that were cultured. A single colony of each isolate was inoculated into LB media (500 µl) and incubated overnight at 28 °C with agitation at 200 r.p.m. Subsequently, 10 µl of the culture was heated at 95 °C for 5 min and then centrifuged at 10,000 × *g* for 1 min. The supernatant (1 µl) was used in the following PCR reaction mix: 5.4 µl Milli-Q water, 2 µl 5× Phusion HF buffer, 0.6 µl 25 mM MgCl_2_, 0.2 µl 10 mM dNTP, 0.2 µl DMSO, 0.25 µl of each primer (20 pmol µl^−1^), 0.05 µl Phusion High-Fidelity DNA polymerase (NEB, M0530L) and 1 µl DNA. The reactions were initially heated to 94 °C for 3 min, followed by 30 cycles of denaturation at 94 °C for 30 s, annealing at the optimized primer temperature (*16S rRNA*: 58 °C, ITS: 64 °C) for 30 s, extension at 72 °C for 1 min and 30 s, and a final extension at 72 °C for 10 min. The V1–V9 region of the bacterial *16S rRNA* gene was amplified with the 8F forward primer (5′-AGAGTTTGATCCTGGCTCAG-3′) and fD1 reverse primer (5′-ACGGCTACCTTGTTACGACTT-3′), while the ITS region was amplified using the ITS1 forward primer (5′-TCCGTAGGTGAACCTGCGG-3′) and ITS4 reverse primer (5′-TCCTCCGCTTATTGATATGC-3′). To confirm the specific amplification of target DNA regions, half of the PCR volume was visualized on an agarose gel via electrophoresis. The resulting amplicons were prepared for sequencing by combining 14 µl of Milli-Q water, 1 µl of the PCR reaction and 2 µl of sequencing primer (10 pmol µl^−1^). Sanger sequencing of the PCR products was conducted using the 8F and ITS1 primers for bacteria and fungi, respectively, and amplicons were sequenced at Eurofins Genomics.

### DNA extraction, library preparation and genome sequencing of selected isolates

To characterize the metabolic potential of the defined community employed in the in vitro fermentation experiment, we sequenced the full genomes of the selected isolates. This allowed us to characterize the individual metabolic capabilities of each isolate and, in turn, understand their collective metabolic capabilities. First, we extracted the total DNA from the isolates. To accomplish this, each isolate was cultivated in its respective medium. This involved inoculating 3 ml of the medium with a single colony and incubating under optimal conditions until saturation. The cells were collected through centrifugation at 4,000 × *g* for 10 min, and the supernatant was discarded. The collected cells were resuspended in 3 ml 10 mM MgCl_2_, subjected to centrifugation as before and finally resuspended in 1 ml 10 mM MgCl_2_. The suspension was transferred to a 2-ml tube with glass beads (150–212 μm and 425–600 μm in size) and centrifuged at 13,000 × *g* for 2 min. The supernatant was discarded and 1 ml of DNA extraction buffer (50 mM Tris-HCl pH 8.0, 5 mM EDTA pH 8.0, 350 mM sorbitol, 1% *N*-lauryl sarcosine, 71 mM NaCl, 0.1% CTAB) with 1 µl of Monarch RNase A (NEB, T3018L) was added. The cells were lysed in a Qiagen TissueLyser II Bead Mill (QIAGEN), which involved shaking at 30 Hz for 10 min. Subsequently, the sample was incubated at 60 °C for 20 min; then an equal volume of chloroform (Scientific Laboratory Supplies, CHE1574) was added. The sample was mixed by inverting several times and centrifuged at 13,000 × *g* for 5 min. The aqueous layer (top layer) was transferred to a new tube and an equal volume of ice-cold isopropanol was added. The tube was inverted several times and incubated at −20 °C overnight. Afterwards, the tube was centrifuged at 13,000 × *g* for 5 min, the supernatant was discarded, and the tube was inverted on tissue paper to allow the DNA pellet to air dry for 10 min. Following this, the DNA was resuspended in 50 µl of Milli-Q water. The suspension was centrifuged at 13,000 × *g* for 5 min, and the supernatant containing the DNA was transferred to a 1.5-ml tube and quantified using a Qubit fluorometer (Thermo Fisher). For the preparation of DNA libraries, the DNA was digested with NEBNext dsDNA fragmentase (NEB, M0348L) in the following reaction mix: 200 ng of DNA in 16 µl Milli-Q water, 2 µl 10× fragmentase reaction buffer v.2 and 2 µl NEBNext dsDNA fragmentase. The reactions were incubated at 37 °C for 20 min, and the process was halted by adding 5 µl 0.5 M EDTA pH 8. The volume was adjusted to 50 µl with Milli-Q water, and DNA fragments between 300 and 500 bp were selectively isolated using double-sided DNA selection with Agencourt AMPure XP beads (Beckman Coulter, A63881). Subsequently, the fragments were end repaired using a mixture comprising 30 µl DNA, 2.5 µl 3 U µl^−1^ T4 DNA polymerase, 0.5 µl 5 U µl^−1^ Klenow DNA polymerase, 2.5 µl 10 U µl^−1^ T4 PNK, 5 µl 10× T4 DNA ligase buffer with 10 mM ATP, 0.8 µl 25 mM dNTP mix and 8.7 µl Milli-Q water. After incubation at 20 °C for 30 min, the fragments were purified again using Agencourt AMPure XP beads. Following this, the DNA fragments were adenylated in a mix containing 34 µl of the end-repaired DNA, 3 µl 5 U µl^−1^ Klenow exo-, 5 µl 10× Enzymatics Blue buffer, 1 µl 10 mM dATP and 9 µl Milli-Q water. The mixture was incubated at 37 °C for 30 min, followed by 70 °C for 5 min, and then purified using Agencourt AMPure XP beads. Individual samples were indexed through ligation using a mix comprising 10.25 µl DNA, 1 µl 600 U µl^−1^ T4 DNA ligase, 12.5 µl of 2× Rapid Ligation buffer and 1.25 µl 2.5 µM indexing adapter from the KAPA Dual-Indexed Adapter kit (Kapa Biosystems, KK8722). Samples were incubated at 25 °C for 15 min; then 5 µl 0.5 M EDTA pH 8 was added. The fragments were purified twice with Agencourt AMPure XP beads and then enriched in the following reaction: 20 µl DNA, 25 µl of 2× KAPA HiFi HS Mix (Kapa Biosystems, KK2602), 2.5 µl 5 μM I5 primer (5′-AATGATACGGCGACCACCGAGATCTACAC-3′) and 2.5 µl 5 μM I7 primer (5′-CAAGCAGAAGACGGCATACGAGAT-3′). The reactions were initially heated to 98 °C for 45 s, followed by 14 cycles of 98 °C for 15 s, 60 °C for 30 s, and 72 °C for 30 s, with a final extension at 72 °C for 1 min. The resulting DNA libraries were purified using Agencourt AMPure XP beads, quantified on a Qubit fluorometer (Thermo Fisher), and equimolar quantities of individual barcoded DNA libraries were pooled and sequenced (PE150 bp) on an MGI Tech MGISEQ-2000 sequencing platform at Beijing Genomics Institute.

### Preparation of a defined community inoculum

A glycerol stock sample of each isolate was plated on the respective selective agar media and incubated at 30 °C for 72–96 h. Subsequently, a 15-ml tube containing 3 ml of selective medium was inoculated with a single colony from the agar plate. The tube underwent incubation at 30 °C with agitation at 80 r.p.m. for fungi, or 200 r.p.m. for bacteria, in a shaking incubator for 72–120 h. Following this incubation period, cells were collected by centrifugation at 4,000 × *g* at 4 °C for 8 min and subjected to three washes with 10 mM MgCl_2_ to remove residual media and cellular debris. The cells were then resuspended in 10 mM MgCl_2_, and the optical density at 600 nm (OD_600_) was measured to estimate cell concentrations. A pooled inoculum containing all isolates of the defined community was prepared, with the final concentration of each isolate in the pool set at 10^9^ colony-forming units per millilitre (c.f.u.s ml^−1^) assuming that 1 OD_600_ unit is equal to 10^9^ c.f.u.s ml^−1^. In addition, individual strains were systematically removed (single-strain dropout) from the 9-member microbial community to evaluate how the absence of each strain impacts overall community structure and function.

### In vitro fermentation set-up and sampling

Mature, ripe, healthy and undamaged cacao pods were harvested from the ICGT. The pods were thoroughly washed with water to dislodge any debris and surface sterilized using 20% hypochlorite solution containing 0.05% Triton X-100 for 10 min in a sterile hood. Afterwards, the pods were rinsed with sterile water and swabbed with 70% alcohol. The pods were carefully opened and the beans with pulp were extracted, maintaining sterile conditions. All beans from all the pods were pooled, mixed to homogeneity and divided into six sterile microboxes (Sac O2, TP1600 + TPD1600), each containing ~1 kg of beans. Three of the microboxes received inoculation with 100 µl of the defined synthetic community inoculum (SYNCOM), while the remaining three microboxes were inoculated with only 100 µl 10 mM MgCl_2_, serving as the No SYNCOM control. The beans were incubated for 96 h in a temperature-controlled incubator: 0–48 h at 30 °C, 48–72 h at 35 °C, and 72–96 h at 45 °C. Daily pH measurements were taken by collecting three beans from each fermentation. The testa/pulp were separated from the cotyledons, macerated in 10 ml distilled water using a mortar and pestle, and the pH of the suspensions was determined. For monitoring the microbial community during fermentation, swab samples of the beans were collected at 0, 48 and 96 h using a Zymo Collection Swab (R1104). Samples were collected in duplicate for each fermentation box and placed in Zymo DNA/RNA Shield Lysis and Collection tubes (Zymo, R1104). The tube contents were vigorously shaken for 10 s and the tubes were stored at −20 °C until used. Following the fermentation, the beans were spread on foil trays and placed in an oven at 35 °C for 5 days to reduce the moisture content to <7%. The beans were stirred on drying days 1, 2 and 3 to prevent bean clumping.

For the single-strain dropout experiments, 125 g of dried unfermented cocoa beans was sterilized with a 70% ethanol solution containing 1% Tween 20 and then rehydrated with sterile water. Excess water was discarded and the beans were transferred to a sterile microbox (Sac O2, TP1600 + TPD1600) containing 200 ml of sterile artificial pulp. The artificial pulp contained the following components: 0.14% (w/v) high-viscosity carboxymethyl cellulose (Sigma-Aldrich, C5678-1KG), 0.77% (w/v) low-viscosity carboxymethyl cellulose (Sigma-Aldrich, C5013-1KG), 1.09% (w/v) pectin (Sigma-Aldrich, P9135-500G), 2.5% (w/v) sucrose (Sigma-Aldrich, S0389-1KG), 4% (w/v) glucose (Sigma-Aldrich, G7021-5KG), 5% (w/v) fructose (Sigma-Aldrich, F0127-1KG), 1% (w/v) citric acid (Sigma-Aldrich, C0759-1KG), 0.5% (w/v) yeast extract (Millipore) (Sigma-Aldrich, 70161-500G), 0.5% (w/v) peptone (Millipore) (Sigma-Aldrich, 91249-500G), 0.1% (w/v) calcium lactate pentahydrate (Sigma-Aldrich, C8356-250G), 0.1% (v/v) Tween 80 (Sigma-Aldrich, P4780-100ML), 0.05% (w/v) magnesium sulfate heptahydrate (Sigma-Aldrich, M2773-1KG) and 0.02% (w/v) manganese sulfate monohydrate (Sigma-Aldrich, M7899-500G), adjusted to pH 3.6. The experimental design included a full synthetic microbial consortium (T1, 9-member SYNCOM) and modified versions where individual strains were removed (T2–T10). Control groups consisted of non-inoculated beans (T11 and T12) and beans inoculated with a randomly selected 9-member microbial consortium (T13). For each treatment, four independent fermentations were performed. The beans were incubated for 120 h in a temperature-controlled incubator under the following conditions: 0–48 h at 30 °C, 48–72 h at 35 °C, and 72–120 h at 45 °C. However, for T12, the beans were maintained at a constant temperature of 30 °C for the entire 120-h incubation period. pH measurements of the testa/pulp and cotyledons were recorded daily from a single bean of each fermentation. Swab samples for microbial community analysis were collected at 0, 24 and 48 h (*n* = 156), while 5 beans from each treatment replicate were sampled at 0, 48 and 120 h for metabolomic analysis (*n* = 156). After fermentation, beans from each treatment replicate (*n* = 52) were oven dried at 35 °C, as previously described, to produce cocoa liquors. In addition, total cell counts in the 9-member SYNCOM were measured using a BlauBrand Thoma counting chamber (Brand). To assess potential growth limitations, whether due to nutrient deficiencies in the pulp or environmental stresses from temperature and/or pH, each isolate was cultured in artificial pulp at pH values of 3.6, 4.6, 5.6 and 6.6. Cultures were incubated under three fermentation temperature conditions (30 °C, 35 °C and 45 °C) for 70 h with 200 r.p.m. agitation. Growth was monitored across all conditions by measuring OD_600_.

### DNA extraction from in vitro fermentation samples

The DNA samples were placed in a Qiagen TissueLyser II Bead Mill (QIAGEN) and homogenized at 30 Hz for 10 min. Following this, DNA was extracted using the ZymoBIOMICS DNA Miniprep kit (Zymo, D4300) following manufacturer instructions, and the resulting DNA concentration was determined using a Qubit fluorometer (Thermo Fisher).

### Bacteria *16S rRNA* library preparation and sequencing

We amplified the V3–V4 highly variable region (~480 bp) of the bacterial *16S rRNA* gene using the 338F (5′-ACTCCTACGGGAGGCAGCA-3′) and 806R (5′-GGACTACHVGGGTWTCTAAT-3′) universal primer sequences. Unique frameshifting tags were added to the 5′ end of both primers following the method outlined in ref. ^97^ to enhance library diversity and enable efficient multiplexing of samples for sequencing. Each sample was amplified in triplicate, and for each 96-well PCR plate of reactions, three unique sets of frameshifting tag combinations were employed with both the forward and reverse primers. This approach facilitated the effective multiplexing of samples for sequencing across multiple plates. The reaction mix for each sample included 1 µl DNA, 5 µl 2× KAPA HiFi HS Mix (Kapa Biosystems, KK2602), 0.25 µl of 338F forward primer frameshift mix (10 pmol µl^−1^), 0.25 µl of 806R reverse primer frameshift mix (10 pmol µl^−1^) and 3.5 µl Milli-Q water. The amplification protocol involved an initial heating step at 94 °C for 3 min, followed by 24 cycles of 94 °C for 30 s, 50 °C for 30 s, and 72 °C for 30 s, with a final extension at 72 °C for 10 min. The PCR products from the triplicate reactions were combined and purified using Agencourt AMPure XP beads (Beckman Coulter, A63881). Subsequently, the PCR products were indexed using 96 unique reverse indexing primers. The indexing mix for each sample included 4.5 µl PCR product DNA, 5 µl 2× KAPA HiFi HS Mix (Kapa Biosystems, KK2602), 0.25 µl forward enrichment primer (10 pmol µl^−1^) and 0.25 µl reverse enrichment-indexing primer (10 pmol µl^−1^). The forward enrichment primer used was (5′-AATGATACGGCGACCACCGAGATCTACACGCCTCCCTCGCGCCATCAGAGATGTG-3′), and the reverse enrichment-indexing primer was the TruSeq Read 2-annealing reverse Illumina adapter compatible with the Illumina MiSeq platform. The indexing procedure involved an initial heating step at 94 °C for 3 min, followed by 9 cycles of 94 °C for 30 s, 60 °C for 30 s, and 72 °C for 30 s, with a final extension at 72 °C for 10 min. The DNA libraries were purified using Agencourt AMPure XP beads (Beckman Coulter, A63881) and quantified with a Qubit fluorometer (Thermo Fisher). Subsequently, the libraries were pooled in equal amounts and diluted to 10 pM for sequencing. The sequencing process (PE300) was conducted on an Illumina MiSeq instrument using the Reagent Kit V3 600-cycle (Illumina) at the DeepSeq Sequencing Facility at the University of Nottingham.

### Fungi ITS library preparation and sequencing

For fungal profiling, the ITS2 region was amplified and sequenced using the ITS3-F (5′-GCATCGATGAAGAACGCAGC-3′) and ITS4-R (5′-TCCTCCGCTTATTGATATGC-3′) universal primer sequences described in ref. ^98^. To enhance library diversity and facilitate sample multiplexing for sequencing, unique frameshifting tags were incorporated at the 5′ end of both primers using the methodology outlined in ref. ^97^. Every sample was subjected to triplicate amplification. In addition, for each 96-well PCR plate of reactions, three distinct sets of unique frameshifting tag combinations of the forward and reverse primers were used to enable the multiplexing of samples for sequencing from multiple plates. The reaction mix for each sample comprised 1 µl DNA, 5 µl 2× KAPA HiFi HS Mix (Kapa Biosystems, KK2602), 0.25 µl ITS3-F forward primer frameshift mix (10 pmol µl^−1^), 0.25 µl ITS4-R reverse primer frameshift mix (10 pmol µl^−1^) and 3.5 µl Milli-Q water. The amplification protocol began with an initial heating step at 94 °C for 3 min, followed by 24 cycles of 94 °C for 30 s, 55 °C for 30 s, and 72 °C for 30 s, concluding with a final extension at 72 °C for 10 min. Amplicons from the triplicate reactions were consolidated and purified using Agencourt AMPure XP beads (Beckman Coulter, A63881). Subsequently, the samples were indexed using the TruSeq Read 2-annealing reverse Illumina adapter, pooled as previously described (see ‘Bacteria *16S rRNA* library preparation and sequencing’) and sequenced on an Illumina MiSeq platform using the 600-cycle V3 Reagent kit (Illumina) at the University of Nottingham’s DeepSeq Sequencing Facility.

### Processing of cocoa beans and liquors for sensory analysis and metabolomics

For the validation of the defined community experiment, dried beans from the three 9-member SYNCOM inoculated fermentations were combined into a single pool, while the three No SYNCOM control batches were pooled separately. The pooled beans were then processed into cocoa liquors and subjected to sensory evaluation as described previously (see ‘Drying, roasting and sensory evaluation of liquor samples’), and well as to gas chromatography–mass spectrometry (GC–MS) analysis. For the single-strain dropout experiment, cocoa beans collected at 0, 48 and 120 h of fermentation were freeze dried for 72 h and ground into a fine powder for liquid chromatography–mass spectrometry (LC–MS) and GC–MS analysis. In addition, the dried fermented beans were processed into cocoa liquors and analysed using sensory evaluation and GC–MS.

### Characterization of volatile compounds in cocoa beans and liquors

The aroma and other volatile compounds in the cocoa bean and liquor samples were analysed at the International Flavour Research Centre (IFRC) at the University of Nottingham. Milled powder (1 g) of the cocoa samples was mixed with 10 µl 3-heptanone (0.01 μg µl^−1^) internal standard in hermetically sealed 20-ml vials and incubated for 5 min at 50 °C in a thermostatic agitator. A 50/30 μm DVB/CAR/PDMS SPME Fibre (Supelco) was used to extract volatile compounds from the headspace of each sample. The SPME fibre extracted for 15 min at 50 °C and desorbed for 0.5 min at 240 °C. The volatiles were analysed by GC–MS using splitless injection into a TRACE 1300 series gas chromatograph coupled with a single quadrupole mass spectrometer (Thermo Fisher). A ZB-WAX-plus column of 30 m length, 0.25 mm internal diameter and 0.250 μm film thickness (Phenomenex) was used with the following time–temperature programme: 40 °C for 2 min, followed by a temperature increase from 40–240 °C at a rate of 6 °C min^−1^, and then held at 240 °C for 5 min. A minimum of 3 replicates per liquor sample were analysed with randomized sample injections for the validation of the defined community experiment, including 9-member SYNCOM-inoculated samples, No SYNCOM samples, Santander, Huila, Antioquia and reference liquors. For the single-strain dropout experiment, individual biological replicates were utilized. The SPME fibre was conditioned for 3 min at 240 °C between samples. The quality of the headspace GC–MS runs was assessed by running the internal standard after 5–20 consecutive sample runs and estimating the variations in retention time and peak areas. Volatile compounds were identified by comparing each mass spectrum with either the spectra from standard compounds or with spectra in reference libraries (NIST/EPA/NIH Mass Spectral Library). The relative abundance of volatiles was calculated from GC peak areas by comparison with the peak area of the internal standard.

### Characterization of non-volatile compounds in cocoa beans

A 100-mg portion of the powdered sample was weighed and placed into a 1.5-ml microcentrifuge tube. The sample was defatted by adding 800 µl ice-cold hexane, vortexing and incubating in a sonic water bath. The mixture was centrifuged and the supernatant was discarded. This defatting process was repeated twice more, and the defatted pellet was dried using nitrogen gas with a sample concentrator (Techne). To extract metabolites, 460 µl 80% methanol was added to the dried pellet, and the mixture was vortexed, sonicated and centrifuged. The supernatant containing the metabolites was saved and the extraction was repeated twice more. The combined supernatant was centrifuged again and the final supernatant was transferred to a new tube. The extract was dried using a Savant SpeedVac SPD140DDA vacuum concentrator (Thermo Scientific) and then stored at −20 °C. Before analysis, the dried pellet was reconstituted with 50% aqueous ethanol, followed by sonication. The sample was centrifuged and the supernatant was transferred to a new 1.5-ml tube. For each time point (0, 48 and 120 h), aliquots from biological replicates of each treatment were pooled separately, transferred to LC–MS vials, capped and stored for further analysis. The generation of the untargeted metabolic profiles was performed using an Agilent 1260 Infinity II Ultra High-Performance Liquid Chromatography system coupled to an Agilent 6546 tandem quadrupole time-of-flight mass spectrometer (Agilent Technologies). Chromatographic separation was performed with an Acuity UPLC HSS T3 column (2.1 × 100 mm, 1.8 μm; Waters) fitted with a KrudCatcher pre-filter (Phenomenex). The flow rate of the mobile phase (A: 5% acetonitrile, versus B: 95% acetonitrile, both with 0.1% formic acid v/v) was at 0.3 ml min^−1^, with the analytical gradient starting at 5% solvent B, increasing to 15%, 25%, 35%, 45% and 65% at 2, 4, 8, 10 and 12 min, respectively, followed by column washing and re-equilibration (total run time 22 min). Quality control (QC) samples were made by pooling samples from all treatments. After injection of ×10 QC samples to condition the system, each sample was randomized to ×5 injections across the batch. QC samples were injected after every 10 consecutive runs to assess system performance across the batch. Data were collected in MS1 mode scanning 50–1,700 *m*/*z*. Reference masses were continuously injected for mass correction.

### Analysis of *16S rRNA* and ITS regions from Sanger sequencing for isolate identification

To identify the bacterial and fungal isolates cultured, we sequenced the *16S rRNA* gene from the bacteria and the ITS region from the fungal isolates. Initially, low-quality bases were trimmed from the sequences, and the results were searched on the National Centre for Biotechnology Information (NCBI) nucleotide database using the Basic Local Alignment Search Tool (BLAST v.2.12.0)^99^ to determine the taxonomy of the species. We performed multiple sequence alignments with the 16S rRNA and ITS sequences for the bacteria and fungi, respectively, using DECIPHER v.2.24.0. The alignments were trimmed with microseq v.2.1.6, transformed into distance matrices with seqinr v.4.2.16, and neighbour-joining trees were constructed with ape v.5.6.2. The resulting trees, based on the bacterial *16S rRNA* sequences (Extended Data Fig. 3b) and fungi ITS sequences (Extended Data Fig. 3c), were visualized using ggtree v.3.8.0 with ggtreeExtra v.1.10.0. To assess the overall relative abundance of our collection representing the cocoa fermentation microbiome, we used the tax_glom() function within phyloseq v.1.44.0 to aggregate taxa from the microbiome dataset of the three Colombian fermentations to the family level. Subsequently, we computed the mean relative abundance of each family at each fermentation time point and plotted the results using ggplot2 v.3.4.2.

### Genome assembly, annotation and construction of the isolates metabolic network

We used Cutadapt (v.4.6)^100^ to eliminate primer and barcode sequences, as well as low-quality sequences, from the paired-end reads of the sequenced genomes of the isolates. Subsequently, the high-quality filtered reads were de novo assembled into a draft genome for each isolate using SPAdes (v.3.15.5)^101^ with default parameters. The assembled genomes were evaluated for contiguity and completeness using BUSCO (v.5.6.1)^102^. Open reading frames in the genomes were predicted with FragGeneScanRs (v.1.1.0)^85^ with default settings. Functional annotation of the predicted proteins was carried out using the eggNOG-mapper (v.2.1.9)^86^ pipeline, utilizing the eggNOG v.5.0.2 database^87^ with Diamond (v.2.0.11)^88^ and MMseqs2 release 12-113e3 (ref. ^89^). The predicted coding DNA and translated amino acid sequences, along with the predicted functional annotations, were combined into genbank-formatted files using emapper2gbk (v.0.3.0)^92^ in ‘genes mode’. Subsequently, the Metage2Metabo (v.1.5.3)^92,93^ pipeline was used to generate the metabolic network of the isolates used in the defined community. Metabolites reachable by each of the isolates in the network, based on the cocoa pulp metabolites as the precursor, were compiled into a data matrix and visualized as described previously (see ‘Metabolic network analysis and identification of a defined microbial community’).

### *16S rRNA* and ITS amplicon sequence data processing

Raw reads were demultiplexed and trimmed with Cutadapt (v.4.6)^100^. Subsequently, the processed sequences were denoised and collapsed into amplicon sequence variants (ASVs) using the DADA2 v.1.24.0 pipeline. In brief, paired reads were filtered by removing sequences with uncalled bases, eliminating reads with >2 expected errors, and truncating reads when the average quality score dropped to <2. Error rates for forward and reverse reads were determined separately through the learnErrors() function. These error rates were then utilized to infer ASVs individually for both the forward and reverse reads, and the forward and reverse sequences were subsequently merged. The merged ASVs were used to construct an ASV sequencing table, and chimaeras were removed. Bacteria ASVs were classified using the SILVA 138 database^103^, while the fungi ASVs were classified using the UNITE v.9 database^104^. Functions in phyloseq v.1.44.0 with microbiome v.1.22.0 and microbiomeutilities v.1.0.17 were used to filter the dataset and remove samples with low read depth, remove unidentified taxa and singletons, transform abundance values using rarefaction, subset and merge sample and taxonomic groups, and perform other dataframe manipulations. To assess alpha diversity across the samples, we calculated the Shannon diversity index using phyloseq v.1.44.0. We used ANOVA to test for significant differences in Shannon diversity indices between groups, and means were separated using Tukey’s HSD test in the agricolae v.1.3.5 R package. For beta diversity, Bray–Curtis dissimilarity matrices were calculated using the phyloseq v.1.44.0 ‘bray’ method, and the variances explained by treatment and fermentation time were estimated by performing PERMANOVA using the adonis2() function in the vegan v.2.6.4 R package. Constrained ordination of beta-diversity was plotted using CAP on the basis of Bray–Curtis dissimilarity matrices calculated with vegan v.2.6.4. We visualized differences in treatment and time with the CAP analysis, using the following models:

10 $$\sim {\rm{treatment}}+{\rm{condition}}({\rm{time}}+{\rm{replicate}})$$

11 $$\sim {\rm{time}}+{\rm{condition}}({\rm{treatment}}+{\rm{replicate}})$$

PCoA based on Bray–Curtis dissimilarities was used to visualize shifts in microbial community composition across fermentation treatments (T1–T13). Bar plots showed the Euclidean distance between each treatment centroid and T1 (the full synthetic community), reflecting the degree of dissimilarity from the baseline. The relative abundance of taxa was plotted as a stacked bar representation using phyloseq v.1.44.0. The tax_glom() function in phyloseq v.1.44.0 was used to agglomerate taxa, and the aggregate_rare() function in microbiome v.1.22.0 was used to aggregate rare groups.

### Analysis of volatile compounds in cocoa beans and liquors

The data were first pre-processed, followed by analysis in R. PCA was performed using Euclidean distances with the prcomp() function, while PCoA was conducted using a dissimilarity matrix computed with the Manhattan distance in the dist() function. Classical multidimensional scaling was then applied using the cmdscale() function. The results were visualized using ggplot2 v.3.4.2. A bar plot of Manhattan distances between each treatment centroid and T1 (the full synthetic community) was generated to quantify dissimilarity from the baseline. To identify enriched volatile compounds among samples, we employed DESeq2 (v.1.40.0)^65^, fitting a GLM with the following design:

12 $$\begin{array}{l}{\rm{relative}}\;{\rm{abundance}}\;{\rm{of}}\;{\rm{volatile}}\;{\rm{compound}}\\\sim{\rm{cocoa}}\;{\rm{liquor}}+{\rm{replicate}}\end{array}$$

13 $$\begin{array}{l}{\rm{relative}}\;{\rm{abundance}}\;{\rm{of}}\;{\rm{volatile}}\;{\rm{compound}}\\\sim{\rm{treatment}}+{\rm{fermentation}}\;{\rm{time}}+{\rm{replicate}}\end{array}$$

From the fitted model, we extracted key comparisons. A volatile compound was deemed significant if it exhibited an FDR-adjusted *P* value (*q* value) < 0.05. The GLM analysis results were visualized in a heat map, with the colours representing the log_2_ fold change generated by the GLM. Black squares were used to highlight significant differences (*q* value < 0.05) with log_2_ fold change > ±2 between the aforementioned comparisons.

### Analysis of non-volatile compounds in cocoa beans

For data analysis, the total ion chromatograms of repeat QC injections were visually assessed to check comparability of runs throughout the dataset. Initially, global MS1 features (peak height >20,000) were first extracted using Mass Profiler software (MP; v.10 Agilent Technologies) and exported to a common .CEF file for each polarity. Thereafter, files for each replicate group were time aligned to the central QC sample in Profinder software (v.10, Agilent); then features were extracted (peak height >5,000) in ‘batch targeted’ mode using the global MS1 features .CEF file as a reference library. Following this pre-processing, PCoA was conducted using Euclidean distances in vegan v.2.6.4. The dissimilarity matrix was computed with the vegdist() function and then used to perform PCoA. The results were visualized using ggplot2 v.3.4.2. A bar plot depicting the Euclidean distances between each treatment centroid and T1 (the full synthetic community) was created to measure dissimilarity from the baseline. We employed DESeq2 (v.1.40.0)^65^ to discern enriched compounds among samples. This was achieved by fitting a GLM with the design:

14 $$\begin{array}{l}{\rm{relative}}\;{\rm{abundance}}\;{\rm{of}}\;{\rm{non}}-{\rm{volatile}}\;{\rm{compound}}\\\sim{\rm{treatment}}+{\rm{fermentation}}\;{\rm{time}}+{\rm{replicate}}\end{array}$$

From the fitted model, we identified key comparisons between treatments and fermentation times, highlighting significant differences where *q* values were <0.05 and log_2_ fold changes exceeded ±2. The results of the GLM analysis were visualized in a heat map, with colours representing the log_2_ fold change values generated by the model.

### Reporting summary

Further information on research design is available in the Nature Portfolio Reporting Summary linked to this article.

## Supplementary information

Supplementary Information Supplementary Figs. 1–9 and Results 1–15, and captions of Supplementary Tables 1–8.

Reporting Summary

Supplementary Tables Supplementary Table 1. *Theobroma cacao* SNP panel used to genotype cacao trees sampled from cocoa plantations located in the Santander, Huila and Antioquia regions in Colombia. Table 2. SNP profiles of *Theobroma cacao* trees assessed from cocoa plantations in this study. Table 3. Feature importance of abiotic and biotic markers associated with flavour attributes in chocolate from Colombia and Trinidad cocoa bean fermentations. Table 4. Genome and quality statistics of metagenome assembled genomes (MAGs) constructed from metagenomic datasets originating from cocoa plantations in Santander, Huila and Antioquia. Table 5. Enriched GO categories across microbial communities during cocoa bean fermentation. Table 6. Enriched KEGG reactions across microbial communities during cocoa bean fermentation. Table 7. Enriched proteins across microbial communities during cocoa bean fermentation. Table 8. Characterization of bacterial and fungal isolates using 16S rRNA and ITS amplicon sanger sequencing.

## Data Availability

Nanopore sequencing data, as well as 16S rRNA and ITS amplicon sequencing data generated for this study, have been archived in the NCBI Sequence Read Archive under project accession PRJNA1104253. Microbial genomes and metagenomes produced in this work are publicly available via https://www.gabrielcastrillo.com/ and through Zenodo at 10.5281/zenodo.15985598 (ref. ^105^), under the Trinidad Isolate Genomes Repository and Colombia MAGs Repository. Datasets required to reproduce the results of this study are available in the associated GitHub repository at https://github.com/David-Lee86/min-com-choc (ref. ^106^). Reference genomes used include *Theobroma cacao* Criollo v.2.0 (Cocoa Criollo B97-61/B2 version 2; https://cocoa-genome-hub.southgreen.fr/download) and *Homo sapiens* GRCh38.p14 (RefSeq GCF_000001405.40). The strain collection used in this study is available upon request by contacting G.C. (gabriel.castrillo@nottingham.ac.uk).

## Code availability

Scripts, source data and metabolic models required to reproduce the analyses of this study can be accessed through GitHub at https://github.com/David-Lee86/min-com-choc (ref. ^106^).
