# Supplementary material for: A defined microbial community reproduces attributes of fine flavour chocolate fermentation
Source: Nat Microbiol. 2025 Aug 18;10(9):2130–52. doi: 10.1038/s41564-025-02077-6 (PMC12408344; doi:10.1038/s41564-025-02077-6)
Supplement: Supplementary file 1 — Supplementary Figs. 1–9 and Results 1–15, and captions of Supplementary Tables 1–8. [file 41564_2025_2077_MOESM1_ESM.pdf]

nature microbiology

Supplementary information https://doi.org/10.1038/s41564-025-02077-6

A defined microbial community reproduces attributes of fine flavour chocolate fermentation

In the format provided by the authors and unedited

Supplementary Fig. 1

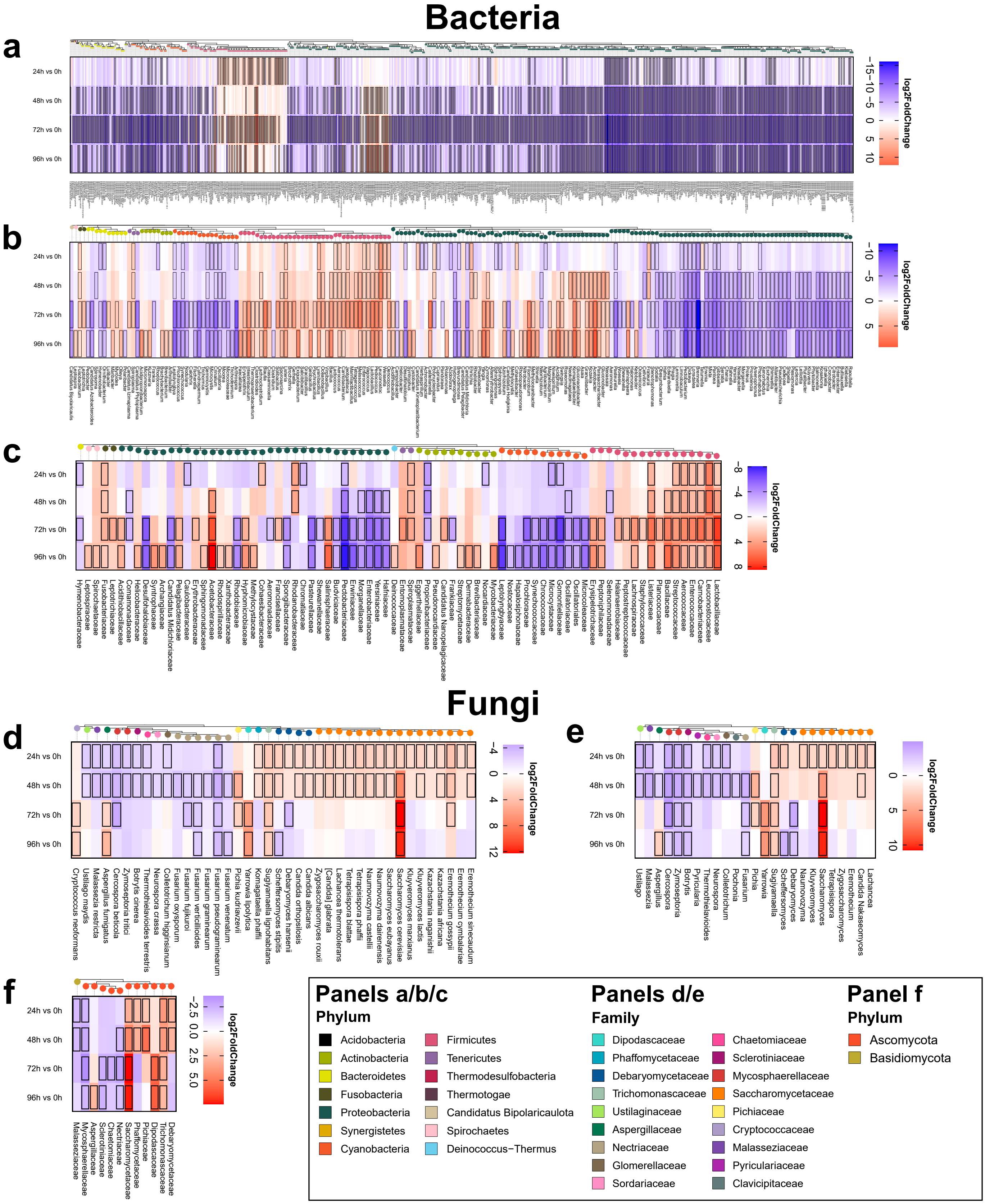

Supplementary Fig. 1. The enrichment profiles of bacteria and fungi change during the progression of cocoa beans fermentation. Heatmaps showing microbial enrichment profiles at different fermentation time points compared to the start of fermentation (0 h) at different taxonomic units for bacteria **a.** Species, **b.** Genus, **c.** Family, and fungi **d.** Species, **e.** Genus, **f.** Family. Heatmaps are coloured based on $\log_2$ fold changes derived from a generalised linear model contrasting the abundance of each taxonomic unit in a given fermentation time point against the start of the fermentation. Tiles outlined in black denote statistically significant enrichment (red) or depletion (blue) ($q < 0.05$) with a $\log_2$ fold change > ± 2 with respect to the start of the fermentation. Heatmaps are clustered based on taxonomic classification of the bacteria or fungi.

Supplementary Fig. 2

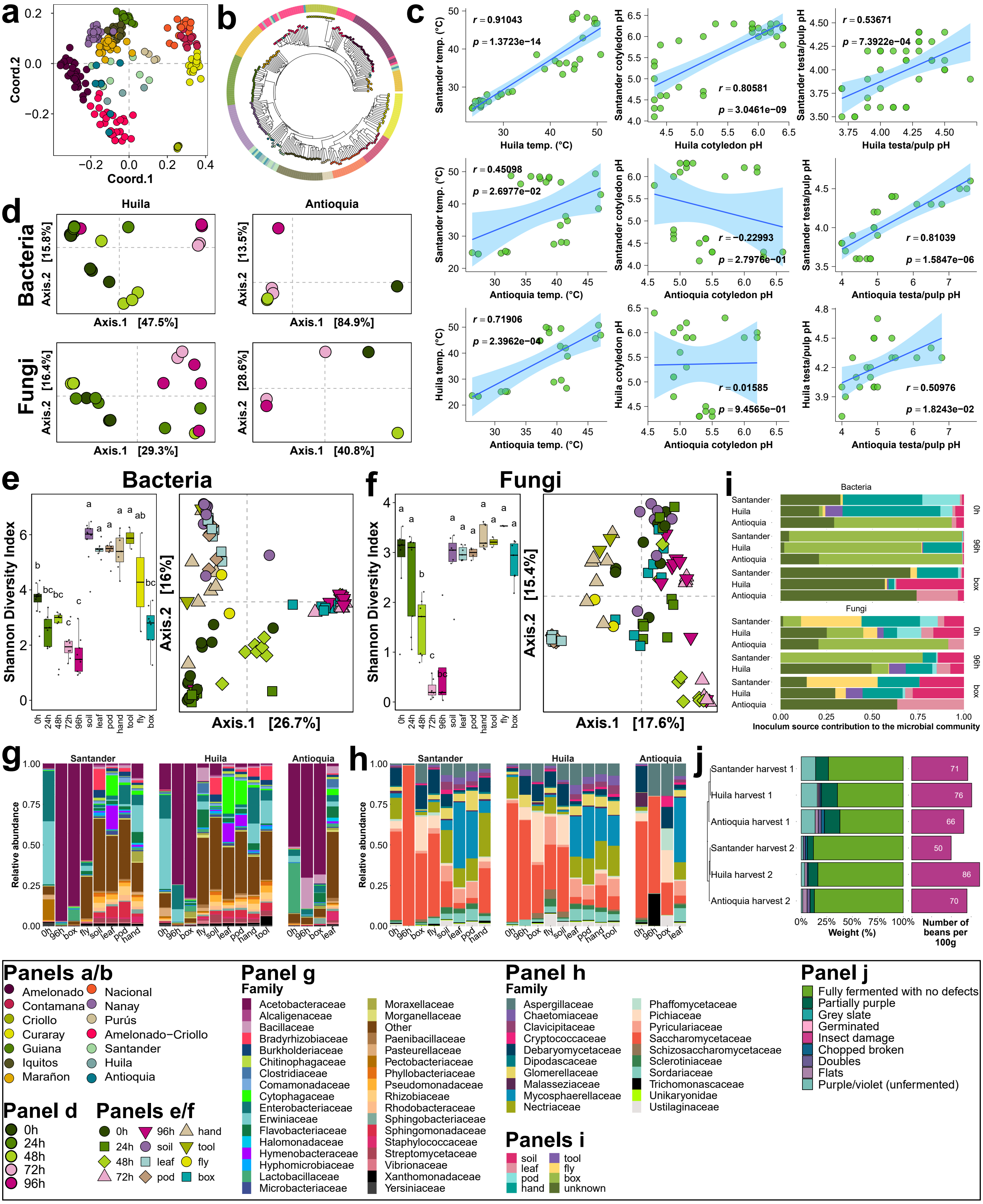

Supplementary Fig. 2. The changes in the abiotic and biotic characteristics of cocoa bean fermentation are linked to chocolate attributes. a. Principal coordinate analyses (PCoA) showing the projected genomic characteristics of *Theobroma cacao* trees from Santander, Huila, and Antioquia plantations. The trees were genotyped and 84 high-quality SNP markers were generated. The analysis also included SNP profiles from reference cocoa accessions. Each point represents a single tree. **b**. Neighbour-Joining tree depicting relationships between the Colombian cacao varieties from Santander, Huila, and Antioquia plantations and the reference cacao accessions. The tree was constructed using the 84 SNP markers identified. The tree tips and outer ring are coloured by genetic clusters or tree location in Colombia **c**. Pearson correlation analysis between bean temperature (temp.), cotyledon pH, and testa/pulp pH recorded during fermentations in Santander, Huila and Antioquia. The relationship was assessed in each plot using a two-sided Pearson correlation test. A linear regression line with a shaded band represents the 95% confidence interval. The Pearson correlation coefficient (r) and associated p-value are shown, with individual data points displayed as dots. **d**. PCoA plots showing the projected microbiota (bacteria and fungi) composition in each farm fermentation: Huila and Antioquia. **e**. Graphs show alpha (left) and PCoAs beta (right) diversities estimated of bacterial and **f**. fungal communities in fermenting cocoa beans and surrounding farm environmental samples from the three farms. Microbial DNA samples were collected from five independent fermentation trials: two trials during the mid-harvest (May), one in Santander and one in Huila, and three trials during the main harvest (October–November), one each in Santander, Huila, and Antioquia. During each trial, two microbial samples were collected daily from the fermenting mass. Additional samples were obtained from various environmental sources on the farms, including cacao leaves, pods, the fermentation box, farm workers' hands, tools, soil, and fruit flies. After quality filtering, 93 bacterial (n = 93) and 80 fungal (n = 80) community profiles were retained for analysis. Alpha diversity was assessed using the Shannon Diversity Index. Boxplots illustrate the distribution of Shannon Index values across fermentation and environmental samples. The horizontal line within each box represents the median; box edges indicate the interquartile range (25th to 75th percentile); whiskers extend to the smallest and largest values within 1.5 times the interquartile range, and individual data points, including outliers, are overlaid as dots. One-way analysis of variance (ANOVA) was used to assess significant differences among groups (bacteria: p = 3.1796e-28; fungi: p = 1.1662e-14), with means separated using Tukey's post hoc test. Groups sharing the same letter are not significantly different (p > 0.05); groups with different letters differ significantly (p < 0.05). Beta diversity was visualised using a PCoA based on Bray-Curtis dissimilarities. **g**. Phylogram displays the relative abundance profiles of main bacterial and **h**. fungal families at early and late fermentation time points and the surrounding farm environmental samples. **i**. Bar plots depict the inoculum source contributions to bacterial and fungal communities at 0h and 96h of fermentation, as well as the fermentation box across the different farms. **j**. Bar plots showing the quality of fermented and dried cocoa beans from Santander, Huila and Antioquia, assessed based on 100 g samples. The composition, by percentage weight of each lot, for the quality parameters evaluated, is presented on the left bars, while the number of beans per 100 g is shown on the right bars. Bar plots are clustered based on bean quality of lots.

Supplementary Fig. 3

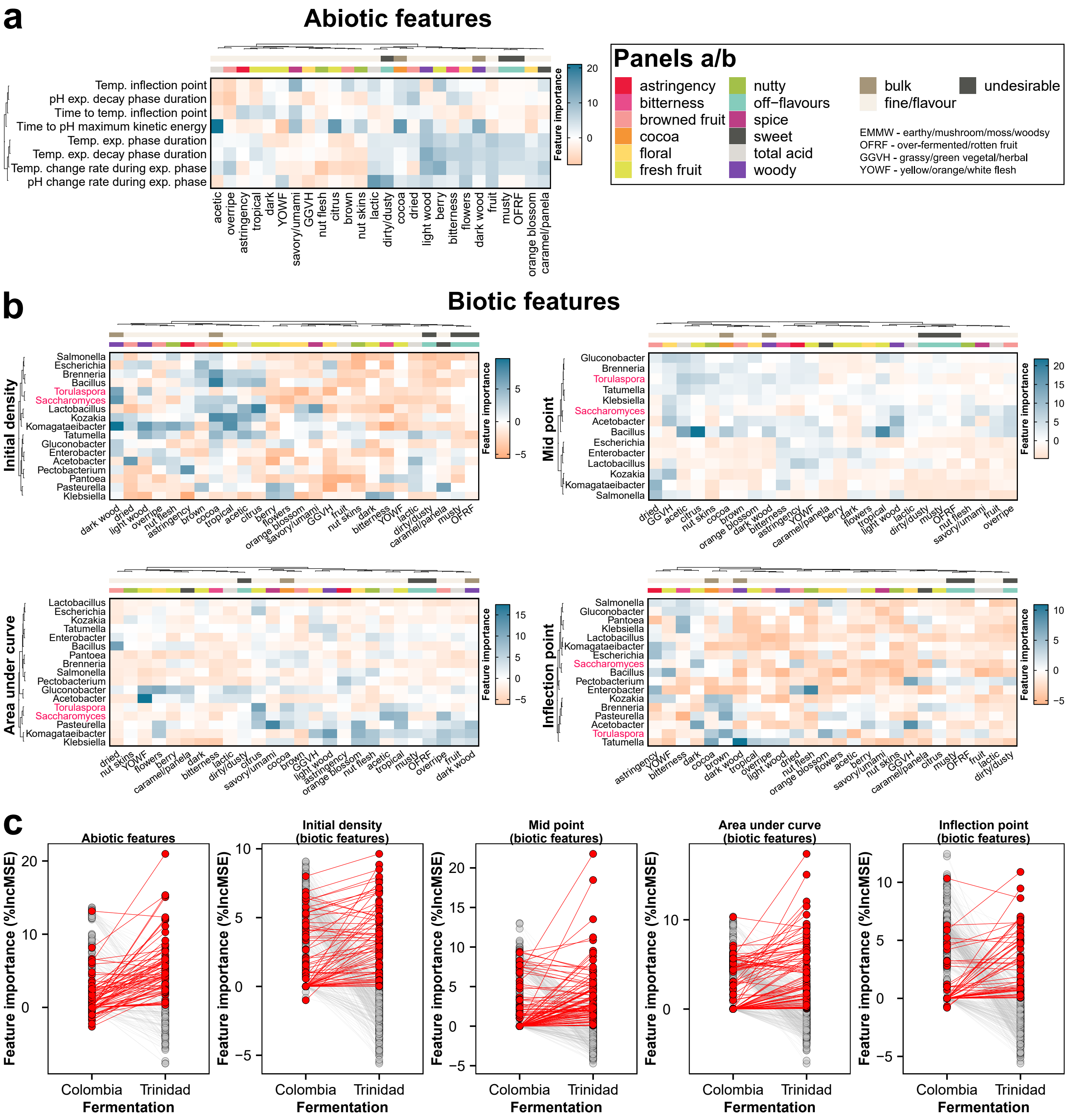

Supplementary Fig. 3. Abiotic and biotic markers identified are associated to chocolate flavour attributes in multiple fermentations across agroecological regions. a. Heatmap depicting extracted features from temperature and pH kinetic curves of 19 different cocoa beans fermentations, emphasizing their association with specific chocolate sensory attributes. Kinetic curves were modelled using the Practical Program for Forces Modelling tool. Hierarchical clustering was applied to the heatmap based on sensory attributes and selected temperature and pH features. Feature importance, quantified by the percentage increase in mean squared error (%IncMSE) derived from the Random Forest model, is represented by a colour gradient. The top bars indicate sensory groups as defined by the Cocoa of Excellence guidelines for cocoa liquors and chocolate, differentiating attributes typical of bulk cocoa, fine/flavour cocoa, and undesirable flavours. **b**. Heatmaps showcasing extracted features from key bacterial and fungal growth curves in 11 independent cocoa beans fermentations, demonstrating their impact on individual sensory attributes. Growth curves were plotted, and kinetic features were extracted. Feature importance (%IncMSE) was calculated using the Random Forest model and is displayed using a colour gradient. The heatmap is hierarchically clustered based on sensory attributes and selected taxa features, with bacterial taxa labelled in black and fungal taxa in red. The top bars denote sensory groups according to the Cocoa of Excellence guidelines, distinguishing flavour attributes associated with bulk cocoa, fine/flavour cocoa, and undesirable flavours. **c**. Features from panels a and b with consistent Feature Importance (%IncMSE) across Colombia and Trinidad fermentations. Red-highlighted points represent features from Trinidad fermentations with at least 70% of the corresponding Feature Importance (%IncMSE) value from the Colombia fermentation.

Supplementary Fig. 4

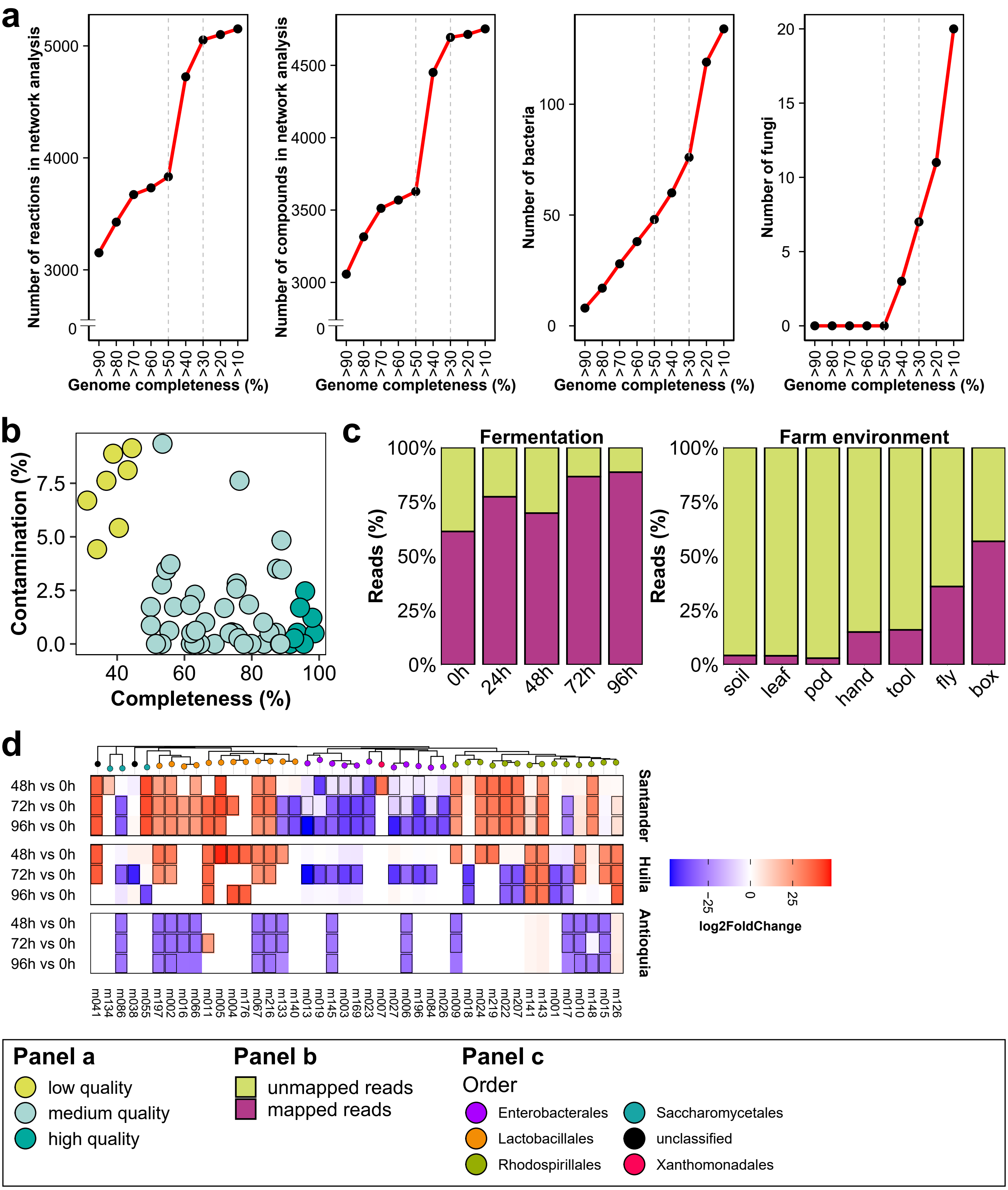

Supplementary Fig. 4. The identified metagenome assembled genomes (MAGs) recapitulate the microbial dynamics found in cocoa bean fermentations. **a**. Impact of genome completeness thresholds on metabolic network features derived from MAGs. Line plots depict the cumulative number of metabolic reactions, compounds, bacterial MAGs, and fungal MAGs retained at increasing genome completeness thresholds (from >10% to >90%). This analysis highlights the trade-off between MAG quality and coverage in the metabolic network analysis. **b**. Scatter plot illustrating the completeness and contamination levels of 55 selected MAGs, filtered for contamination <10%, and completeness >50% for bacteria or >30% for fungi. MAGs are colored by quality category: low (yellow), medium (blue), and high (green). **c**. Bar plots illustrate the representation of MAGs in microbial communities within fermenting cocoa beans (Fermentation; left) and surrounding farm environmental samples (Farm environment, right). Each bar represents a sample group, with percentages indicating reads mapped to MAGs over the total reads generated. **d**. Heatmaps display MAG enrichment profiles during cocoa fermentation progression in Santander, Huila, and Antioquia. Enrichments were determined through generalised linear models, with colours indicating $\log_2$ fold changes derived from each model. Positive fold changes (highlighted in red) indicate enrichments at the respective time point compared to the start of fermentation (0 h), while negative fold changes (highlighted in blue) indicate depletion. Tiles outlined in black denote statistically significant enrichment or depletion ($q < 0.05$) with a $\log_2$ fold change > ± 2. Heatmaps are clustered based on taxonomic classification of the bacteria or fungi.

Supplementary Fig. 5

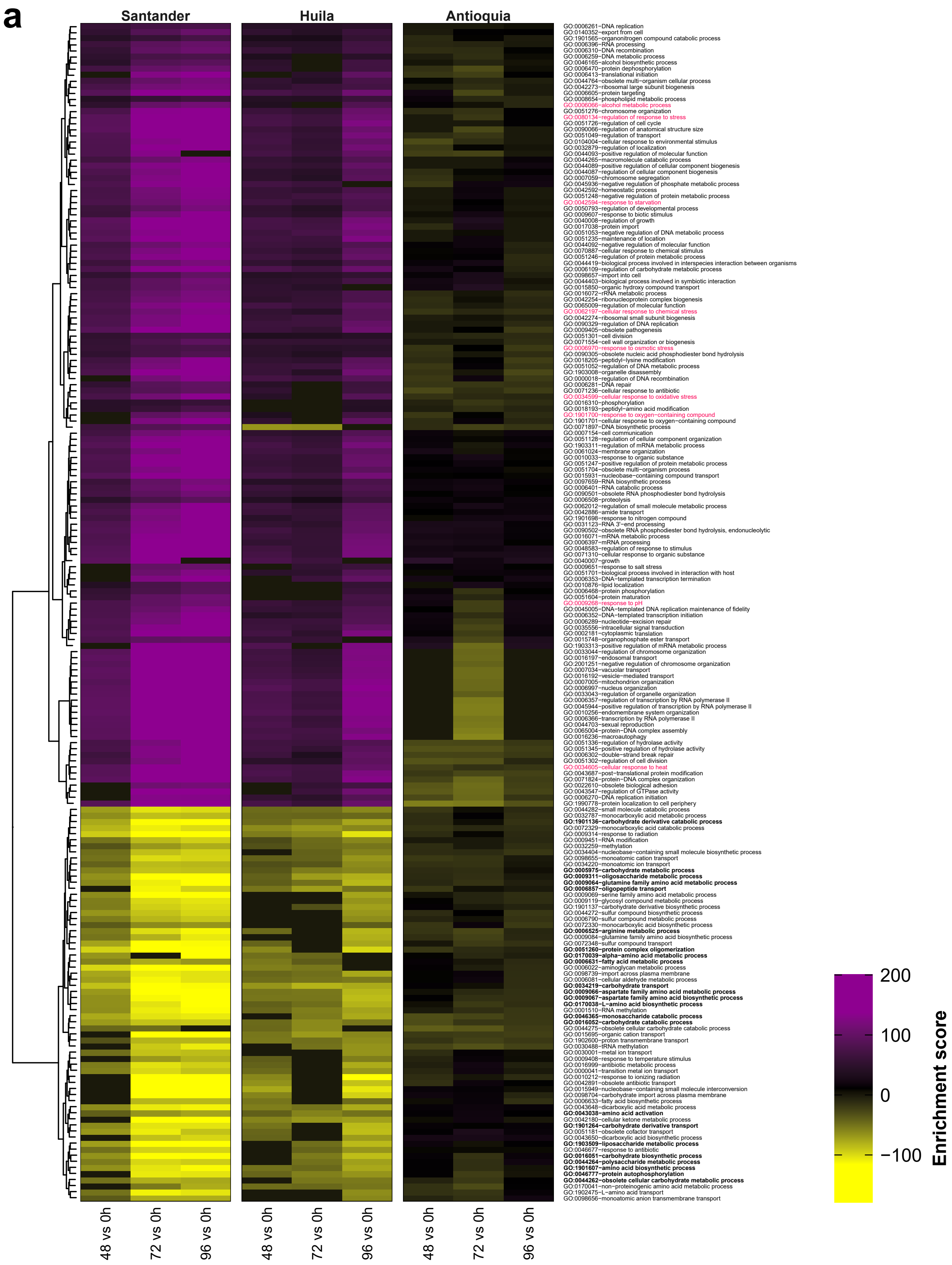

Supplementary Fig. 5. Gene Ontology based enrichment reveals dynamic microbial functional transitions during cocoa bean fermentation. Shotgun metagenomic reads from cocoa bean fermentations were assembled into contigs. Genes encoded on these contigs were predicted and annotated using the eggNOG v5.0.2 database. Relative abundances of microbial genes were estimated by mapping sequencing reads back to the assembled contigs. **a**. Heatmap illustrates biological processes enriched within the microbial communities during fermentation. Gene enrichments were determined using a generalised linear model, followed by enrich Gene Ontology (GO) classifications. Significantly enriched and depleted GO categories were identified based on an adjusted *p*-value < 0.05. The most prominent enriched and depleted GO categories, shared across comparisons, are presented and coloured based on the square root transformed delta rank values (enrichment score) of the GO categories. GO terms enriched (purple) and depleted (yellow) were calculated with respect to the start of the fermentation process (0 h), and clustered according to their enrichment score. Enriched processes typically associated with food fermentation are highlighted in red text, while depleted processes related to microbial primary metabolism are highlighted in bold text.

Supplementary Fig. 6

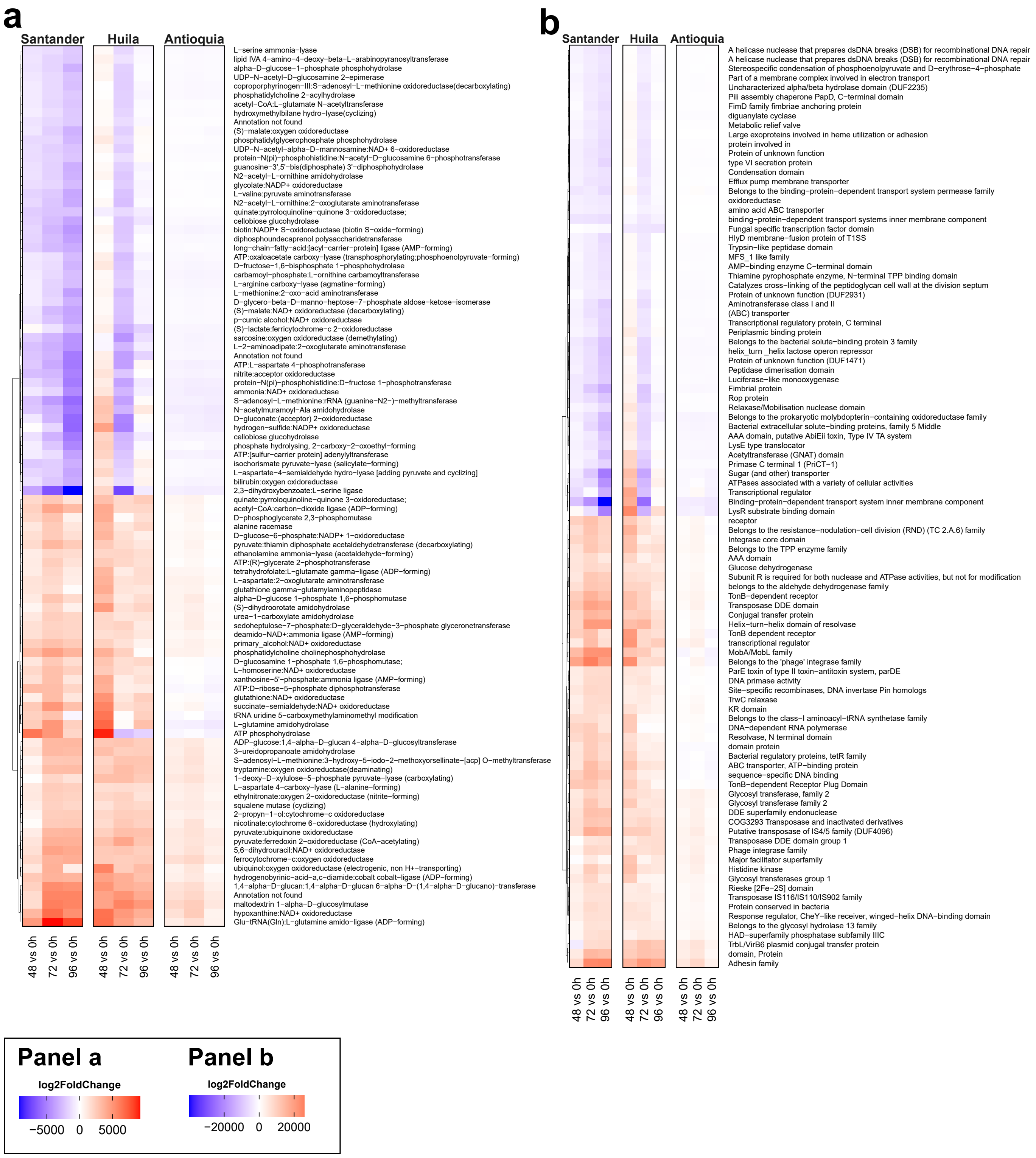

Supplementary Fig. 6. Predicted microbial biochemical reactions and proteins change during cocoa beans fermentation and between fermentations. Shotgun sequencing reads from microbial communities during cocoa fermentation were assembled into contigs. Encoded genes were identified, and predicted proteins were functionally annotated using the eggNOG v5.0.2 database. The relative abundance of contigs throughout fermentation was determined by mapping sequencing reads to the assembled genes, followed by abundance estimation. **a**. Biochemical reactions catalogued in the KEGG (Kyoto Encyclopaedia of Genes and Genomes) database and **b**. proteins enrichment was analysed using a generalized linear model and visualized as heatmaps. Significantly enriched (red) or depletion (blue) biochemical reactions and proteins were identified based on an adjusted *p*-value threshold of < 0.01. Heatmaps are colour-coded according to log_2 fold changes, representing the difference in abundance of each biochemical reaction or protein at specific fermentation time points relative to the start of fermentation. Hierarchical clustering was applied to enrichment profiles to uncover patterns of microbial activity throughout the fermentation process.

Supplementary Fig. 7

a

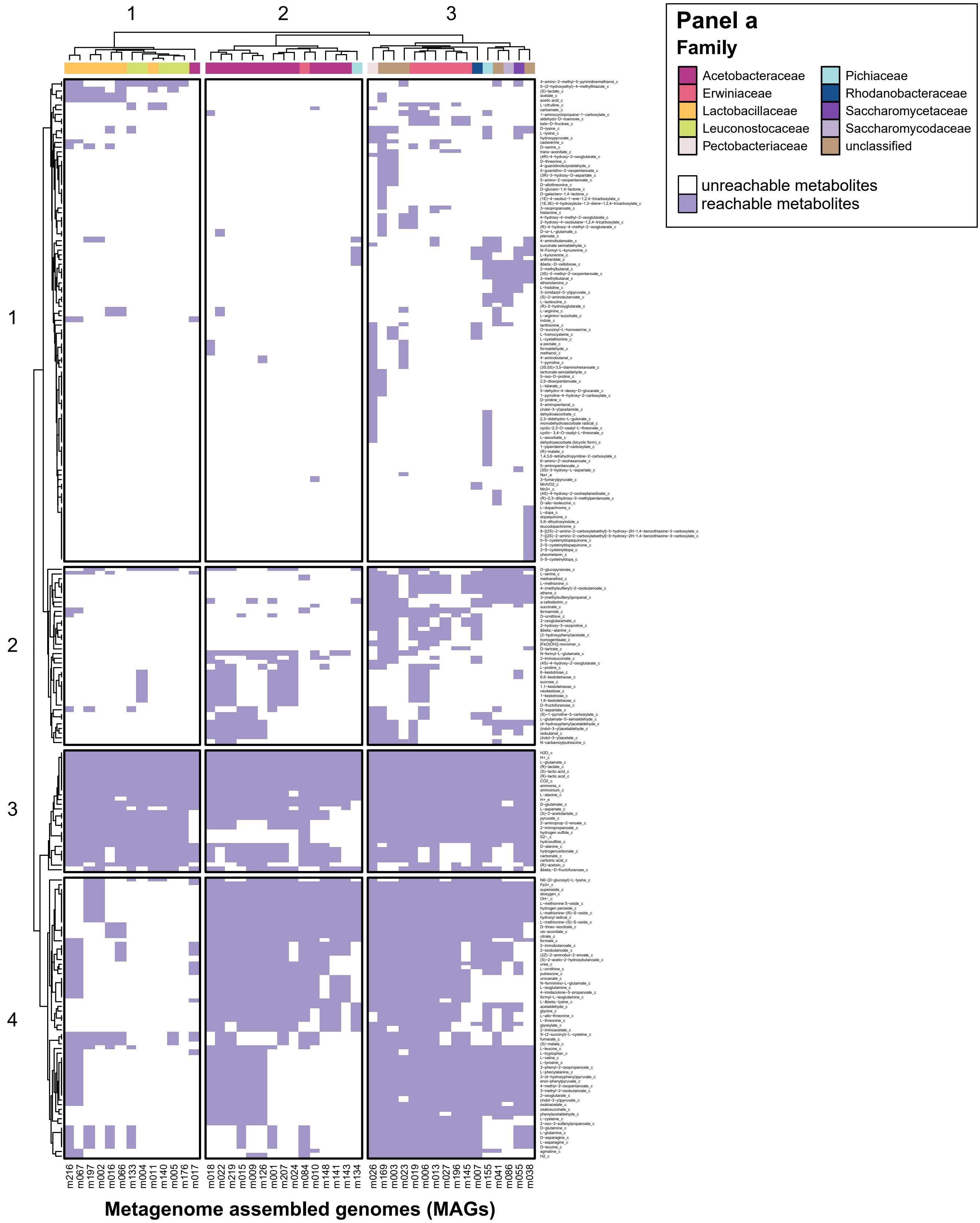

Supplementary Fig. 7. Metabolic capabilities within microbial communities in fermenting cocoa beans are highly redundant. Heatmap illustrates metabolites attainable by each MAG, based on cocoa pulp as the precursor. Genome-scale metabolic networks were reconstructed for the 44 MAGs detected in the fermenting beans, and these reconstructions were used to construct the metabolic network of the fermentation community. Metabolic capabilities of individual MAGs are depicted, and are clustered based on metabolites and MAG metabolic potential, with taxonomic assignments indicated on the top bar.

Supplementary Fig. 8

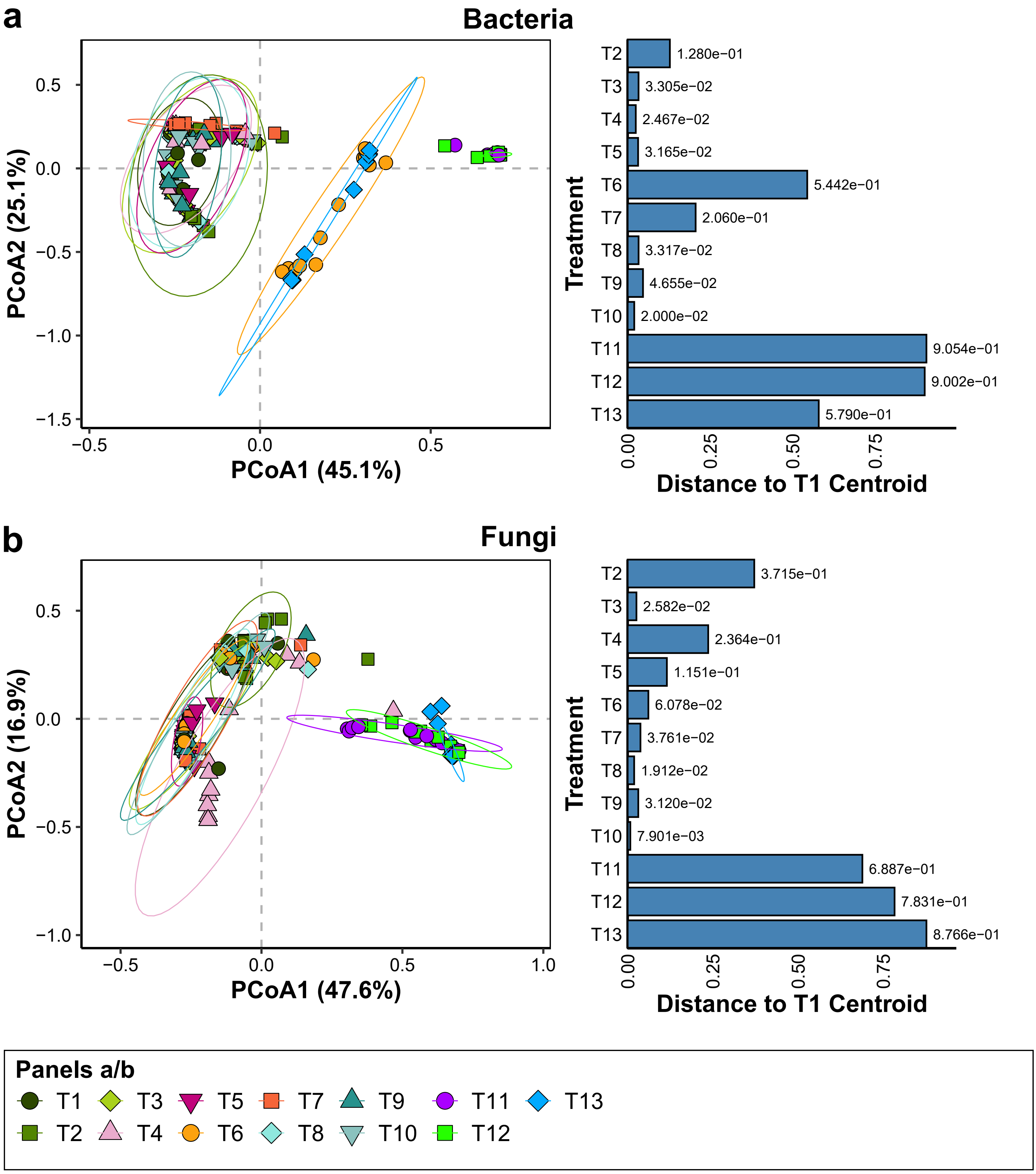

Supplementary Fig. 8. Beta diversity analysis of bacterial and fungal communities highlight treatment-driven shifts in microbial composition during cocoa bean fermentation. Principal Coordinates Analysis (PCoA) plots based on Bray-Curtis dissimilarities illustrate shifts in microbial community composition across cocoa bean fermentation treatments (T1–T13). Panel **a**. shows bacterial communities, while panel **b**. depicts fungal communities. Left panels: Each point represents a biological replicate, with colours and shapes indicating treatment groups. Ellipses denote 95% confidence intervals (*t*-distribution) around each group. Axis labels indicate the proportion of variance explained by each PCoA axis. Right panels: Bar plots show the Euclidean distances between each treatment centroid and the T1 (full synthetic community) centroid, representing the degree of dissimilarity from the baseline condition.

Supplementary Fig. 9

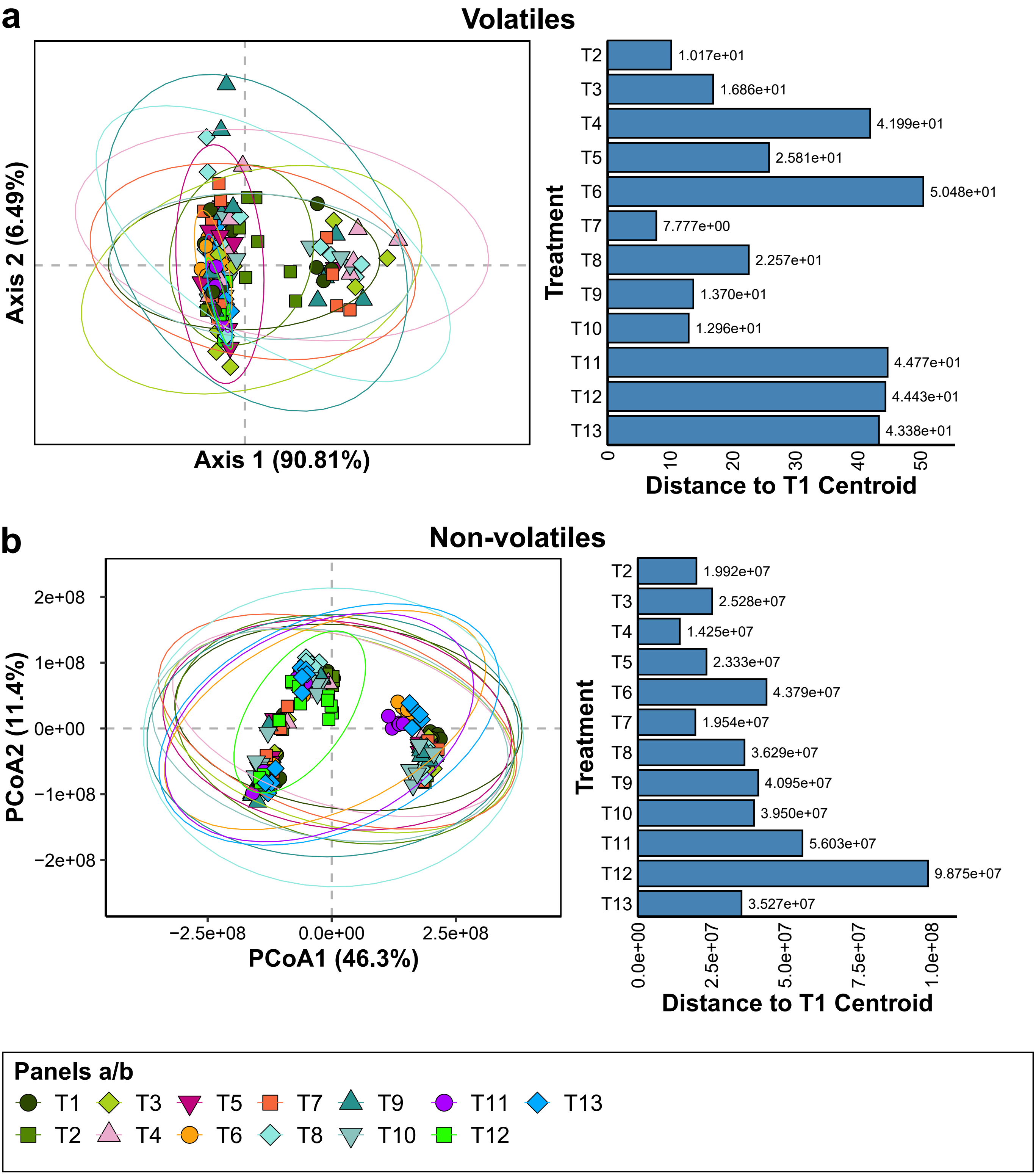

Supplementary Fig. 9. Principal Coordinates Analysis (PCoA) of cocoa bean fermentation metabolic profiles reveals treatment-driven shifts in metabolite composition linked to microbial activity. PCoA plots illustrating changes in metabolite composition across fermentation treatments (T1-T13). **a**. Volatile compounds profiled using Gas Chromatography-Mass Spectrometry (GCMS). b. Non-volatile compounds profiled with Liquid Chromatography-Mass Spectrometry (LCMS). Left panels: Each point represents a biological replicate, coloured and shaped by treatment group. Ellipses indicate 95% confidence intervals (*t*-distribution) around each treatment cluster. Axis labels denote the percentage of total variance explained by each principal coordinate. Right panels: Bar plots show distances of each treatment centroid relative to the full synthetic community (T1), serving as a quantitative measure of dissimilarity from this baseline reference.

Supplementary Results:

Supplementary Result 1. Assigning Oxford Nanopore shotgun metagenome sequences identity

After removing reads that mapped to the cocoa genome (6.94 Gbp, 22.26% of the total DNA output) and human (*Homo sapiens*) genomes (0.07 Gbp, 0.28% of the total DNA output), we retained a total of 7,250,430 high-quality sequence reads (24.15 Gbp) for further analysis. The majority of reads were attributed to bacteria (90.48%) and fungi (9.32%), and a small percentage (0.2%) represented viruses, archaea, and other non-identified sequences. Thus, we focused on bacteria and fungi to understand the microbial dynamics of the cocoa bean fermentative process.

Supplementary Result 2. Assessment of bacterial and fungal enrichment across all fermentation time points

We also analysed the species-level enrichment of bacterial and fungal communities across all fermentation time points relative to time zero. During the first 24 h, only a small proportion of bacterial species were enriched (0.63%) or depleted (1.68%) (Supplementary Fig. 1a). However, at later time points, while the number of enriched bacterial species remained relatively stable (0.44% to 1.2%), the proportion of depleted species increased substantially (7.1% to 10.32%) (Supplementary Fig. 1a). This trend suggests that as fermentation progresses, environmental conditions become increasingly selective, allowing only specific bacterial species to thrive. On the contrary, in the case of fungi, during the first 24 h, we detected an enrichment of 22 fungal species, representing 55% of all fungi identified, while only 20% were depleted (Supplementary Fig. 1d). At later time points, changes in fungal species composition were minimal, with only *Saccharomyces cerevisiae* and *Sugiyamaella lignohabitans* showing further enrichment (Supplementary Fig. 1d). This suggests that fungi rapidly adapt to the fermentation environment,

establishing a relatively stable community early in the process. These enrichment and depletion patterns were further confirmed at higher taxonomic levels (Supplementary Fig. 1). Therefore, we concluded that in the first 48 h, the cocoa bean fermentation might be mainly driven by an enriched population of fungi that include the *Saccharomyces* and *Sugiyamaella* genera. Following this, a second fermentation phase is likely to occur, with the further contribution of enriched bacteria belonging to the *Listeriaceae*, *Lactobacillaceae*, and *Acetobacteraceae* families. These findings align with previous studies on microbial community dynamics in fermenting cocoa beans[1-5]. Supporting this, we found a positive correlation between bacterial and fungal community dissimilarities (Fig. 1g), suggesting potential interkingdom coordination during fermentation.

Supplementary Result 3. Evaluation of genotypic variation of cocoa trees in the identified farms

First, we genotyped the different varieties of cocoa trees found on the farms that produce the beans to be fermented, using single nucleotide polymorphism (SNP) genetic markers (Extended Data Fig. 1b). Our findings revealed that across all three farms, the cocoa varieties observed were genetic hybrids, exhibiting a similar substantial genetic component from the Amelonado and Criollo genetic groups[6], with minor genetic contributions from other cocoa populations (Fig. 2a, Supplementary Fig. 2a-b, and Supplementary Tables 1-2). Thus, we concluded that the cocoa varieties used on the three farms were hybrids with similar genetic backgrounds.

Supplementary Result 4. Kinetics of temperature and pH changes in Huila and Antioquia fermentations

We analysed the kinetics of temperature and pH (testa/pulp and cotyledons) changes in cocoa bean fermentation in the Huila and Antioquia farms, in two consecutive harvests. Our findings showed that in general, the changes in these abiotic fermentation indicators in Huila resembled those observed previously at the Santander farm (Fig. 1a and 2b). Indeed, we found strong correlations between temperature and pH changes recorded in cocoa bean fermentations in both locations (Supplementary Fig. 2c). However, in the case of Antioquia, we observed that the temperature and the cotyledon’s pH dropped after 96 h and 48 h of fermentation, respectively (Fig. 2b) following kinetics that were different from those seen in Santander and Huila fermentations. Indeed, we observed a weak correlation when comparing the

fermentation temperature changes between Antioquia and either Santander or Huila (Supplementary Fig. 2c). This discrepancy was particularly evident in the cotyledon's pH kinetics, with no significant correlation between the fermentation values of Antioquia and the other farms (Supplementary Fig. 2c). It suggests that the fermenting beans from Antioquia might undergo a different chemical transformation compared to the other two farms, likely due to the presence of different microbial populations.

Supplementary Result 5. Kinetics of bacterial and fungal compositional changes in Huila and Antioquia fermentations

Our study revealed that the Huila and Antioquia fermentations presented general microbiota composition characteristics similar to those observed in Santander's fermentation (Fig. 1c-d, 2c-d, and Supplementary Fig. 2d). Supporting our hypothesis however, we found very different dynamics in the microbial community in the Antioquia fermentation, with a high relative abundance of *Lactobacillaceae* and *Acetobacteraceae* at early time points, and *Acetobacteraceae* and *Bacillaceae* by the end of the fermentation (Fig. 2e). In the case of fungi, we only detected *Saccharomycetaceae* at all time points analysed (Fig. 2f). This atypical microbial composition was not detected in the case of Huila, which reproduced microbiota characteristics found in Santander fermentation (Fig. 1e-f, 2e-f). These differences in microbial composition dynamics in Antioquia fermentation compared to the other locations, could explain the differences found in the temperature and pH patterns observed between the fermentations. Moreover, we also noticed that in all cases, the microbiota composition found in the fermentation events varied between the geographical locations a pattern that has also been observed in previous studies[5,7,8] (Fig. 2c-d, and Supplementary Fig. 2d). This geographical signal was stronger in the case of the fungal populations (PERMANOVA R^2 = 0.09946, p < 0.0002) in comparison to the bacteria (PERMANOVA R^2 = 0.06982, p = 0.0053), suggesting that the local environment or origin of the sources of microbes for fermentation could also be affecting the cocoa bean fermentation properties (Fig. 2c-d and Supplementary Fig. 2d).

Supplementary Result 6. Studying environmental sources of microbes for fermentation

We explored whether the differences in microbial composition and dynamics observed across the three locations could be explained by the environmental sources of microbes for fermentation. We collected samples from potential environmental sources of microbes for fermentation on all farms, such as the cocoa

tree leaves, cocoa pod surface, the hands of the farmers handling the beans, the surface of the tools the farmer used to open the cocoa pods, flies in contact with the cocoa beans, and the inner surface of the empty fermentation boxes. Employing whole metagenome shotgun sequencing, we characterised the bacterial and fungal populations within these environmental samples and compared them to those in the fermenting beans. Our observations indicated that, generally, all environmental samples had a higher bacterial alpha diversity compared to the different time points analysed during fermentation (Supplementary Fig. 2e). Nevertheless, the bacterial alpha diversity was notably reduced to levels similar to those found in the cocoa bean fermentations in samples in direct contact with the fermenting beans, such as flies and the inner surfaces of the fermentation boxes. Furthermore, a principal coordinates analysis separated most of the environmental samples from the fermentation samples along the second axis, with the exception of fermentation box samples, which grouped with the late fermentation time points (Supplementary Fig. 2e). Therefore, we speculated that these environmental sources may contribute to the composition of the microbial communities present in the cocoa bean fermentations. Indeed, we observed that the bacterial composition found on the inner surfaces of the boxes resembles the composition of later time points in the fermentations (Supplementary Fig. 2e and 2g). To further explore these connections, we performed community-wide microbial source tracking analysis to ascertain how the environmental sources contributed to the fermentation communities (Supplementary Fig. 2i). Interestingly, the analysis showed that all the environmental samples examined (soil, leaf, pod, hand, tool, fly, and fermentation box) contributed to the fermentation microbiota, albeit in varying proportions and at different times. In Santander and Huila, bacterial communities at 0 h predominantly originated from the farmers' hands, while at 96 h, they were mainly traced back to the fermentation box (Supplementary Fig. 2i). Conversely, in Antioquia, the bacterial community mainly stemmed from the fermentation box at both 0 h and 96 h. These findings strongly suggest that bacteria harboured within the box surfaces might function as a ‘memory’, serving as stable inoculation sources over time and contributing to subsequent rounds of bean fermentation. This result was robust across all farms analysed (Supplementary Fig. 2e, 2g, and 2i).

In the case of fungal populations, all environmental samples showed comparable alpha diversity to the early time points of the bean fermentations (Supplementary Fig. 2f). With the exception of the surface of the cocoa pod and the cocoa leaves that grouped together, the rest of the environmental samples were

interspersed with the early time points of the bean fermentations (Supplementary Fig. 2f and 2h) showing similar fungal community compositions. Additionally, the source contributions for fungal communities displayed variability across the three farms at both time points (Supplementary Fig. 2i). These results suggest that the fungal component of the initial sources of microbes for fermentation likely originates from multiple environmental sources. This finding could explain the strong geographic signal observed in the fungal populations from the analysed fermentations (Fig. 2d). Moreover, community-wide microbial source tracking analysis highlighted that the fermentation box communities were initially derived from various environmental origins, primarily soil, cocoa leaves, hands, and flies, with differing source contributions between farms for both bacterial and fungal communities (Supplementary Fig. 2i). These divergent contributions from environmental sources among farms, coupled with temporal disparities, add an additional layer of complexity that potentially enriches the diversity of flavours observed across different locations.

Supplementary Result 7. Assessment of fermentation quality using classical quality parameters

We evaluated whether changes in the identified fermentation signatures (temperature, pH, and microbial composition) could offer insights into the quality of the fermented and dried cocoa beans. We evaluated nine quality parameters[9-11] commonly used in the chocolate industry, to rapidly assess the commercial quality of the fermented and dried cocoa beans (Supplementary Fig. 2j). Bean cotyledons exhibiting a brown hue (a sign of complete fermentation) without any flaws, or cotyledons displaying a brown/partially purple colour, post-fermentation and drying, are recognised as fermented beans of superior quality[9,11]. While the fermentation degree of the beans from the second harvest was higher compared to the first harvest in all farms (Fisher's Exact Test p-value < 0.05), we found no significant differences (Fisher's Exact Test p-value $= 0.9045303$) in the fermentation degree quality parameters between the different locations, despite the fact that the Antioquia fermentation signatures were very atypical (Fig. 2b-f). Notably, the criteria which are mainly used to assess the degree of fermentation of the beans, specifically, the quantity of beans displaying purple/violet (indicating under-fermentation), grey slate (indicating over-fermentation), partially purple, and fully fermented with no defects[9,11], were comparable across the three fermentation locations (Supplementary Fig. 2j). These results suggest that the fermentation markers identified here (temperature, pH, and microbial composition variations), were not informative about the overall degree of

fermentation of the beans. Therefore, we postulated that variations in these fermentation signatures, which are associated with chemical processes taking place within the beans, could offer insights into the flavour characteristics of the chocolate.

Supplementary Result 8. Comparing chocolate flavour attributes in the three farms selected

A canonical analysis of the principal coordinate (CAP) using flavour attributes of all fermentations, separated the liquors from Santander and Huila from those from Antioquia on the first coordinate axis (Fig. 3a). In line with the abiotic and biotic fermentation signature changes (Fig. 1 and 2), this result indicated that the Santander and Huila fermentations shared core and complementary flavour attributes, and that both were different from those detected in samples from Antioquia (Fig. 3a). Further, we noticed a similarity in the organoleptic qualities between the liquors derived from the fermented beans in Santander and Huila, and a fine flavour liquor from Madagascar (Fig. 3b). In contrast, the liquor from Antioquia clustered with reference bulk liquors from Ivory Coast and Ghana, displaying a limited range of flavour notes (Fig. 3b). These results underscore the pivotal role of bean fermentation, especially the dynamics of its abiotic and biotic markers defined here, as a critical factor governing the development of fine flavour in chocolate production.

Supplementary Result 9. Addressing duplications in the abiotic features identified in fermentations

To refine our analysis, we addressed potential duplications in the identified abiotic features related to temperature and cotyledon pH by clustering a pairwise correlation matrix. This approach identified clusters of highly correlated features (Extended Data Fig. 2a-b). We selected only the features with the highest coefficient of variation within each cluster for further analysis. Among the identified kinetic features, those related to exponential phases and inflection in temperature kinetics, as well as those associated with the time needed to achieve maximum kinetics and exponential phases in cotyledon pH, exhibited the highest coefficient of variation during the fermentations across the different locations (Extended Data Fig. 2a-b). These selected features were used to assess the importance of the abiotic parameters in predicting flavour attributes in the final chocolate.

Supplementary Result 10. Validation of the predictive power of the identified abiotic and biotic markers

To further validate the robustness and predictive power of these identified markers, we analysed associations between the abiotic and microbial features and the chocolate flavour attributes across 19 and 11 independent natural fermentations, respectively. These fermentations were conducted over multiple years across diverse agroecological cocoa-growing regions in Trinidad, a country renowned for producing high-quality fine or flavour cocoa beans. Farms and fermentation events were selected to capture a broad spectrum of cocoa flavour profiles. Despite this broad panel in sensory attributes, derived from fermentations spanning multiple years, varied agroecological regions, and a different country, our analysis revealed strong associations between several features and key flavour attributes, consistent with those identified in Colombia (Supplementary Fig. 3, Supplementary Table 3). For instance, the abiotic marker, time to pH maximum kinetic energy, was linked to dark wood notes, while the duration of pH exponential decay phase was associated with dried fruit, bitterness, and cocoa flavours (Fig. 3c, Supplementary Fig. 3, Supplementary Table 3). Among microbial features, the area under the curve of *Saccharomyces* was linked with acetic, tropical, citrus, and overripe fruit flavours, whereas *Komagataeibacter* was tied to orange blossom, grassy/green vegetal/herbal, nut flesh, and fruit attributes (Fig. 3d, Supplementary Fig. 3, Supplementary Table 3). Similarly, the area under the curve and initial density of *Gluconobacter* and *Pasteurella*, respectively, were connected to berry notes, while the inflection points of *Torulaspora* and *Pasteurella* were associated with brown fruit flavours (Fig. 3d, Supplementary Fig. 3, Supplementary Table 3). Moreover, several features detected in Colombian fermentations showed even stronger associations in the Trinidad fermentations. For example, the time to pH maximum kinetic energy was linked to acetic, cocoa, and citrus notes, the rate of temperature change during the exponential phase was associated with berry, light wood, overripe fruit, caramel/panela, and floral notes, and the pH rate of change during the exponential phase was tied to lactic acid and dirty/dusty attributes (Fig. 3c, Supplementary Fig. 3, Supplementary Table 3). Additionally, the area under the curve for *Gluconobacter*, *Acetobacter*, *Pasteurella*, *Komagataeibacter*, and *Bacillus* connected with floral, yellow/orange/white flesh, savoury/umami, light wood, and dried fruit notes, respectively, while the initial density and midpoint of *Bacillus* were linked to cocoa, citrus, tropical, and acetic attributes (Fig. 3d, Supplementary Fig. 3, Supplementary Table 3). Overall, the results indicate that together with the abiotic features associated

with temperature and pH in the fermentation, it is possible to influence the chocolate flavour by manipulating the microbial community composition and dynamics during the cocoa fermentation.

Supplementary Result 11. Validation of the metagenome assembled genomes (MAGs) quality

We examined the impact of increasing MAG genome completeness thresholds, from >10% to >90%, on metabolic network features (Supplementary Fig. 4a). This analysis revealed a clear trade-off: higher completeness thresholds reduced the number of retained MAGs and, consequently, the metabolic reactions and compounds represented in the network. To balance MAG genome completeness with metabolic coverage, we selected thresholds of ≥50% for bacterial MAGs and ≥30% for fungal MAGs. These criteria were based on (i) preserving metabolic diversity in the network analysis and (ii) accounting for generally lower completeness of fungal MAGs, which nonetheless contribute unique and critical metabolic functions to the fermentation. After removing duplicates, MAGs with contamination >10%, and those below the completeness thresholds, 55 MAGs remained for further analysis (Supplementary Fig. 4a-b and Supplementary Table 4). Genome completeness for these MAGs ranged from 50% to 98.65% for bacteria and from 31.11% to 44.38% for fungi (Supplementary Fig. 4a-b and Supplementary Table 4). We confirmed that these MAGs successfully replicated the microbial community compositional dynamics of the fermentations, with 61.34% to 88.66% of all shotgun read data from the fermentation samples from our study, mapping to the MAGs (Supplementary Fig. 4c). In contrast, when analysing the sequence data from the environmental samples, a lower average mapping percentage (19.27%) was achieved, which further reinforced that the MAGs represented the community composition patterns specific to the cocoa fermentations (Supplementary Fig. 4c). Indeed, the taxonomic assignment of these MAGs identified main fermentation associated phyla, such as Ascomycota, Bacteroidetes, Firmicutes and Proteobacteria (Fig. 4a). Moreover, employing a linear model, we determined that the relative abundance of the MAGs mirrored the typical kinetic changes in microbial composition observed in Santander and Huila fermentations with an enrichment in *Acetobacteraceae* and *Saccharomycetaceae* after 48 h of fermentations, as well as the atypical community pattern discovered in Antioquia fermentation (Supplementary Fig. 4d).

Next, we identified the microbial biological processes that were enriched within the microbial communities during the fermentation on the three farms. For this, we analysed the abundance of the bacterial and fungal

genes within the communities across the samples using a generalised linear model and subsequently identified the enriched Gene Ontology (GO) categories. Aligning with the microbial community composition patterns (Supplementary Fig. 4d), this functional analysis not only affirmed similarities in the dynamics of function enrichment in the Santander and Huila fermentations, but also highlighted stark differences in comparison to the Antioquia fermentation (Supplementary Fig. 5, Supplementary Table 5). Delving into the analysis of the most prominent enriched GO categories not only uncovered functional categories linked to microbial division and growth, but also revealed enrichment in microbial biological processes typically associated with food fermentation, such as pH response, alcohol biosynthetic process, alcohol metabolic process, response to heat, starvation and osmotic and oxidative stresses[12-14] in fermentations from Santander and Huila (Supplementary Fig. 5, Supplementary Table 5). As expected, these functions became more enriched as the fermentations progressed (Supplementary Fig. 5, Supplementary Table 5). Additionally, enrichment analysis of predicted metabolic pathways and proteins further highlighted key enzymatic groups that were enriched during the fermentation, that likely contributed to the flavour development in the cocoa beans (Supplementary Fig. 6, Supplementary Table 6-7). For instance, enzymes involved in amino acid metabolism, such as aminotransferase, which produce precursors that can lead to the formation of pyrazines, which are known aroma associated compounds in chocolate that impart roasted and nutty notes, were notably abundant[15,16] (Supplementary Fig. 6). Additionally, L-aspartate 4-carboxy-lyase, which breaks down L-aspartate into alanine and other intermediates that generate sweet and savoury flavour precursors[17,18], showed an enriched profile. Similarly, enzymes such as ethanolamine ammonia-lyase, which catalyses acetaldehyde production, a volatile compound imparting fruity and sometimes pungent aromas, was prominent, alongside primary alcohol:NAD+ oxidoreductase, an enzyme involved in aldehyde synthesis, contributing to fruity, floral, and fatty aromas[19-21] (Supplementary Fig. 6). Notably, these enriched biological processes, metabolic pathways, and proteins were observed in the Santander and Huila fermentations but limited in the Antioquia samples, underscoring the distinct nature of this fermentation (Supplementary Fig. 5-6). Moreover, consistent with estimated nutrient transformation kinetics in the bean pulp and cotyledons, we observed a pronounced repression of microbial processes related to primary metabolism across the fermentations (Supplementary Fig. 5-6). Taken together, these findings suggest that the sequenced microbial communities from the fermentations and the corresponding assembled MAGs captured the functional and compositional dynamics of the microbiota present in the

fermentations. We hypothesised that the constructed MAGs likely capture essential metabolic pathways for cocoa bean fermentation and the development of diverse chocolate flavour attributes.

Supplementary Result 12. Identifying a defined number of microbes with full metabolic capabilities required for cocoa fermentations

Taken together, our overall results related to metabolic capabilities of the constructed MAGs indicated that it is feasible to identify a defined number of microbes that have the full diversity of metabolic capabilities required for driving cocoa fermentations, and that these microbes can be used as starters to modulate the final flavour attributes of chocolate. Indeed, our metabolic network analysis pinpointed 10 MAGs with metabolic capacities equivalent to the entire microbial community identified in the cocoa fermentations (Fig. 4b-c). The metabolic network analysis indicated that this reduced community had the capacity to produce 97.5% (n = 237) of the metabolites achievable by the full microbial community (Fig. 4c). Furthermore, the analysis revealed that while individual microbes within the defined community could reach 216 metabolites, an additional 21 metabolites were attained through cooperative interactions among community members in cross-feeding reactions.

Supplementary Result 13. Building a collection of bacterial and fungal isolates

We isolated bacterial and fungal strains from fermenting cocoa beans at the Cocoa Research Centre fermentation facility in Trinidad and Tobago. The fermenting bean samples were inoculated on different agar-based selection media for the culture of the main taxonomic groups present in fermenting cocoa beans[2,22-24]. Using this protocol, we isolated 368 and 115 bacterial and fungal strains, respectively. The taxonomy of the microbial isolates was assigned using Sanger sequencing of the bacterial 16S ribosomal RNA (*16S rRNA*) gene in the case of bacteria, and the internal transcribed spacer (ITS) region in the case of fungi. We used the taxonomy of the strains and their morphological and colour characteristics to eliminate duplicate isolates, resulting in a refined collection comprising 57 bacterial and 14 fungal strains (Extended Data Fig. 3b-c, Supplementary Table 8). This collection encompasses a diverse representation of bacterial and fungal families commonly found in cocoa fermentation[2,4,5,22,25] (Extended Data Fig. 3b-c). Furthermore, the bacterial and fungal families represented in our collection, collectively accounted for approximately 54.2% to 98.1% of bacteria, and 76.7% to 99.7% of fungi, of the total relative abundance

observed in cocoa fermentation samples at different time points, from the Colombian fermentations studied (Extended Data Fig. 3d).

Supplementary Result 14. Design and validation of the defined microbial consortium

We selected from our collection, a subset of 5 bacterial, and 4 fungal strains (Supplementary Table 8), that approximate the taxonomy and their metabolic potential of the 10 MAGs identified in our metabolic network analysis accounting for approximately 55.6% of the taxa at the Family level (Fig. 4b, 4d). To confirm their metabolic capacity were similar to the 10 MAGs, we sequenced the complete genome of these 9 microbial isolates and analysed their metabolic potential to utilise the precursors of cocoa pulp (Fig. 4b-c). This analysis verified that the consortium of 9 isolates had the capacity to produce 94.9% of the metabolites attainable by the 10 MAGs, and 92.6% of the metabolites attainable by the full microbial community, when initiated with cocoa pulp as the precursor (Fig. 4c). Therefore, we used this microbial consortium to evaluate the predictive accuracy of our methodology in designing starter cultures capable of reproducing cocoa fermentations with desirable flavour attributes. We demonstrated the lack of redundancy in metabolic attributes present in the selected microbial consortium performing independent dropout experiments in which we removed individual strains from the consortium, one at a time, and inoculated beans with the resulting microbial combinations (Extended Data Fig. 4a). As controls, we used uninoculated beans and beans inoculated with a randomly selected 9-member microbial consortium (Extended Data Fig. 4a). We observed that inoculating cocoa beans with all the synthetic microbial communities (full, drop-outs, and random) in microboxes under controlled conditions, in general reproduced the kinetics of changes in the pH of the testa/pulp and cotyledons found in the fermentations of the Santander and Huila farms with flavour characteristics of fine chocolate (Fig. 1a, 2b, and Extended Data Fig. 4b, 5a). We confirmed that these changes in fermentation pH were absent in the non-inoculated beans that we used as a control (Extended Data Fig. 4b, 5a).

Further, using the full 9-members defined starter we reproduced general microbiota characteristics found in the Santander and Huila fermentations (Fig. 1c-1f, 2c-2f, 5a-d). Canonical analysis of principal coordinates (CAP) showed significant differences in microbial community composition between fermentations inoculated with the synthetic community and those that were not (Fig. 5c-d). Similar to the

farm fermentations (Fig. 1c-1f, and 2c-2f), we observed a dynamic shift in microbial composition, characterised by an enrichment of *Acetobacteraceae*, *Saccharomycetaceae*, and *Saccharomycodaceae* at later time points in the fermentations (Fig. 5c-5f). We observed that the total amount of microbial cells is not stable during cocoa bean fermentations and increases with time reaching values close to $1.5x10^9$ cells/mL of fermented pulp at the end of the fermentation process (Extended Data Fig. 5c). However, in the dropout experiments, removing individual members of the microbial inoculum eliminated the typical decrease in bacterial and fungal alpha diversity in five of the bacterial dropout combinations and one of the fungal dropout combinations (Extended Data Figure 6a-b). This suggests a key role of most individual consortium components for maintaining microbial community structure. Analysis of between samples variation (beta diversity) confirmed differences in microbial community compositions across cocoa beans fermentations driven by the different dropout microbial combinations that were mostly explained by the variables 'composition of the initial starters', 'time', and the interaction between them (Extended Data Fig. 7, Supplementary Fig. 8). We also confirmed that the microbiota characteristics of the uninoculated beans and beans inoculated with the random selected synthetic community were different from the full synthetic community (Extended Data Fig. 4, 6 and 7, Supplementary Fig. 8). With the analysis of our new dropout experiments (analysis of 13 different fermentations inoculated with distinct microbial combinations resulting from removing one by one all the inoculum members) we can conclude that: a) Changes in the inoculum composition cause changes in the flavour characteristics, b) The microbial composition is the causal reason of changes in the flavour attributes, c) This microbial-driven changes in the flavour is likely caused by metabolic transformation of the beans during fermentation. Thus, our results indicate that it is possible to manipulate the microbial community during cocoa fermentations to alter the chocolate flavour in favour of more desired profiles. Overall, we demonstrated that it is feasible to reproduce the main abiotic and biotic characteristics of cocoa fermentation found in spontaneous fermentation on the farm, using controlled conditions and the design of defined synthetic starters.

Supplementary Result 15. Addressing the relationship between the metabolic profile of fermentation and the composition of the microbial starter

We confirmed that changes in the metabolic profiles of VOCs were linked to the initial microbial composition of the starters and evolved over time during fermentation (Extended Data Fig. 8a-b,

Supplementary Fig. 9a). Kinetic analysis of VOC accumulation in fermentations driven by the full, dropout, and randomly selected synthetic communities revealed significant differences in the concentration of individual VOCs across the 13 synthetic starters and time points analysed (Extended Data Fig. 8a–d). The accumulation patterns of VOCs in these fermentations differed from those in non-inoculated fermentations, where VOC profiles remained largely unchanged compared to time zero (Extended Data Fig. 8c). We extended our metabolic analysis to non-volatile compounds detected in cocoa beans during fermentation and observed clear differences in metabolic profiles across the 13 synthetic starters (full, dropout, and randomly selected communities), which became more pronounced as fermentation progressed (Extended Data Fig. 8e-f, Supplementary Fig. 9b). For example, at late time points, principal coordinate analysis showed that non-inoculated fermentations and those inoculated with a randomly selected synthetic community were metabolically distinct from the other controlled fermentations (Extended Data Fig. 8e-f, Supplementary Fig. 9b). At the level of individual compounds, hundreds to thousands exhibited distinct accumulation kinetics across all fermentations analysed (Extended Data Fig. 9a-c). We verified that the accumulation kinetics of the different metabolites were different in all fermentations (dropout, random, and non-inoculated) compared to the fermentation inoculated with the full synthetic community, confirming the lack of redundancy in the metabolic capacities of the defined starter designed in this work (Extended Data Fig. 9b-c).

Supplementary Tables

Supplementary Table 1. *Theobroma cacao* SNP panel used to genotype cacao trees sampled from cocoa plantations located in Santander, Huila, and Antioquia regions in Colombia.

Supplementary Table 2. SNP profiles of *Theobroma cacao* trees assessed from cocoa plantations in this study.

Supplementary Table 3. Feature importance of abiotic and biotic markers associated with flavour attributes in chocolate from Colombia and Trinidad cocoa bean fermentations.

Supplementary Table 4. Genome and quality statistics of metagenome assembled genomes (MAGs) constructed from metagenomic datasets originating from cocoa plantations in Santander, Huila, and Antioquia.

Supplementary Table 5. Enriched GO categories across microbial communities during cocoa bean fermentation.

Supplementary Table 6. Enriched KEGG reactions across microbial communities during cocoa bean fermentation.

Supplementary Table 7. Enriched proteins across microbial communities during cocoa bean fermentation.

Supplementary Table 8. Characterization of bacterial and fungal isolates using 16S rRNA and ITS amplicon sanger sequencing.

References

1 Ghisolfi, R. *et al.* Bacterial and fungal communities are specifically modulated by the cocoa bean fermentation method. *Foods* **12**, 2024 (2023).

2 Papalexandratou, Z. *et al.* Linking cocoa varietals and microbial diversity of Nicaraguan fine cocoa bean fermentations and their impact on final cocoa quality appreciation. *Int. J. Food Microbiol.* **304**, 106-118 (2019).

3 Papalexandratou, Z. *et al.* Species diversity, community dynamics, and metabolite kinetics of the microbiota associated with traditional Ecuadorian spontaneous cocoa bean fermentations. *Appl. Environ. Microbiol.* **77**, 7698-7714 (2011).

4 Illeghems, K., De Vuyst, L., Papalexandratou, Z. & Weckx, S. Phylogenetic analysis of a spontaneous cocoa bean fermentation metagenome reveals new insights into its bacterial and fungal community diversity. *PLoS ONE* **7**, e38040 (2012).

5 Pacheco-Montealegre, M. E., Dávila-Mora, L. L., Botero-Rute, L. M., Reyes, A. & Caro-Quintero, A. Fine resolution analysis of microbial communities provides insights into the variability of cocoa bean fermentation. *Front. Microbiol.* **11**, doi:10.3389/fmicb.2020.00650 (2020).

6 Motamayor, J. C. *et al.* Geographic and genetic population differentiation of the Amazonian chocolate tree (*Theobroma cacao* L). *PLoS ONE* **3**, e3311 (2008).

7 Bortolini, C., Patrone, V., Puglisi, E. & Morelli, L. Detailed analyses of the bacterial populations in processed cocoa beans of different geographic origin, subject to varied fermentation conditions. *Int. J. Food Microbiol.* **236**, 98-106 (2016).

8 Papalexandratou, Z., Camu, N., Falony, G. & De Vuyst, L. Comparison of the bacterial species diversity of spontaneous cocoa bean fermentations carried out at selected farms in Ivory Coast and Brazil. *Food Microbiol.* **28**, 964-973 (2011).

9 Sukha, D. A. & Ali, N. A. Analysing sensory and processing quality of cocoa. In *Achieving sustainable cultivation of cocoa – genetics, breeding, cultivation and quality* Vol. 1 (ed Pathmanathan Umaharan) Ch. 27, 27, 395 – 436 (Burleigh Dodds Science Publishing, 2018).

10 Ilangantileke, S. G., Wahyudi, T. & Bailon, M. A. G. Assessment methodology to predict quality of cocoa beans for export. *J. Food Qual.* **14**, 481-496 (1991).

11 Sukha, D. A. The grading and quality of dried cocoa beans. In *Drying and roasting of cocoa and coffee* (eds Ching Lik Hii & Flávio Meira Borém) Ch. 5, 5, 89-139 (CRC Press, 2019).

12 Giraffa, G. Studying the dynamics of microbial populations during food fermentation. *FEMS Microbiol. Rev.* **28**, 251-260 (2004).

13 Vinicius De Melo Pereira, G. *et al.* A review of selection criteria for starter culture development in the food fermentation industry. *Food Rev. Int.* **36**, 135-167 (2020).

14 Sharma, R., Garg, P., Kumar, P., Bhatia, S. K. & Kulshrestha, S. Microbial fermentation and its role in quality improvement of fermented foods. *Fermentation* **6**, 106 (2020).

15 Schnermann, P. & Schieberle, P. Evaluation of key odorants in milk chocolate and cocoa mass by aroma extract dilution analyses. *J. Agric. Food Chem.* **45**, 867-872 (1997).

16 Counet, C., Callemien, D., Ouwerx, C. & Collin, S. Use of gas chromatography− olfactometry to identify key odorant compounds in dark chocolate. Comparison of samples before and after conching. *J. Agric. Food Chem.* **50**, 2385-2391 (2002).

17 Bachmanov, A. A. *et al.* Genetics of amino acid taste and appetite. *Advances in Nutrition* **7**, 806S-822S (2016).

18 Yaylayan, V. A. & Keyhani, A. Origin of 2, 3-pentanedione and 2, 3-butanedione in D-glucose/L-alanine Maillard model systems. *J. Agric. Food Chem.* **47**, 3280-3284 (1999).

19 Rodriguez-Campos, J., Escalona-Buendía, H. B., Orozco-Avila, I., Lugo-Cervantes, E. & Jaramillo-Flores, M. E. Dynamics of volatile and non-volatile compounds in cocoa (*Theobroma cacao* L.) during fermentation and drying processes using principal components analysis. *Food Res. Int.* **44**, 250-258 (2011).

20 Rodriguez-Campos, J. *et al.* Effect of fermentation time and drying temperature on volatile compounds in cocoa. *Food Chemistry* **132**, 277-288 (2012).

21 Aprotosoaie, A. C., Luca, S. V. & Miron, A. Flavor chemistry of cocoa and cocoa products—an overview. *Compr. Rev. Food Sci. F.* **15**, 73-91 (2016).

22 Lefeber, T., Gobert, W., Vrancken, G., Camu, N. & De Vuyst, L. Dynamics and species diversity of communities of lactic acid bacteria and acetic acid bacteria during spontaneous cocoa bean fermentation in vessels. *Food Microbiol.* **28**, 457-464 (2011).

23 Crafack, M. *et al.* Influencing cocoa flavour using *Pichia kluyveri* and *Kluyveromyces marxianus* in a defined mixed starter culture for cocoa fermentation. *Int. J. Food Microbiol.* **167**, 103-116 (2013).

24 Moreira, I. M. d. V., Miguel, M. G. d. C. P., Duarte, W. F., Dias, D. R. & Schwan, R. F. Microbial succession and the dynamics of metabolites and sugars during the fermentation of three different cocoa (*Theobroma cacao* L.) hybrids. *Food Res. Int.* **54**, 9-17 (2013).

25 Garcia-Armisen, T. *et al.* Diversity of the total bacterial community associated with Ghanaian and Brazilian cocoa bean fermentation samples as revealed by a 16 S rRNA gene clone library. *Appl. Microbiol. Biotechnol.* **87**, 2281-2292 (2010).
