# Supplementary material for: A defined microbial community reproduces attributes of fine flavour chocolate fermentation
Source: Nat Microbiol. 2025 Aug 18;10(9):2130–52. doi: 10.1038/s41564-025-02077-6 (PMC12408344; doi:10.1038/s41564-025-02077-6)
Supplement: Supplementary file 2 — Reporting Summary [file 41564_2025_2077_MOESM2_ESM.pdf]

nature portfolio

Corresponding author(s): David E. Salt and Gabriel Castrillo

Last updated by author(s): Jun 14, 2025

Reporting Summary

Nature Portfolio wishes to improve the reproducibility of the work that we publish. This form provides structure for consistency and transparency in reporting. For further information on Nature Portfolio policies, see our Editorial Policies and the Editorial Policy Checklist.

Statistics

For all statistical analyses, confirm that the following items are present in the figure legend, table legend, main text, or Methods section.

n/a	Confirmed	
☐	☒	The exact sample size (*n*) for each experimental group/condition, given as a discrete number and unit of measurement
☐	☒	A statement on whether measurements were taken from distinct samples or whether the same sample was measured repeatedly
☐	☒	The statistical test(s) used AND whether they are one- or two-sided<br>*Only common tests should be described solely by name; describe more complex techniques in the Methods section.*
☐	☒	A description of all covariates tested
☐	☒	A description of any assumptions or corrections, such as tests of normality and adjustment for multiple comparisons
☐	☒	A full description of the statistical parameters including central tendency (e.g. means) or other basic estimates (e.g. regression coefficient) AND variation (e.g. standard deviation) or associated estimates of uncertainty (e.g. confidence intervals)
☐	☒	For null hypothesis testing, the test statistic (e.g. *F*, *t*, *r*) with confidence intervals, effect sizes, degrees of freedom and *P* value noted<br>*Give P values as exact values whenever suitable.*
☐	☒	For Bayesian analysis, information on the choice of priors and Markov chain Monte Carlo settings
☒	☐	For hierarchical and complex designs, identification of the appropriate level for tests and full reporting of outcomes
☐	☒	Estimates of effect sizes (e.g. Cohen's *d*, Pearson's *r*), indicating how they were calculated

Our web collection on statistics for biologists contains articles on many of the points above.

Software and code

Policy information about availability of computer code

Data collection	DNA sequencing base-calling was performed with Guppy v4.0.15, utilising the high-accuracy model template_r9.4.1_450bps_hac.jsn. SNP genotyping for cocoa varieties involved quantifying fluorescence intensity with Fluidigm EP1 software, with genotypic calls automatically generated using Fluidigm SNP Genotyping Analysis software v4.1.3.
Data analysis	Summary of data analysis software:<br><br>R v4.3.0 was used for statistical analyses, including functions from the base stats package. All figures were plotted with ggplot2 v3.4.2 unless otherwise stated.<br><br>Temperature, pH and bean colour analysis<br>Temperature and pH distributions were analysed using the stats v4.3.0 package in R, while correlation analyses utilized ggpubr v0.6.0. ImageJ v1.54d70 was used for extraction of RGB values, grayscale, and luminance from bean images. Bean colour variations and principal components were assessed using the prcomp function in R.<br><br>Microbiota community composition<br>The initial dataset underwent demultiplexing and trimming with qcat v1.1.0. Filtering was done with NanoFilt v2.8.0, and reads were mapped to reference genomes using minimap2 v2.17. Contaminant sequences were removed with SAMtools v1.9 and Seqtk v1.3. Kraken v2.1.2 classified sequencing reads, with relative abundances estimated using Bracken v2.7. Abundance and taxonomic tables were extracted with Pavian v1.0. Data processing and diversity analyses were conducted with phyloseq v1.44.0, microbiome v1.22.0, and microbiomeutilities v1.0.17. PERMANOVA, ordination, and other diversity metrics were calculated using vegan v2.6.4. agricolae v1.3.5 was used to separate means. Differential abundance was analysed with DESeq2 v1.40.0, while microbial source tracking was performed with FEAST v0.1.0.

Analysis of cocoa genotypes
SNP profiles were aligned using DECIPHER v2.24.0, and a distance matrix was generated with seqinr v4.2.16. A Neighbour-Joining tree was constructed using ape v5.6.2 and visualized with ggtree v3.8.0 and ggtreeExtra v1.10.0. Ancestry analysis was performed with STRUCTURE v2.3.4 and structure-threader v1.3.10, with congruence among runs assessed using CLUMPAK v1.1.

Bean quality and cocoa liquor sensory profiles
Bean quality was evaluated with stats v4.3.0, and variance in sensory attributes of cocoa liquors was estimated using vegan v2.6.4.

Linking abiotic and biotic features to sensory attributes
Temperature and pH kinetics were modelled using the PPFM 2020 (Practical Program for Forces Modeling) tool, with correlations calculated using Hmisc v5.0.1. gcplyr v1.5.2 was used to extract growth features of selected taxa. randomForest v4.7.1.1 identified key features linking temperature, pH, and microbial growth to sensory attributes.

Metagenome assembled genome (MAG) construction, classification, annotation and abundance
Contigs were assembled using metaFlye in Flye v2.9. Metagenomic binning was performed with minimap2 v2.17, SAMtools v1.12, MaxBin v2.2.4, and MetaBAT v2.15. MAGs were dereplicated with dRep v3.4.0 and evaluated using CheckM v1.1.6. Marker gene sequences for each MAG were aligned using Clustal W in msa v1.32.0. Alignments were trimmed with microseq v2.1.6, transformed into a distance matrix with seqinr v4.2.16, and a Neighbour-Joining tree was constructed with ape v5.6.2. The resulting tree was visualised using ggtree v3.8.0 with ggtreeExtra v1.10.0. Ribosomal RNA (rRNA) genes in MAGs were identified using Barrnap v0.9. Open reading frames in each MAG were predicted using FragGeneScanRs v1.1.0. Taxonomic classification was done with CAT v8.22, and functional annotation of predicted proteins was carried out using eggNOG-mapper v2.1.9, Diamond v2.0.11, and MMseqs2 release 12-113e3. Relative abundance of MAGs was determined using CoverM v0.6.1. Enrichment profiles were determined using DESeq2 v1.40.0.

Enrichment of microbial biological functions
Contigs were deduplicated using BBTools v38.76 and relative abundances were determined with minimap2 v2.17 and CoverM v0.6.1. Taxonomic classification was conducted using CAT v8.22, and DESeq2 v1.40.0 was employed for enrichment analysis. Open reading frames encoded within contigs were predicted using FragGeneScanRs v1.1.0 and functional annotation of predicted proteins was performed with eggNOG-mapper v2.1.9 with Diamond v2.0.11 and MMseqs2 release 12-113e3. GO enrichment analysis was determined with the GO_MWU tool.

Metabolic network modelling
Genbank-formatted files for MAGs were created using emapper2gbk v0.3.0, and metabolic networks were modelled with Metage2Metabo v1.5.3 with Pathway Tools v26.0. The output was visualized using ComplexHeatmap v2.12.1 with circlize v0.4.15, and ggvenn v0.1.10 was used for metabolite visualization.

Isolate identification
16S rRNA and ITS sequences were identified via BLAST v2.12.0.

Genome assembly, annotation and construction of metabolic network of isolates
Cutadapt v4.6 was used to filter and trim paired-end reads and de novo assembly of draft genomes for isolates were performed with SPAdes v3.15.5. Assembled genomes were evaluated using BUSCO v5.6.1. Open reading frames were predicted with FragGeneScanRs v1.1.0. Functional annotations were done with eggNOG-mapper v2.1.9, Diamond v2.0.11, and MMseqs2 release 12-113e3. Metabolic networks were modelled using Metage2Metabo v1.5.3 with Pathway Tools v26.0.

16S rRNA and ITS amplicon sequence processing
Amplicon data was demultiplexed and trimmed with Cutadapt v4.6, and subsequently processed using DADA2 v1.24.0. SILVA 138 and UNITE v9 databases were used for microbial taxonomic classification.

Analysis of volatile and non-volatile compounds in cocoa bean/liquor samples
Mass Profiler (MP) v10 and Profinder v10 were used for non-volatile compounds feature extraction and chromatogram alignment. Principal component analysis of volatile compounds was conducted with the prcomp function in R, with enrichment analysis performed using DESeq2 v1.40.0.

Scripts and additional source data required to reproduce the analyses of this study can be accessed through the following GitHub link: https://github.com/David-Lee86/min-com-choc.

For manuscripts utilizing custom algorithms or software that are central to the research but not yet described in published literature, software must be made available to editors and reviewers. We strongly encourage code deposition in a community repository (e.g. GitHub). See the Nature Portfolio guidelines for submitting code & software for further information.

Data

Policy information about availability of data

All manuscripts must include a data availability statement. This statement should provide the following information, where applicable:

- Accession codes, unique identifiers, or web links for publicly available datasets
- A description of any restrictions on data availability
- For clinical datasets or third party data, please ensure that the statement adheres to our policy

Nanopore sequencing data, as well as 16S rRNA and ITS amplicon sequencing data generated for this study, have been archived in the NCBI Sequence Read Archive under project accession PRJNA1104253. Microbial genomes and metagenomes produced in this work are available at https://www.gabrielcastrillo.com/ through the following repositories: Trinidad Isolate Genomes Repository and Colombia MAGs Repository. Datasets required to reproduce the results of this study are available in the associated GitHub repository: https://github.com/David-Lee86/min-com-choc. Reference genomes used include Theobroma cacao Criollo v2.0 (Cocoa Criollo B97-61/B2 version 2; https://cocoa-genome-hub.southgreen.fr/download) and Homo sapiens GRCh38.p14 (RefSeq GCF_000001405.40). The strain collection used in this study is available upon request by contacting Gabriel Castrillo (gabriel.castrillo@nottingham.ac.uk).

Research involving human participants, their data, or biological material

Policy information about studies with human participants or human data. See also policy information about sex, gender (identity/presentation), and sexual orientation and race, ethnicity and racism.

Reporting on sex and gender	Not applicable
Reporting on race, ethnicity, or other socially relevant groupings	Not applicable
Population characteristics	Not applicable
Recruitment	Not applicable
Ethics oversight	Not applicable

Note that full information on the approval of the study protocol must also be provided in the manuscript.

Field-specific reporting

Please select the one below that is the best fit for your research. If you are not sure, read the appropriate sections before making your selection.

☒ Life sciences ☐ Behavioural & social sciences ☐ Ecological, evolutionary & environmental sciences

For a reference copy of the document with all sections, see nature.com/documents/nr-reporting-summary-flat.pdf

Life sciences study design

All studies must disclose on these points even when the disclosure is negative.

Sample size

Field experiment: Farm selection and fermentation trials (design and rationale)
Three cocoa plantations, one each from Santander, Huila, and Antioquia, were selected for this study. Farm selection was primarily determined by:
Representation of key agroecological zones
Colombia's cocoa production spans diverse climates, soil types, and topographies, all of which can significantly influence fermentation dynamics and bean quality. A study by Calvo et al.1 highlighted the importance of including distinct agroecological regions, such as Santander, Huila, and Antioquia, to capture this environmental variability. These regions also represent the three most important cocoa-producing agroecological zones in Colombia.

Logistical feasibility and depth of data collection
Selecting three farms enabled detailed, repeated sampling (temperature, pH, microbial analyses) and practical management of resources (e.g., time, personnel, equipment). A similar approach is common in cocoa fermentation field studies aiming for in-depth multi-factorial analysis2-4

Best practices and infrastructure considerations
The selected farms adhered to best agricultural practices and had suitable fermentation infrastructure, ensuring consistency in fermentation procedures and data reliability.

Capturing Seasonal Variability in Cocoa Fermentation Dynamics
Cocoa bean fermentations were analysed during the two standard harvest periods of the year: the mid harvest (May) and the main harvest (October-November) on all three farms, except in Antioquia, where only the main harvest was characterised (n = 5 independent fermentations). This design allowed us to capture seasonal differences in climatic conditions such as rainfall, temperature, and humidity, that may influence pod maturity, pulp composition, microbial activity, and ultimately cocoa fermentation dynamics and bean quality. By including both mid and main harvests in the design, the study provides a more comprehensive understanding of fermentation variability across seasons, enhancing the relevance and generalizability of the findings.

Field experiment: Temperature and pH monitoring
The temperature of the fermenting cocoa mass was recorded daily at three different locations within the fermentation boxes (bottom left corner, middle, and top right corner) and at two depths. For pH measurement, beans were collected from the boxes, from three different locations within the fermentation boxes. Three beans were collected from each location and analysed. Daily measurements at three spatial points and two depths per fermentation box ensured spatial heterogeneity was captured. This granularity balances data richness with practical constraints (equipment and labour availability) and follows precedent in cocoa fermentation analysis5

Field experiment: Bean colour measurements
Images were taken daily to track the colour changes in the beans. From these images, colour dimensions were extracted by selecting at least eight points on each bean image at each analysed time point. Measuring at least eight points per bean image per time point provides robust data to capture intra-bean variability and temporal dynamics of colour changes.

Field experiment: Microbial community analyses
Samples for microbial community analysis were collected daily in duplicate from the fermenting beans during each independent fermentation at the three farms, Santander, Huila, and Antioquia, during both the mid harvest (May) and the main harvest (October-November). Additional duplicate samples were collected from various environmental sources on the farms. The sample size was chosen to balance logistical feasibility with the need for sufficient replication to account for both biological and technical variability. Duplicate sampling at each time point and from each source provided internal replication, enabling reliable detection of temporal changes and farm-specific differences in microbial community composition. The inclusion of sampling across two harvest periods and three distinct farms ensured adequate coverage of environmental and seasonal variability, thereby enhancing the relevance and generalizability of the findings. This replication strategy is consistent with established practices in cocoa fermentation studies2-4,6,7, and are considered sufficient to generate robust, generalisable insights while allowing meaningful statistical comparisons.

Field experiment: SNP genotyping cocoa varieties
A preliminary survey of the morphological diversity of the cocoa fruits was performed to estimate the number of cocoa varieties cultivated on the farms. Following this survey, genetic analysis of each distinct morphotype (n = 24) was performed.

Field experiment: Bean quality assessment
Quality assessments were performed on 100 g samples of beans from each fermentation batch (both mid and main harvests) at each farm. This sample size follows industry-standard practices for cocoa bean quality evaluation8, ensuring comparability with established benchmarks and commercial grading protocols.

In vitro experiment: Fermentation design and sampling
In vitro fermentations were performed in triplicate using a defined synthetic microbial consortium, with and without inoculum (serving as a negative control) to ensure statistical robustness. To monitor the microbial community dynamics, samples were collected and analysed at 0, 48, and 96 h, with at least duplicate samples for each fermentation replicate further strengthening the robustness and reliability of the experimental design. pH measurements of the testa/pulp and cotyledons were recorded daily from a single bean in each fermentation.

For the single-strain dropout experiment, four independent fermentations were carried out for each microbial consortium treatment (n = 52), providing strong replication and supporting statistical comparisons of dropout effects. pH measurements of the testa/pulp and cotyledons were again recorded daily from a single bean in each fermentation. Swab samples for microbial community analysis were collected at 0, 24, and 48 h (n = 156), and five beans from each treatment replicate were sampled at 0, 48, and 120 h for metabolomic analysis (n = 156).

Sampling at these defined time points (0, 24, 48, 96, and 120 h) was designed to capture key fermentation phases, providing sufficient resolution to characterise fermentation dynamics.h

Sensory evaluation of cocoa liquors
Cocoa liquor samples were subjected to sensory evaluation through coded, randomised tastings by 3-6 trained sensory panellists in duplicate or triplicate. This provided sufficient robustness for focused evaluations, ensuring reproducibility while managing panel fatigue

Total cell counts and growth curves
Total cell counts in the 9-member SYNCOM were measured from four independent fermentations at 0, 48, and 120 h (n = 12). Growth curves of individual isolates from the 9-member SYNCOM were constructed by culturing each isolate at four different pH levels and three different temperature conditions. A minimum of three independent cultures were performed for each isolate under each condition. The chosen sample sizes follow established standards for microbial growth studies, providing sufficient replication for robust statistical analysis and reliable characterization of growth dynamics.

Analysis of volatile and non-volatile compounds
A minimum of three replicates per liquor sample were analysed with randomized sample injections for the validation of the minimal community experiment, including 9-member SYNCOM-inoculated samples, No SYNCOM samples, Santander, Huila, Antioquia, and reference liquors. The quality of the headspace GC-MS runs was assessed by running the internal standard after every 5-20 consecutive sample runs and estimating the variations in retention time and peak areas. Using three or more replicates per liquor sample with randomized injections aligns with best practices in analytical chemistry, supporting repeatability and data reliability. For the single-strain dropout experiment, four independent biological fermentation replicates were conducted for each microbial consortium treatment (13 treatments) at 0, 48, and 120 h (n = 156). This level of replication provided sufficient statistical power for multivariate analysis and robust detection of differences between treatments.

Analysis of non-volatile compounds in cocoa beans
For each time point (0, 48, and 120 h), aliquots from replicates of each treatment were pooled separately, and five replicates per pooled sample were analysed with randomized sample injections (n = 195). The sample size was chosen to balance analytical throughput, resource constraints, and the need for sufficient replication to enable robust statistical comparisons. The use of five analytical replicates, combined with randomized injections, provides reliable estimates of technical variability and ensures repeatability, while supporting confident multivariate statistical analysis.

Reference
1 Calvo, A. M. et al. Dynamics of cocoa fermentation and its effect on quality. Sci. Rep. 11, 16746 (2021).
2 Pacheco-Montealegre, M. E., Dávila-Mora, L. L., Botero-Rute, L. M., Reyes, A. & Caro-Quintero, A. Fine resolution analysis of microbial communities provides insights into the variability of cocoa bean fermentation. Front. Microbiol. 11, doi:10.3389/fmicb.2020.00650 (2020).
3 Bortolini, C., Patrone, V., Puglisi, E. & Morelli, L. Detailed analyses of the bacterial populations in processed cocoa beans of different

geographic origin, subject to varied fermentation conditions. Int. J. Food Microbiol. 236, 98-106 (2016).
4 Papalexandratou, Z., Camu, N., Falony, G. & De Vuyst, L. Comparison of the bacterial species diversity of spontaneous cocoa bean fermentations carried out at selected farms in Ivory Coast and Brazil. Food Microbiol. 28, 964-973 (2011).
5 Tan, J., Balasubramanian, B., Sukha, D., Ramkissoon, S. & Umaharan, P. Sensing fermentation degree of cocoa (Theobroma cacao L.) beans by machine learning classification models based electronic nose system. Journal of Food Process Engineering 42, e13175 (2019).
6 Hamdouche, Y. et al. Impact of turning, pod storage and fermentation time on microbial ecology and volatile composition of cocoa beans. Food Res. Int. 119, 477-491 (2019).
7 Camu, N. et al. Dynamics and biodiversity of populations of lactic acid bacteria and acetic acid bacteria involved in spontaneous heap fermentation of cocoa beans in Ghana. Appl. Environ. Microbiol. 73, 1809-1824 (2007).
8 Sukha, D. A. The grading and quality of dried cocoa beans. In Drying and roasting of cocoa and coffee (eds Ching Lik Hii & Flávio Meira Borém) Ch. 5, 5, 89-139 (CRC Press, 2019).

Data exclusions

No data was excluded from the analysis

Replication

Field fermentation experiments
To characterise the progression of the cocoa bean fermentation and ensure the reproducibility of our observations, we systematically monitored the fermentation process on farms that follow the traditional practices of local farmers who use wooden boxes to ferment cocoa beans. The methods for harvesting cocoa pods, extracting beans, and setting up and conducting the fermentations were consistently applied across all farms. To account for possible variabilities across the wooden boxes, temperature measurements were performed at two depths in the fermenting cocoa mass, close to the surface and in the middle of the fermenting beans across three distinct positions (bottom left corner, middle, and top right corner) within the fermentation box. Beans were also sampled from these positions for pH measurements, with the pH meter being calibrated daily. Photographs of the beans were taken daily using the same device at approximately the same time each day to maintain consistency. For microbial community analysis, samples were collected daily from the same depth and position within the fermentation box. To further assess reproducibility, we conducted the fermentation analysis during two separate harvest periods (mid and main harvest seasons) on all farms, except in Antioquia, where only the main harvest was analysed. These protocols were uniformly applied across all three farms for each harvest period.

In vitro fermentation experiments
In vitro fermentations were conducted in triplicate using a defined synthetic microbial consortium, with parallel fermentations without inoculum as negative controls. To track microbial community dynamics, samples were collected at 0, 48, and 96 h, with at least duplicate samples per fermentation replicate. pH measurements of the testa/pulp and cotyledons were recorded daily from a single bean per replicate fermentation. For the single-strain dropout experiment, four independent fermentations were performed for each microbial consortium treatment. pH was measured daily from a single bean per fermentation replicate. Microbial swab samples were collected at 0, 24, and 48 h, while five beans per replicate fermentation were sampled at 0, 48, and 120 h for metabolomic analysis.

Total cell counts and growth curves
Total cell counts for the 9-member SYNCOM were determined from four independent fermentations at 0, 48, and 120 h. Growth curves of individual isolates were generated from at least three independent cultures per isolate, across four pH levels and three temperature conditions.

All replication attempts were successful and included in the study.

Randomization

Liquor samples were coded and subjected to randomized tastings by the trained sensory panellists. For the analysis of volatile and non-volatile compounds, sample replicates were randomly injected into the gas chromatography–mass spectrometry (GC-MS) and liquid chromatography-mass spectrometry (LC-MS) to ensure unbiased results. For the analysis of the microbial communities, the order of samples was randomized during the DNA extraction, library preparation, and sequencing steps.

Blinding

Samples were coded to blind the experimenter during microbial, volatile, and non-volatile compound analyses, and to blind sensory panellists during the evaluation of cocoa liquors.

Reporting for specific materials, systems and methods

We require information from authors about some types of materials, experimental systems and methods used in many studies. Here, indicate whether each material, system or method listed is relevant to your study. If you are not sure if a list item applies to your research, read the appropriate section before selecting a response.

Materials & experimental systems

n/a	Involved in the study	
☒	☐	Antibodies
☒	☐	Eukaryotic cell lines
☒	☐	Palaeontology and archaeology
☒	☐	Animals and other organisms
☒	☐	Clinical data
☒	☐	Dual use research of concern
☐	☒	Plants

Methods

n/a	Involved in the study	
☒	☐	ChIP-seq
☒	☐	Flow cytometry
☒	☐	MRI-based neuroimaging

Dual use research of concern

Policy information about dual use research of concern

Hazards

Could the accidental, deliberate or reckless misuse of agents or technologies generated in the work, or the application of information presented in the manuscript, pose a threat to:

No	Yes	
☒	☐	Public health
☒	☐	National security
☒	☐	Crops and/or livestock
☒	☐	Ecosystems
☒	☐	Any other significant area

Experiments of concern

Does the work involve any of these experiments of concern:

No	Yes	
☒	☐	Demonstrate how to render a vaccine ineffective
☒	☐	Confer resistance to therapeutically useful antibiotics or antiviral agents
☒	☐	Enhance the virulence of a pathogen or render a nonpathogen virulent
☒	☐	Increase transmissibility of a pathogen
☒	☐	Alter the host range of a pathogen
☒	☐	Enable evasion of diagnostic/detection modalities
☒	☐	Enable the weaponization of a biological agent or toxin
☒	☐	Any other potentially harmful combination of experiments and agents

Plants

Seed stocks	Cocoa beans analysed in this study were collected from commercial cocoa farms located in Santander, Huila and Antioquia Colombia. Cocoa beans analysed in the in-vitro study collected from the International Cocoa Genebank in Trinidad.
Novel plant genotypes	Not applicable
Authentication	Not applicable
